# Does Medication Status Impact the Effectiveness of Nuts in Altering Blood Pressure and Lipids? A Systematic Review and Meta-Analysis

**DOI:** 10.1093/nutrit/nuaf033

**Published:** 2025-04-01

**Authors:** Hoi Y Wong, Alison M Coates, Sharayah Carter, Alison M Hill

**Affiliations:** Alliance for Research in Exercise, Nutrition and Activity (ARENA), Allied Health and Human Performance, University of South Australia, Adelaide, SA 5001, Australia; Alliance for Research in Exercise, Nutrition and Activity (ARENA), Allied Health and Human Performance, University of South Australia, Adelaide, SA 5001, Australia; Alliance for Research in Exercise, Nutrition and Activity (ARENA), Allied Health and Human Performance, University of South Australia, Adelaide, SA 5001, Australia; School of Health and Biomedical Sciences, RMIT University, Bundoora, VIC 3083, Australia; Alliance for Research in Exercise, Nutrition and Activity (ARENA), Clinical and Health Sciences, University of South Australia, Adelaide, SA 5001, Australia

**Keywords:** cardiovascular diseases, blood pressure, cholesterol, nuts, medication

## Abstract

**Context:**

Nut consumption is attributed to improvements in risk factors for cardiovascular disease (CVD), including high blood pressure (BP) and dyslipidemia. However, it is unclear whether these effects are altered with concurrent treatment with BP and lipid-lowering medication.

**Objective:**

We sought to investigate the effects of the consumption of whole tree nuts and peanuts (collectively termed nuts) on BP and lipids, and whether BP and lipid-lowering medication use alters these effects.

**Data Sources:**

The MEDLINE, EMBASE, Scopus, and Web of Science databases were systematically searched through June 21, 2023, for randomized controlled trials (RCTs) assessing the effects of nut consumption on BP and/or lipids.

**Data Extraction:**

Random effects meta-analyses (mean difference, 95% confidence interval [CI]) were conducted, with subgroup analyses based on reported participant use of BP or lipid-lowering medication, including medicated, unmedicated, unreported (ie, use not specified), and mixed (ie, included combined data from medicated and unmedicated participants). A total of 115 studies were included in the review, of which 109 were meta-analysed.

**Data Analysis:**

Nut consumption significantly reduced triglycerides (TG), total cholesterol, low-density lipoprotein cholesterol, very-low-density lipoprotein cholesterol, non–high-density lipoprotein cholesterol, and apolipoprotein B, with no effect on high-density lipoprotein cholesterol or blood pressure. Few studies were conducted in medicated participants only (n = 1 for lipid outcomes only), and for the studies including both medicated and unmedicated participants (ie, mixed), outcomes by medication use were not reported. Significant differences in TG and apolipoprotein B were observed between medication use groups, with nut consumption resulting in the largest reductions in unmedicated participants. Strong heterogeneity was observed with no evidence of publication bias.

**Conclusions:**

Lipid-lowering, but not BP-lowering benefits of nut consumption were observed; however, few studies reported the effect based on participants’ medication status. Future studies are required to determine if there are additional benefits of including nuts in the diet of medicated patients with cardiovascular disease.

**Systematic Review Registration:**

PROSPERO registration code CRD42022296849.

## INTRODUCTION

Poor dietary habits contribute to the development of cardiovascular disease (CVD).^1^ Lifestyle modifications, including dietary change, play a fundamental role in preventing or reducing CVD risk factors such as high blood pressure (BP) and dyslipidemia, which may be associated with increased levels of triglycerides (TG), total cholesterol (TC), and low-density lipoprotein cholesterol (LDL-C) and reduced levels of high-density lipoprotein cholesterol (HDL-C).^2–6^ While advice regarding lifestyle change is recommended for all persons with elevated blood pressure and cholesterol,^4^^,^^7^ pharmacological treatment may be required to improve cardiovascular outcomes.

Nuts, often recommended as an integral part of a healthy balanced diet, are featured prominently in food-based dietary guidelines worldwide.^8^ Tree nuts and peanuts are nutrient dense and rich in unsaturated fats, including monounsaturated fatty acids (MUFAs) and polyunsaturated fatty acids (PUFAs), as well as protein, fiber, vitamins (eg, folic acid, niacin, tocopherols, and vitamin B6), minerals (eg, calcium, magnesium, potassium), antioxidants, phytochemicals, and phytosterols.^4^^,^^9^ These nutrients and nuts as a food have been associated with lipid-lowering benefits via several proposed mechanisms, such as reduction in bile acid absorption and increase in cholesterol catabolism, and increased fecal excretion of fat, cholesterol, and bile acids via interference with micelle formation in the intestinal lumen.^10^ Similarly to statins, which are the predominant antidyslipidemic medication, nuts may lower cholesterol via inhibition of 3-hydroxy-3-methylglutaryl-CoA reductase, which increases the synthesis by gut microbiota of short-chain fatty acids (SCFAs), particularly butyrate and propionate.^10^^,^^11^ Nuts may increase PUFA incorporation in lipid bilayers, leading to physical changes in the membrane—including increased fluidity, flexibility, and elasticity and reduced thickness,^12^ which can modulate the activity of membrane receptors and their ligands, eg, increasing the affinity of LDL receptors for apolipoprotein B (ApoB)-100 in LDL particles.^10^ Proposed mechanisms by which nuts may lower BP also relate to their nutrient content: PUFAs may increase baroreceptor sensitivity, arginine may facilitate vasodilation effects, magnesium may act as a natural calcium channel blocker, and fiber may improve gut microbiota and increase synthesis of SCFAs, particularly acetate, to facilitate the reduction in BP.^11^^,^^13^ Some, but not all, of these mechanisms overlap with those of antihypertensive medications, which act as angiotensin-converting enzyme inhibitors, α1-blockers, cardio-selective β-blockers, calcium channel blockers, and diuretics, with both single and combination medication therapies commonly used.^14^

Systematic reviews and meta-analyses of prospective cohort studies have consistently demonstrated positive associations between increased nut consumption and reduced CVD incidence and/or mortality.^15^^,^^16^ A systematic review and dose–response meta-analysis of 12 prospective studies reported that a daily intake of 28 g of nuts was associated with a 21% reduced risk for CVD compared to no nut consumption.^17^ A recent meta-analysis of 139 randomized controlled trials (RCTs) reported that tree nut and peanut consumption led to significant reductions in TG, TC, LDL-C, and ApoB but had no effect on HDL-C, systolic BP (SBP), or diastolic BP (DBP).^18^

While nut intake has proven effective in reducing the risk of CVD,^19^ to our knowledge there has been no previous systematic review that has specifically compared the impact of nut consumption on BP and lipids in patient populations with and those without concurrent medication use for these conditions. Given that a wide range of food compounds can alter the pharmacodynamic effects of some medications,^20^^,^^21^ it is possible that there may be interactions between nutrients in nuts and the medications used to manage hyperlipidemia and hypertension.^22^

The lack of consideration of medication use may mean that previous reports of cardioprotective effects of nuts^18^ may have included findings that had been blunted, unaffected, or perhaps elevated depending on the medication status of the study participants and potential antagonistic or synergistic mechanisms of action of antilipidemic and antihypertensive medications and nuts. As such, the primary objective of this review was to provide a comprehensive summary of the effects of nut consumption on BP and lipid regulation while exploring the potential influence of medication use on these effects.

## METHODS

This systematic review and meta-analysis was conducted in accordance with the 2020 Preferred Reporting Items for Systematic Reviews and Meta-Analyses (PRISMA) model (Table S1).^23^ The protocol was registered in the Prospective Register of Systematic Reviews (PROSPERO) (CRD42022296849).

### Search Strategy and Study Selection

Searches were performed on the Medline, EMBASE, Web of Science, and Scopus databases from inception through March 7, 2022, and rerun on June 21, 2023. Relevant keywords or search terms were sourced from previous systematic reviews on nuts, lipids, and BP.^2^^,^^5^^,^^24^ These keywords were pilot tested in a search via Medline to determine whether they identified a set of clearly eligible studies. The search strategy was refined, and a consensus was reached among the research team. An academic librarian (University of South Australia) reviewed the final search strategy for all databases (Table S2). Searches were limited to studies in humans and English-language articles only. Reference lists of relevant articles and systematic reviews were manually searched for articles that may have been missed by the database searches.

Once duplicates were removed, all records identified by the search strategy were imported into Covidence (Covidence Systematic Review software; Veritas Health Innovation), where the titles and abstracts were independently screened based on the inclusion and exclusion criteria (H.W. screened all studies while A.M.C., A.M.H., and S.C. equally divided the role of the second reviewer). After consensus was reached and ineligible studies were removed, the full texts of the included studies were independently screened by the same 4 reviewers (H.W. screened all of the studies while A.M.C., A.M.H., and S.C. equally divided the role of the second reviewer). Any discrepancies were resolved by a third reviewer who was not involved in the initial review (ie, A.M.C., A.M.H., or S.C.).

When the same outcomes of interest from 1 study were reported in multiple articles, the article with the longest follow-up time and the largest sample size was included. This approach was used to prevent duplication of study populations in the meta-analysis.

### Eligibility Criteria

Detailed PICOS (Participants, Intervention, Comparators, Outcomes, and Study Design) criteria were used to define the research question: “In medicated and unmedicated humans, does a nut-containing diet, compared to a nut-free diet, improve blood lipids or BP over a minimum duration of 3 weeks?” and are presented in Table 1. Briefly, RCTs of human studies with an intervention duration of ≥3 weeks comparing the effects of a nut-containing diet (tree nuts or peanuts) with those of a nut-free control diet on one of the outcomes of blood lipids or BP were included (Table 1). A minimum duration of 3 weeks was selected to exclude postprandial studies and enable sufficient time for diet-induced changes in lipids and BP to occur.^25^ This criterion is consistent with a recent meta-analysis investigating the effect of nuts on BP, lipids, and lipoproteins.^18^ Only studies investigating the effects of whole nuts or peanuts were included in the review; studies involving nut products, such as nut oils, flours, or powders, were excluded. Studies that included manipulation of energy intake along with nut consumption were included if an equivalent no-nut comparison was available. Studies that were not available in a full-text version or were not published in a peer-reviewed journal in the English language were excluded.

**Table 1.** Description of the PICOS standards used to define the research questions, and inclusion and exclusion criteria.

Parameters	Descriptions
Participants	Medicated and unmedicated humans
Interventions	Nut-containing diet (including dietary co-interventions such as energy restriction) Intervention diets containing whole nuts (including roasted and salted nuts) of at least 1 of the tree nuts (eg, almonds, walnuts, hazelnuts, pistachios, Brazil nuts, pecans, cashews, macadamia, pine nuts) or peanuts
Comparisons	Nut-free control diet (with equivalent dietary co-intervention as relevant)
Outcomes	Change in blood lipid and/or BP parameters Reported at least 1 of the outcomes of blood lipids (TC, TG, LDL-C, HDL-C, non-HDL-C, VLDL-C, IDL-C, ApoA1, Apo B, and Lp[a]) or BP (SBP and DBP)
Study Design	Randomized controlled trials (parallel or crossover design) Intervention duration was equal to or greater than 3 weeks

Abbreviations: Apo, apolipoprotein; DBP, diastolic blood pressure; HDL-C, high-density lipoprotein—cholesterol; IDL-C, intermediate-density lipoprotein—cholesterol; LDL-C, low-density lipoprotein—cholesterol; Lp, lipoprotein; SBP, systolic blood pressure; TC, total cholesterol; TG, triglycerides; VLDL-C, very low-density lipoprotein—cholesterol.

### Data Extraction

A data extraction template was developed and pilot tested on 4 randomly selected articles. The template was refined, and a consensus was reached among all reviewers. One reviewer (H.W.) extracted the following information from each of the included studies: study author, publication year, country, study design (crossover or parallel), intervention duration, intervention (nut type, form, and dose), control and outcome measures (BP and lipid parameters), and participant characteristics (including number randomized and number who completed the study, age, gender, body mass index [BMI], general health conditions, and antidyslipidemia and antihypertension medication use). Data related to other common medications such as antidiabetic drugs or antidepressants were not extracted. Only the use of antidyslipidemic and antihypertensive medications was of interest in this review, and on the basis of these variables the participants were further categorized into 4 subgroups: (1) Medicated, all participants in the trial were medicated; (2) Unmedicated, no participants in the trial were medicated; (3) Mixed, both medicated and unmedicated participants were included in the trial; however, their results were not reported separately; (4) Unreported, use of medication was not reported. On one occasion participant characteristic data were sourced from Ghanavati et al.,^26^ but the relevant outcomes were obtained from Ghanavati et al.^27^ Outcome data were checked by a second reviewer (A.M.H. and S.C.).

### Quality Assessment

All eligible articles were assessed in duplicate by 2 independent reviewers (H.W. screened all studies while A.M.C., A.M.H., and S.C. equally divided the role of the second reviewer) using the American Dietetic Association Quality Criteria.^28^ When there was a discrepancy in the final rating, each component was re-reviewed by a third reviewer who was not involved in the initial review (ie, A.M.C., A.M.H., or S.C.) to reach a consensus. A final rating of either “positive” (all answers to the relevance questions are “yes” and most answers to the validity questions, including the 4 criteria—2, 3, 6, and 7, and 1 additional question, are “yes”, “neutral” (if any answer to the 4 criteria in the validity questions is “no” but other criteria indicate strengths) or “negative” (if 6 or more answers to the validity questions are “no”) was given. A negative rating was used to exclude studies with poor quality from meta-analyses. The certainty of the body of evidence for all outcomes was rated using the Grading of Recommendations, Assessment, Development, and Evaluations (GRADE) framework (GRADEpro Guideline Development Tool [Software]) against the criteria of study design, risk of bias, inconsistency, indirectness, imprecision, and other considerations including publication bias.^29^

### Data Synthesis

*Parallel Studies.* For each outcome, the means and SDs of the changes for the intervention and control groups were extracted. When not reported, the pre- and postintervention means were used to calculate the mean changes for each group. The SD of the change was calculated using the process outlined in the Cochrane Handbook ^30^ using either the median correlation coefficient (*R*) for each outcome measure (calculated from studies that reported baseline, post, and change values), or a modest correlation coefficient of 0.5 when no correlation estimates were available.

*Crossover Studies.* The mean within-individual difference (MD) between the intervention and control phases and associated SE were extracted. When this value was not reported, the mean final values and SDs for the control and intervention phases were extracted. These values were used to calculate the MD (intervention − control), SD, and SE using the process outlined in the Cochrane Handbook.^31^ The median correlation coefficient for each outcome (calculated from studies that reported final values for the intervention and control phases and MD) was used to calculate the SD of the difference, or a modest correlation coefficient of 0.5 was imputed when no correlation estimates were available.

When the SE or 95% confidence interval (CI) was reported, the SD was calculated using formulas in the Cochrane Handbook.^30^ When medians and 25th and 75th percentiles were reported, the formulas from Wan et al (2014)^32^ were used to calculate means and SDs. Geometric means (with associated variance) were converted to arithmetic means and SDs using the approach by Higgins et al.^33^ When results for both intention-to-treat analysis and per-protocol analysis were reported, the former were extracted. When the non-adjusted and adjusted results were reported, only the adjusted results were extracted. Data were converted to SI units, for example, milligrams per deciliter was converted to millimoles per liter by dividing by 88.57 for TG and 38.57 for TC, LDL-C, HDL-C, VLDL-C, intermediate-density lipoprotein cholesterol (IDL-C), and non-HDL-C. While the most appropriate units of measurement of lipoprotein a [Lp(a)] are nanomoles per liter,^34^ most studies reported Lp(a) concentrations in milligrams per liter, based on the isoform of Lp(a) that was detected in the assay; therefore grams per liter was used as the preferred unit of measurement. To calculate nut dose (in grams per day) from studies reporting nut intake as a percentage of total energy intake, the mean energy intake of participants was multiplied by the percent of total energy from nuts and divided by the energy per gram of nuts. The energy values for different nut types were obtained from the United States Department of Agriculture National Nutrient Database.^35^

To avoid unit of analysis errors, unless implementing a subgroup analysis, data from studies with multiple arms were combined using the formula in the Cochrane Handbook.^30^ This approach included studies investigating different nut doses, nut types, or nut variants (for example, with or without skin, high-oleic vs conventional) within the 1 study. Data from 1 study investigating different timings of nut consumption were combined because the volume of nuts was the same for each treatment and there was no difference in outcomes between the treatments.^36^ Similarly, data from the ad libitum and substitution arms in the study by Guarneiri, Paton, and Cooper^37^^,^^38^ were combined because there were no reported differences in energy intake, nut intake, or outcomes between arms. Studies that compared different nut interventions to their own control (ie, 4 arms)^39^^,^^40^ were treated separately. When the article did not provide sufficient data for meta-analysis, the study authors were contacted for this information. If the authors did not reply after 2 months (they were emailed twice), these articles were excluded from the meta-analysis and only reported descriptively.

### Statistical Analysis

Random effects meta-analyses were performed using Stata version 18 (StataCorp LLC). To avoid unit of analysis error, MD and SE estimates for parallel studies were first calculated in Stata and subsequently combined with precalculated MD and SE values for crossover studies. A separate random-effects model (primarily restricted maximum likelihood [REML] or maximum likelihood [ML]) was then implemented to determine the MD with the 95% CI for each review outcome overall and by medication use, which was the primary analysis for this review. A meta-analysis was performed for all overall outcomes with 2 or more separate studies.^41^ For medication use, meta-analyses were only performed for outcomes containing at least 10 studies to enable sufficient distribution across medication use groups.

The presence of statistical heterogeneity within each meta-analysis was investigated using the Cochran’s Q and *I*^2^ statistics. Heterogeneity was categorized as low, moderate, or high based on values of 25%, 50%, and 75%, respectively.^42^ Publication bias and small study effects were assessed via visual inspection of funnel plots (for outcomes with 10 or more studies)^41^ and Egger’s regression test, with *P  <  *.05 considered evidence of publication bias.

Subgroup analyses were conducted based on study design, duration (<12 weeks or ≥12 weeks), nut type, nut dose (≤30, 31–60, or ≥60 g/d), and health characteristics consistent with previous meta-analyses.^18^^,^^43^ Due to increasing interest in the use of nuts for weight management,^43^^,^^44^ differences in studies that implemented concurrent energy restriction with nut intake compared to energy restriction alone were also investigated. Subgroup analyses had a minimum of 10 studies for each outcome^45^; however, the number of studies within each subgroup may have been <10.

## RESULTS

**Figure 1.**
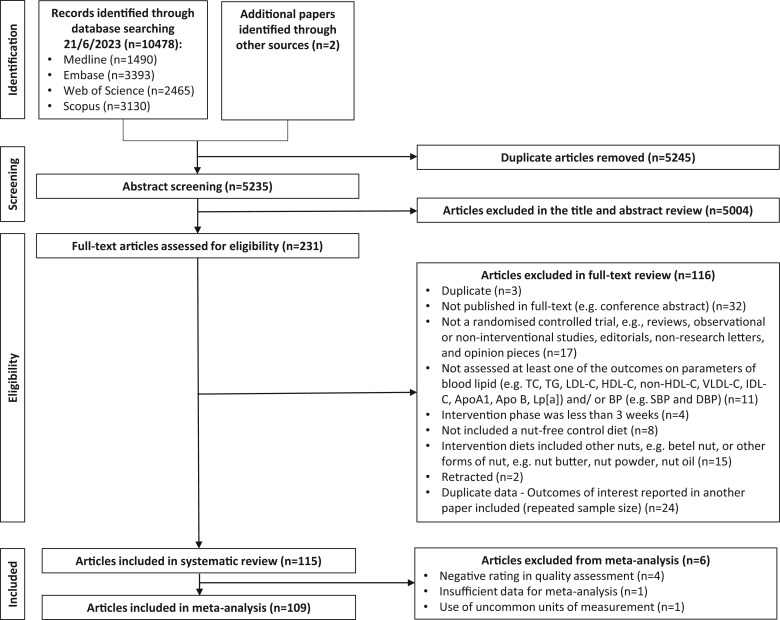
Flow diagram of the literature search process. Abbreviations: Apo, apolipoprotein; BP, blood pressure; DBP, diastolic blood pressure; HDL-C, high-density lipoprotein cholesterol; IDL-C, intermediate-density lipoprotein—cholesterol; LDL-C, low-density lipoprotein cholesterol; Lp, lipoprotein; SBP, systolic blood pressure; TC, total cholesterol; TG, triglyceride; VLDL-C, very low-density lipoprotein cholesterol.

A total of 10,478 articles were identified from the systematic search, with an additional 2 articles identified from the reference lists of included studies. The study selection process was conducted in accordance with the 2020 PRISMA Flow Diagram in Figure 1.^23^ After removal of duplicates with screening by title, abstract, and full -text, 115 articles describing 108 RCTs (including 65 parallel and 43 crossover designs) were included in the systematic review. Six articles were excluded from the meta-analysis due to insufficient data,^46^ use of uncommon units of measurement,^47^ or a negative quality rating,^48–51^ resulting in 109 articles describing 104 RCTs with 13,493 participants included in the meta-analysis (Figure 1).

### Study Characteristics

Key characteristics of the 115 articles included in the systematic review are presented in Table S3, including 6 articles^46–51^ that were excluded from the meta-analysis due to insufficient data or negative ratings. Interventions were conducted in 18 countries across 6 continents and ranged in duration from 3^52^ to 208 weeks,^53^ with only 8 studies (7%) having a duration of >1 year. The sample size of these interventions ranged from 9^51^ to 4808 participants,^53^ and the age of participants ranged from 10.8^54^ to 71.3 years.^55^ Among these interventions, 97 (84%) included both female and male participants, 8 included females only, 5 included males only, and 4 did not report gender distribution. The interventions included participants who were generally healthy^36^^,^^38^^,^^52^^,^^56–73^ or had various risk factors for chronic diseases, such as overweight/obesity,^37^^,^^74–95^ elevated TC/elevated LDL-C/hyperlipidaemia/hypercholesterolemia/dyslipidaemia,^47^^,^^48^^,^^50^^,^^51^^,^^54^^,^^96–111^ elevated fasting glucose/high risk of diabetes/prediabetes,^40^^,^^112–118^ or were diagnosed with type 2 diabetes mellitus,^39^^,^^49^^,^^119–127^ at risk of developing CVD,^53^^,^^128–131^ diagnosed with CVD,^132–134^ at risk or met criteria for metabolic syndrome,^135–142^ had chronic kidney disease,^55^ or had a mixture of health conditions and other conditions.^26^^,^^46^^,^^143–154^

Various types of tree nuts were consumed by study participants, including almonds (*n* = 29),^36^^,^^39^^,^^46^^,^^57^^,^^59^^,^^60^^,^^62^^,^^69^^,^^70^^,^^75^^,^^79^^,^^80^^,^^84^^,^^87^^,^^90^^,^^93^^,^^94^^,^^101^^,^^109^^,^^114^^,^^115^^,^^117^^,^^118^^,^^128^^,^^132^^,^^134^^,^^145^^,^^147^^,^^149^ Brazil nuts (*n* = 1),^89^ cashews (*n* = 3),^111^^,^^119^^,^^126^ hazelnuts (*n* = 5),^54^^,^^64^^,^^83^^,^^125^^,^^144^ macadamias (*n* = 2),^153^^,^^154^ pecans (*n* = 7),^37^^,^^38^^,^^66^^,^^68^^,^^88^^,^^100^^,^^133^ pistachios (*n* = 16),^47^^,^^49^^,^^51^^,^^52^^,^^67^^,^^74^^,^^97^^,^^98^^,^^102^^,^^105^^,^^112^^,^^120^^,^^121^^,^^127^^,^^136^^,^^139^ and walnuts (*n* = 29),^40^^,^^48^^,^^50^^,^^55^^,^^56^^,^^58^^,^^61^^,^^65^^,^^71–73^^,^^77^^,^^81^^,^^82^^,^^86^^,^^96^^,^^99^^,^^107^^,^^108^^,^^110^^,^^123^^,^^124^^,^^137^^,^^140^^,^^143^^,^^148^^,^^150–152^ as well as peanuts (*n* = 7).^78^^,^^85^^,^^91^^,^^103^^,^^113^^,^^116^^,^^138^ Fourteen articles reported investigations of the effects of consuming mixed nuts,^27^^,^^53^^,^^63^^,^^76^^,^^92^^,^^95^^,^^104^^,^^122^^,^^129–131^^,^^141^^,^^142^^,^^146^ and 2 articles reported comparisons different types of nuts within one intervention, specifically, cashews and walnuts,^135^ and almonds and walnuts.^106^ Nut doses ranged from 10^134^ to 100 g^101^ per day across studies (the most frequently tested dosage was 30–60 g/d, 65% of studies). Six studies^60^^,^^70^^,^^83^^,^^97^^,^^98^^,^^139^ compared multiple doses of nuts and 4 studies compared different nut varieties (Pakistani vs American almonds^134^; skinned vs whole nuts^54^^,^^85^; conventional vs high-oleic peanuts^91^). Energy restriction featured as a co-intervention in 14 studies.^27^^,^^67^^,^^74^^,^^77^^,^^79^^,^^81^^,^^82^^,^^85^^,^^87^^,^^90–92^^,^^116^^,^^129^

When considering antihypertensive medication use, there were no articles that reported recruiting only participants who were Medicated. Twenty articles reported participants categorized as Mixed.^27^^,^^53^^,^^55^^,^^61^^,^^75^^,^^86^^,^^107^^,^^109^^,^^112^^,^^116^^,^^119^^,^^122^^,^^123^^,^^131^^,^^132^^,^^135^^,^^137^^,^^146^^,^^149^^,^^154^ The proportion of participants who received antihypertensive medication ranged from 4% to 87%; however, 3 articles did not report this detail.^135^^,^^149^^,^^154^ None of the Mixed medication studies reported results separately based on medication status (ie, Medicated and Unmedicated). There were 26 articles^36^^,^^47^^,^^48^^,^^50–52^^,^^57–59^^,^^68^^,^^71^^,^^73^^,^^76^^,^^88^^,^^89^^,^^97^^,^^98^^,^^102^^,^^103^^,^^105^^,^^113^^,^^120^^,^^127^^,^^140^^,^^150^^,^^151^ reporting results from Unmedicated participants. Sixty-nine articles^37–40^^,^^46^^,^^49^^,^^54^^,^^56^^,^^60^^,^^62–67^^,^^69^^,^^70^^,^^72^^,^^74^^,^^77–85^^,^^87^^,^^90–96^^,^^99–101^^,^^104^^,^^106^^,^^108^^,^^110^^,^^111^^,^^114^^,^^115^^,^^117^^,^^118^^,^^121^^,^^124–126^^,^^128–130^^,^^133^^,^^134^^,^^136^^,^^138^^,^^139^^,^^141–145^^,^^147^^,^^148^^,^^152^^,^^153^ did not report whether participants were Medicated or not and were therefore categorized as Unreported.

In terms of the use of antidyslipidemic medications, only 1 article reported outcomes from Medicated participants.^101^ There were 24 articles with Mixed medication use,^27^^,^^53^^,^^55^^,^^61^^,^^75^^,^^77^^,^^81^^,^^82^^,^^86^^,^^109^^,^^112^^,^^114^^,^^119^^,^^120^^,^^122^^,^^123^^,^^127^^,^^131–133^^,^^135^^,^^137^^,^^145^^,^^146^ and the proportion of participants who received antidyslipidemic medication ranged from 2.1% to 96%; however, 1 article did not report the proportion of medicated participants.^135^ None of the Mixed medication studies reported results separately based on medication status (ie, medicated and unmedicated). There were 56 articles^36–39^^,^^46–48^^,^^50–52^^,^^54^^,^^56–60^^,^^62^^,^^65–68^^,^^71^^,^^73^^,^^76^^,^^79^^,^^80^^,^^87–91^^,^^93^^,^^96–100^^,^^102–107^^,^^111^^,^^113^^,^^138^^,^^140^^,^^143^^,^^144^^,^^147^^,^^149–154^ involving Unmedicated participants, and 34 articles^40^^,^^49^^,^^63^^,^^64^^,^^69^^,^^70^^,^^72^^,^^74^^,^^78^^,^^83–85^^,^^92^^,^^94^^,^^95^^,^^108^^,^^110^^,^^115–118^^,^^121^^,^^124–126^^,^^128–130^^,^^134^^,^^136^^,^^139^^,^^141^^,^^142^^,^^148^ did not report whether participants were Medicated or not and were therefore categorized as Unreported.

### Quality Assessment

The quality ratings for the articles included in the systematic review are individually presented in Table S3. Overall, 47 articles were rated as “positive,” 63 articles were rated as “neutral,” and 5 articles were rated as “negative,”^48–51^^,^^98^ with most of the latter category excluded from the meta-analysis. However, 1 article^98^ that achieved a negative rating was included in the meta-analysis as an exception because it reported BP data from an intervention that had been well described in an earlier publication that reported lipid data^97^ and had achieved a positive rating and was included in the meta-analysis.

The certainty of the body of evidence that determined the effects of nut consumption on BP and lipid outcomes was assessed using the GRADE framework (Table S4). The certainty of evidence for DBP, VLDL-C, Apo A1, and Lp(a) was “high.” The body of evidence was downgraded to “moderate” for SBP, TG, TC, LDL-C, HDL-C, IDL-C, non-HDL-C, and Apo B due to inconsistency for these outcomes and for IDL-C due to imprecision (small sample size).

### Meta-Analysis

#### Blood Pressure—SBP and DBP

Two articles identified in the systematic review were excluded from the meta-analysis of BP due to insufficient data^46^ and poor quality,^49^ including 1 study of participants with Unreported medication status that reported a significant decrease in SBP compared with the control.^49^ Blood pressure (SBP and DBP) was evaluated in 59 strata from 58 interventions, with 1 additional intervention reporting only SBP (60 strata from 59 studies). Nut consumption did not result in lower participant SBP or DBP compared to controls **(**Table 2, Figures S1 and S2). Further analyses indicated that medication use does not modify the effect of nuts on SBP or DBP; however, no studies of Medicated participants only were included **(**Table 3). Subgroup analyses indicated significant differences related to health status for SBP; however, the small number of studies within the “Prediabetic” and “high risk of CVD” subgroups limits interpretation. Significant differences by nut type were observed for DBP, with larger reductions with almond consumption, although the magnitude of effect was small (Tables S5 and S6).

**Table 2.** Effect of nut consumption on blood pressure and lipids.

Outcomes	Number of strata	Number of studies	Number of participants (intervention: Control)	Effect estimates (95% CI), *P*-value	I^2^ (%)	Publication bias (95% CI), *P*-value
SBP, mmHg	60	59	9837 (5447: 5316)	−0.70 (−1.59, 0.19), *P* = .124	74	0.17 (−0.87, 1.21), *P = *.754
DBP, mmHg	59	58	9777 (5417: 5286)	−0.30 (−0.66, 0.06), *P = *.106	24	−0.04 (−0.55, 0.63), *P = *.894
TG, mmol/L	100	99	7715 (4844: 4535)	−0.06 (−0.08, −0.04), *P < *.001	96	−0.24 (−0.65, 0.17), *P = *.252
TC, mmol/L	101	99	7083 (4552: 4255	−0.12 (−0.17, −0.08), *P < *.001	89	−0.16 (−0.97, 0.64), *P = *.689
LDL-C, mmol/L	101	99	7114 (4561: 4277)	−0.11 (−0.14, −0.08), *P < *.001	90	−0.08 (−0.70, 0.53), *P = *.785
HDL-C, mmol/L	101	99	7689 (4848: 4565)	0.01 (−0.00, 0.03), *P = *.072	98	−0.08 (−0.71, 0.55), *P = *.798
VLDL-C, mmol/L	24	24	1792 (1226:1145)	−0.04 (−0.06, −0.02), *P < *.001	42	−0.47 (−1.40, 0.46), *P = *.322
IDL-C, mmol/L	5	5	266 (189: 190)	−0.02 (−0.04, 0.01), *P = *.135	40	−2.59 (−5.02, −0.17), *P = *.036
Non-HDL-C, mmol/L	21	21	1480 (1084: 981)	−0.20 (−0.27, −0.14), *P < *.001	73	0.09 (−1.08, 1.26), *P = *.885
Apo A1, g/L	28	28	1851 (1223: 1178)	−0.00 (−0.01, 0.01), *P = *.395	30	0.30 (−0.47, 1.06), *P = *.445
Apo B, g/L	34	34	2344 (1663: 1609)	−0.03 (−0.05, −0.02), *P < *.001	76	0.50 (−0.48, 1.48), *P = *.320
Lp(a), g/L	12	12	1193 (806: 816)	0.00 (0.00, 0.01), *P = *.023	0	−0.35 (−1.08, 0.39), *P = *.355

Abbreviations: Apo, apolipoprotein; CI, confidence interval; DBP, diastolic blood pressure; HDL-C, high-density lipoprotein—cholesterol; IDL-C, intermediate-density lipoprotein—cholesterol; LDL-C, low-density lipoprotein—cholesterol; Lp, lipoprotein; SBP, systolic blood pressure; TC, total cholesterol; TG, triglycerides; VLDL-C, very low-density lipoprotein—cholesterol.

**Table 3.** Effect of nut consumption on blood pressure and lipid outcomes based on medication status.

Outcomes	Medication status	Number of strata	Number of studies	Effect estimate (95% CI), *P*-value	Inconsistency *I*^2^ (%)	Test for subgroup differences
SBP, mmHg	Medicated	0	0	NA	NA	Chi² = 0.84, df = 2 (*P* = .656), *I*^2^ = 0%
	Mixed	17	17	−1.48 (-3.52, 0.56), *P = *.156	86	
	Unmedicated	12	12	−0.72 (-2.07, 0.62), *P = *.292	44	
	Unreported	31	30	−0.35 (-1.63, 0.92), *P = *.585	63	
DBP, mmHg	Medicated	0	0	NA	NA	Chi² = 0.91, df = 2 (*P* = .634), *I*^2^ = 0%
	Mixed	17	17	−0.08 (-0.70, 0.54), *P = *.795	36	
	Unmedicated	12	12	−0.51 (-1.24, 0.22), *P = *.173	21	
	Unreported	30	29	−0.41 (-0.99, 0.18), *P = *.176	8	
TG, mmol/L	Medicated	1	1	−0.14 (-0.34, 0.06), *P = *.174	NA	Chi² = 12.26, df = 3 (*P* = .007), *I*^2^ = 76%
	Mixed	22	22	−0.05 (-0.09, -0.00), *P = *.033	59	
	Unmedicated	46	46	−0.09 (-0.11, -0.06), *P < *.001	89	
	Unreported	31	30	−0.02 (-0.05, 0.01), *P = *.109	1	
TC, mmol/L	Medicated	1	1	−0.20 (-0.46, 0.07), *P = *.144	NA	Chi² = 3.16, df = 3 (*P* = .367), *I*^2^ = 5%
	Mixed	20	20	−0.08 (-0.14, -0.02), *P = *.007	51	
	Unmedicated	48	47	−0.16 (-0.22, -0.09), *P < *.001	95	
	Unreported	32	31	−0.10 (-0.18, -0.03), *P = *.005	71	
LDL-C, mmol/L	Medicated	1	1	−0.19 (-0.39, 0.02), *P = *.075	NA	Chi² = 5.39, df = 3 (*P* = .145), *I*^2^ = 44%
	Mixed	21	21	−0.07 (-0.11, -0.02), *P = *.005	49	
	Unmedicated	48	47	−0.14 (-0.18, -0.09), *P < *.001	96	
	Unreported	31	30	−0.08 (-0.12, -0.04), *P < *.001	26	
HDL-C, mmol/L	Medicated	1	1	0.07 (-0.05, 0.19), *P = *.267	NA	Chi² = 2.43, df = 3 (*P* = .489), *I*^2^ = 0%
	Mixed	22	22	0.01 (-0.02, 0.04), *P = *.478	91	
	Unmedicated	48	47	0.02 (-0.00, 0.04), *P = *.080	99	
	Unreported	30	29	0.00 (-0.02, 0.02), *P = *.845	76	
VLDL-C, mmol/L	Medicated	1	1	−0.08 (-0.14, -0.01), *P = *.031	NA	Chi² = 2.06, df = 3 (*P* = .561), *I*^2^ = 0%
	Mixed	2	2	−0.02 (-0.07, 0.03), *P = *.463	39	
	Unmedicated	14	14	−0.04 (-0.07, -0.02), *P < *.001	46	
	Unreported	7	7	−0.06 (-0.11, 0.00), *P = *.054	49	
Non-HDL-C, mmol/L	Medicated	1	1	−0.26 (-0.62, 0.10), *P = *.151	NA	Chi² = 3.71, df = 3 (*P* = .295), *I*^2^ = 19%
	Mixed	3	3	−0.11 (-0.30, 0.09), *P = *.280	4	
	Unmedicated	13	13	−0.24 (-0.32, -0.16), *P < *.001	79	
	Unreported	4	4	−0.10 (-0.25, 0.05), *P = *.206	44	
ApoA1, g/L	Medicated	0	0	NA	NA	Chi² = 2.02, df = 2 (*P* = .364), *I*^2^ = 1%
	Mixed	6	6	0.01 (-0.01, 0.04), *P = *.360	18	
	Unmedicated	14	14	−0.01 (-0.02, 0.00), *P = *.163	40	
	Unreported	8	8	−0.00 (-0.03, 0.02), *P = *.856	0	
ApoB, g/L	Medicated	0	0	NA	NA	Chi² = 7.91, df = 2 (*P* = .019), *I*^2^ = 75%
	Mixed	6	6	−0.01 (-0.05, 0.03), *P = *.729	67	
	Unmedicated	20	20	−0.05 (-0.06, -0.03), *P < *.001	78	
	Unreported	8	8	−0.01 (-0.04, 0.01), *P = *.338	0	
Lp(a), g/L	Medicated	1	1	−0.00 (-0.03, 0.03), *P = *.890	NA	Chi² = 0.14, df = 2 (*P* = .932), *I*^2^ = 0%
	Mixed	2	2	−0.00 (-0.03, 0.03), *P = *.967	30	
	Unmedicated	9	9	0.00 (0.00, 0.01), *P = *.021	0	
	Unreported	0	0	NA	NA	

Abbreviations: Apo, apolipoprotein; CI, confidence interval; DBP, diastolic blood pressure; df, degree of freedom; HDL-C, high-density lipoprotein cholesterol; IDL-C, intermediate-density lipoprotein cholesterol; LDL-C, low-density lipoprotein cholesterol; Lp, lipoprotein; NA, not applicable; SBP, systolic blood pressure; TC, total cholesterol; TG, triglycerides; VLDL-C, very low-density lipoprotein cholesterol.

#### Lipids—TG, TC, LDL-C, HDL-C

**Figure 2.**
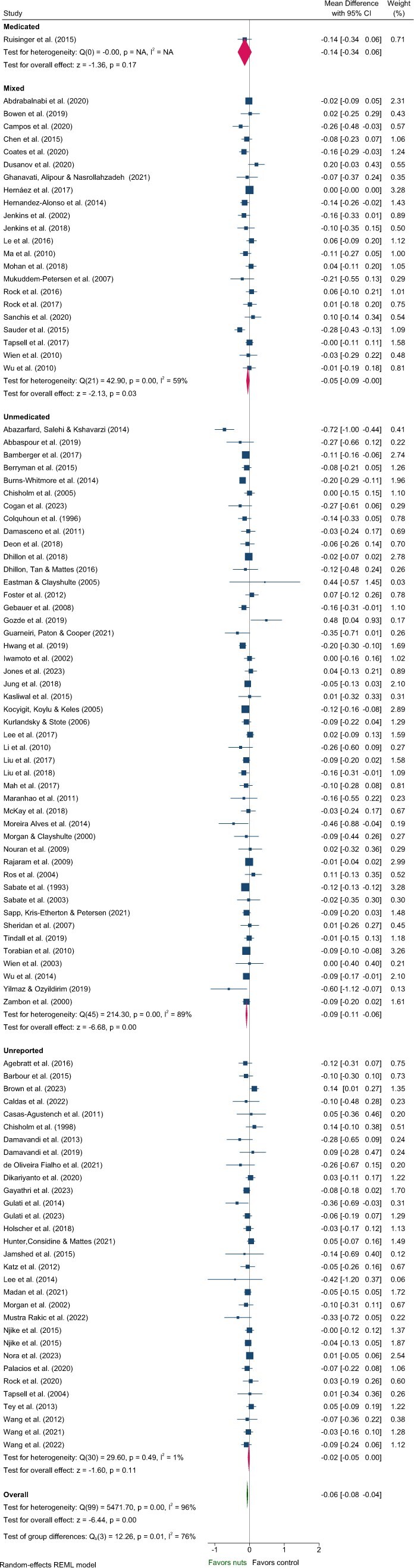
Forest plot of effects of nuts consumption on triglyceride (mmol/L), with sub-group analysis by medication status. Abbreviation: CI, confidence interval.

**Figure 3.**
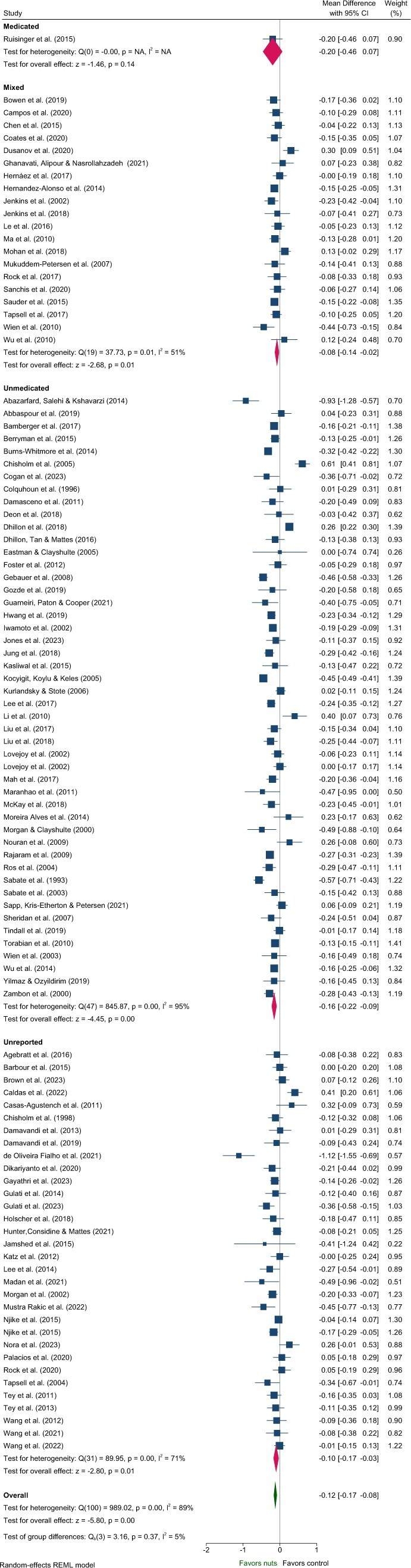
Forest plot of effects of nuts consumption on total cholesterol (mmol/L), with sub-group analysis by medication status. Abbreviation: CI, confidence interval.

**Figure 4.**
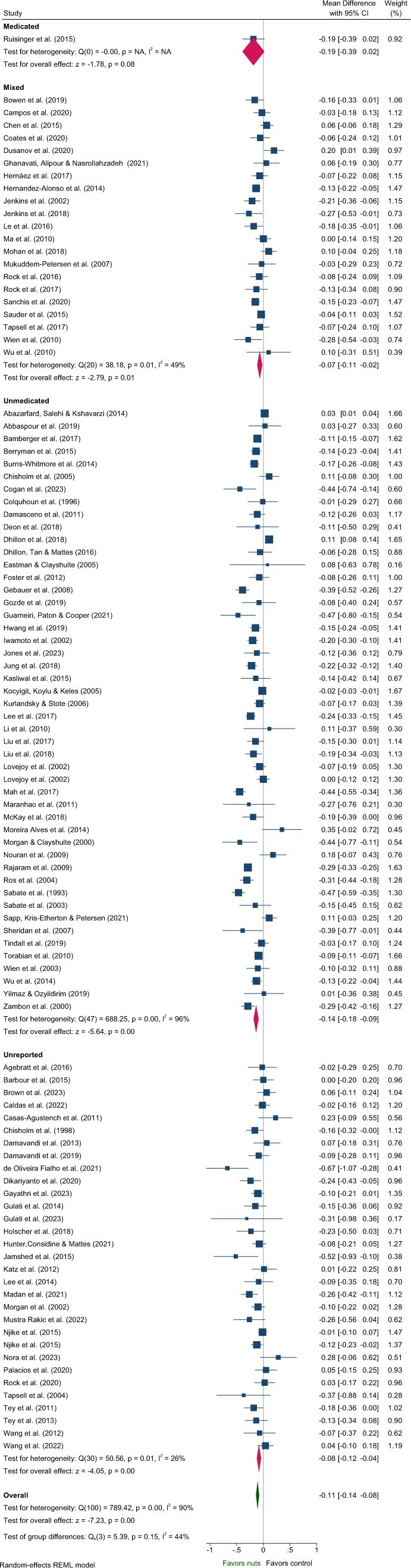
Forest plot of effects of nuts consumption on low-density lipoprotein-cholesterol (mmol/L), with sub-group analysis by medication status. Abbreviation: CI, confidence interval.

Five articles identified in the systematic review were excluded from the meta-analysis of lipids due to insufficient data^46^ and poor quality.^48–51^ These studies predominantly included Unmedicated participants,^46^^,^^48^^,^^50^^,^^51^ with 1 study classified as Unreported.^49^ One study reported a significant reduction in TC and increased HDL-C with nut consumption compared to control,^50^ while another reported significantly reduced LDL-C.^51^ Effects of nuts on TC, LDL-C, and HDL-C were evaluated in 101 strata from 99 interventions while TG were evaluated in 100 strata from 99 interventions. Nut consumption resulted in a significantly greater decrease in TG, TC, and LDL-C compared to the control overall (Table 2, Figures 2–4). There was no significant effect on HDL-C (Table 2, Figure S3). Evaluation of medication use identified a significant between-group difference for TG (Table 3), with significant effects of nuts observed in the Mixed and Unmedicated groups. For TC and LDL-C, despite a significant effect of nuts within the Unmedicated, Unreported, and Mixed subgroups, the test for subgroup differences indicated that medication use does not modify the effect (Table 3). The single study conducted in Medicated individuals did not reveal a significant effect of nuts compared to control for TG, TC, LDL-C, or HDL-C.

Subgroup analyses indicated significant differences related to health status for the effect of nuts on TG and HDL-C (Tables S7 and S10). Variation in the magnitude of effect on TG was also observed for nut type and nut dose, and for TC and LDL-C by nut type, although with moderate–high heterogeneity across most subgroups (Tables S7–S9). Larger reductions in LDL-C were observed for crossover study designs and interventions that did not implement concurrent energy restriction (Table S9). However, these findings should be interpreted with caution due to heterogeneity >80% for these subgroups.

#### Other Lipid Outcomes—Non–-HDL-C, VLDL-C, IDL-C, Apo B, Apo A1, and Lp(a)

**Figure 5.**
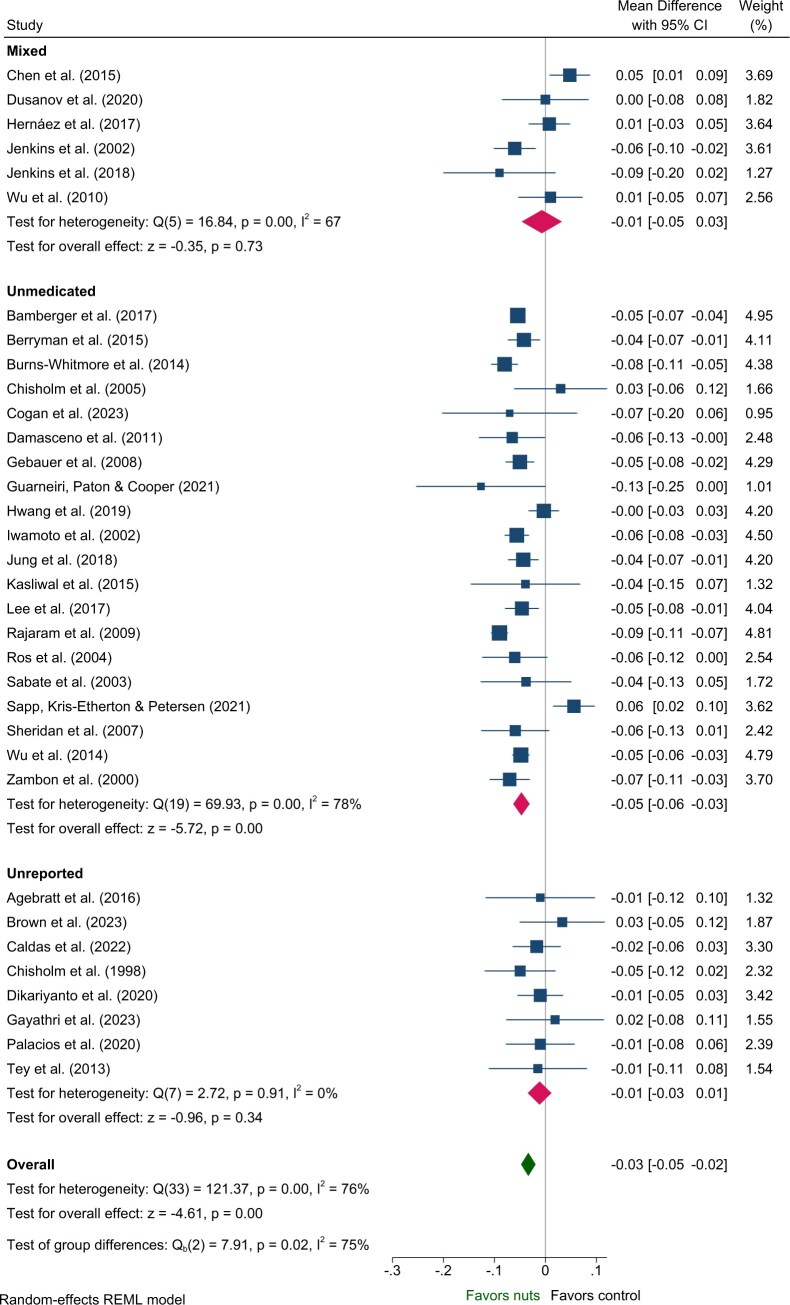
Forest plot of effects of nuts consumption on apolipoprotein B (g/L), with subgroup analysis by medication status. Abbreviation: CI, confidence interval.

Effects on VLDL-C, non–HDL-C, and ApoB were evaluated in 24, 21, and 34 strata (from the same number of interventions), respectively. Nut consumption resulted in a significantly greater decrease in VLDL-C, non-HDL-C, and ApoB compared to control overall (Table 2, Figures S4 and S5, Figure 5). Evaluation of medication use identified significant between-group differences for ApoB, with significant effects of nuts observed within the Unmedicated, but not the Mixed or Unreported medication use groups (Table 3, Figure 5). While significant benefits of nuts were observed for VLDL-C and non-HDL-C within the Unmedicated subgroup, the magnitude of effect did not differ among medication use subgroups (Table 3). The single study involving Medicated individuals reported a significant reduction in VLDL-C.

The meta-analysis showed that nut consumption did not result in significant mean differences in IDL-C and ApoA1 following nut consumption (Table 2, Figure S6). Significant reductions in Lp(a) were observed with nut consumption; however, this outcome was evaluated only in a small number of studies (*n* = 12) and the resulting magnitude of effect had little clinical importance (Table 2, Figure S7). One study^47^ reporting no effect of pistachios on Lp(a) was not included in the meta-analysis due to inability to convert values to common units (grams per liter). While nut consumption compared with control appears to lower Lp(a) in Unmedicated individuals, between-group analysis indicated that this effect does not differ from that in the other medication use subgroups. No significant effects were observed in any of the medication use subgroups for ApoA1 (Table 3). Medication use analyses were not conducted for IDL-C as it was reported in <10 studies.

When considering other subgroup analyses, significant differences in non-HDL-C were identified related to health status, concurrent energy restriction, and nut type (Table S12). A subgroup difference was observed for ApoA1 by energy restriction; however, the within-group changes were not significant and were likely influenced by the uneven distribution of studies across groups (Table S13). Larger reductions in ApoB were found in crossover compared to parallel studies, studies of shorter duration (<12 weeks), and by health status and nut type; however, these findings should be interpreted with caution due to high heterogeneity and an uneven distribution of studies by health status and nut type (Table S14). No subgroup differences were observed for VLDL-C or Lp(a) (Tables S11 and S15).

#### Heterogeneity

Overall, a high level of heterogeneity (≥75%) was observed for SBP and most key lipid outcomes (TG, TC, LDL-C, HDL-C, non–HDL-C, and ApoB). Moderate heterogeneity was observed for VLDL-C and IDL-C (Table 2), and low heterogeneity for the remaining outcomes, including DBP, ApoA1, and Lp(a) (Table 2). For the medication use analysis, moderate to high heterogeneity was observed between the trials within each of the groups for most outcomes (Table 3).

#### Publication Bias

Funnel plots with more than 10 studies in each were generated for BP and lipid outcomes (Figure S8). Egger’s test results did not detect funnel plot asymmetry for any of the BP or lipid measures included in this review (Table 2).

## DISCUSSION

This study is to our knowledge the first systematic review and meta-analysis to evaluate whether antihypertensive and antidyslipidemic medication use alters BP and lipid-lowering responses to nut consumption. Significant overall improvements with tree nut and peanut consumption were observed in many lipid parameters (TG, TC, LDL-C, VLDL-C, non-HDL-C, and ApoB but not HDL-C, SBP, or DBP), consistent with findings from previous reviews.^10^^,^^18^^,^^155^ This meta-analysis extends findings from previous analyses by incorporating additional studies, focusing exclusively on RCTs, and performing an analysis based on participant antihypertensive and antidyslipidemic medication use. A key finding of this analysis was that only 1 study reported specifically recruiting Medicated individuals, with all participants on statin therapy.^101^ This study reported that consuming 100 g/d of almonds significantly lowered non-HDL-C, VLDL-C, and IDL-C in people taking lipid-lowering medication, with no differences in TG, TC, LDL-C, HDL-C, or Lp(a) compared to control. Significant differences between medication groups for TG and ApoB were observed, suggesting that medication use may modulate the effect of nuts on these parameters; the greatest reductions were observed in Unmedicated participants. While significant effects of participants consuming nuts compared with controls were observed for other outcomes, most consistently for lipids (TC, TG, LDL-C), these results did not differ between medication use groups. Comparison of nut consumption with control did not reveal improved BP overall or by medication use.

Because many of the purported CVD benefits of nuts have overlapping mechanisms of action with antilipidemic and antihypertensive medications, it is important to know whether the effects attributed to medication may limit any additional effect attributed to nut consumption or whether there is a synergistic benefit from the combination. Unfortunately, the outcomes of this review do not substantiate any relationships because only 1 intervention was included that evaluated the effects of nut consumption on lipids in Medicated individuals. Furthermore, none of the interventions in the Mixed group separated the reporting of results for BP and lipid outcomes based on medication use. The length of medication usage was also not reported. Additionally, approximately one-third of the interventions that measured lipid outcomes, and more than half of those which measured BP outcomes, did not specify whether they included Medicated participants and were therefore classified as Unreported medication status.

This review includes data for a diverse range of population groups made up of study participants with pre-existing CVD risk factors for conditions such as overweight/obesity, metabolic syndrome, and diabetes as well as participants considered to be in good health. This diversity among the study participants added to the richness and depth of the findings but also posed a challenge in assessing the true effect size, because studies with populations with existing comorbidities may produce a larger effect than those focusing solely on healthy individuals. Indeed, in the present study some of the subgroup analyses detected differences by health status, but the interpretation of these findings is limited by the uneven distribution of studies across study participant conditions. Overall, these findings highlight a significant research gap, and future studies should evaluate whether medication use influences the lipid- and BP-lowering effects of nut consumption in different health conditions.

While few studies have examined the effects of consuming nuts on lipids and BP in participants not taking medication compared with participants who are taking medication, some knowledge can be gained from studies that have considered cardiovascular benefits of medications alone and in combination with dietary patterns that contain nuts, such as the Mediterranean diet (MedD). A review of diet–statin drug interactions highlighted that consumption of foods rich in some mono- and polyunsaturated fats may alter cytochrome P-450 activation and in turn alter the half-life of some statins.^156^ The combination of statins and omega-3 fatty acids changes drug pharmacodynamics, resulting in an improved lipid profile and greater cardioprotection.^156^ Further support for these beneficial effects comes from a study that assessed the independent and combined effects of statins and the MedD on CVD mortality risk in 1180 patients followed up for 7.9 years. A greater reduction in CVD mortality risk was observed in patients treated with this diet–drug combination compared to the risk in patients using statins alone or those with average to high adherence to MedD.^157^ This combined benefit of statins and MedD was postulated to be attributable to a reduction in low-grade inflammation rather than effects on blood cholesterol,^157^ given that lipid-lowering medication has been shown to have anti-inflammatory effects.^158^ Thus, assessment of low-grade inflammation using biomarkers such as C-reactive protein may be relevant for future interventions testing the effectiveness of lipid reduction combined with nut consumption in medicated and unmedicated patient populations.

In comparison, the Dietary Approaches to Stop Hypertension diet (another dietary pattern that contains nuts) was found to be more effective in lowering BP in participants who were unmedicated than in participants taking antihypertensive medication.^159^ This result was also observed in other studies of participants with healthy and low-sodium dietary patterns.^160^ These study results suggests that when medicated and unmedicated study populations are combined in the same analysis, the effectiveness of the dietary intervention may be blunted. It is important to note, however, that all populations consuming a healthy dietary pattern achieved a reduction in BP although it differed in magnitude depending on medication use.

A database of comprehensive food–drug interactions (FooDrugs) has recently been developed to ensure that food–drug interactions are listed in a central repository that is free to access, thus providing a valuable resource to researchers and clinicians.^161^ This database should be referenced in CVD management guidelines and continuing education sessions offered to practitioners in the management of diet and CV health. Furthermore, an update to reporting guidelines for RCTs in nutrition is currently underway^162^^,^^163^ which should lead to improvements in the quality of reporting in nutrition interventions. While these recommendations are an important step, future guidelines could encourage the clear reporting of medication when it is associated analyses of unmedicated vs medicated individuals or that this specific data be made available on request to enable evaluation of diet–drug interactions.

### Strengths and Limitations

This review has several noteworthy strengths. The comprehensive approach includes an examination of a wide range of lipid biomarkers., including SBP, DBP, TG, TC, LDL-C, HDL-C, VLDL-C, IDL-C, non-HDL-C, ApoA1, ApoB, and Lp(a), along with BP, and the findings are aligned with those of previous reviews of randomized and nonrandomized controlled trials.^10^^,^^18^^,^^155^ Studies were included in the meta-analysis if of sufficient quality, and rated as “positive” or “neutral.” While a moderate-to-high level of inconsistency was observed for the majority of outcomes, there was no evidence of publication bias.

This review included studies of participants who consumed a wide range of nut types and nut doses, which were incorporated into diets as a proportion of total energy intake or as a prespecified amount. Subgroup analyses were used to identify specific nut and dose effects for several outcomes; however, there was no consistency in the observed relationships. Variations were also evident in background diets (eg, habitual, energy restricted, Mediterranean, or specific-disease management), which may have introduced additional sources of variation. Given the recent interest in nuts for weight management,^43^^,^^44^ in this review we evaluated whether the inclusion of nuts in an energy-restricted diet resulted in differences in lipids or BP. Larger reductions in LDL-C and non-HDL-C were observed when nuts were not combined with energy restriction; however, these results should be interpreted with caution given the comparatively fewer studies involving energy restriction.

More specifically, the categorization of the articles in this review was based on the use of antihypertensive and antidyslipidemic medications by study participants. However, it is important to consider that antidyslipidemic medications, such as statins, also have a statistically significant and clinically meaningful effect on BP, albeit relatively small.^164^ This effect could not be captured in this review, emphasizing the need for future studies to explore the impact of combined medications on BP outcomes. Moreover, there were relatively few studies on some of the outcome variables, including VLDL-C, IDL-C, non-HDL-C, ApoA1, ApoB, and Lp(a), and in some medication use groups, which should be taken into consideration when interpreting the entire body of evidence.

## CONCLUSION

This review provides insight into whether or not medication use modulates the effects of nut consumption on BP and lipid outcomes. When assessed by medication status, significant differences in the effects of nuts on lipids were observed for TG and ApoB, with larger reductions observed in Mixed and Unmedicated participants for TG, and Unmedicated for ApoB. Nut consumption did not lead to greater reductions in SBP or DBP, with medication status not affecting these outcomes. However, the limited number of interventions involving medicated participants, coupled with inadequate reporting of medication status in one-third of the lipid studies and more than half of the BP studies, creates uncertainty regarding the efficacy of nut consumption in the presence of antihypertensive and lipid-lowering medications. To address these gaps in knowledge, future studies should improve reporting of medication use and consider evaluating or making data available on effects based on participants’ medication status, an approach offering the potential to uncover additional benefits of including nuts in the diet of medicated individuals. Health messaging to promote the consistent consumption of nuts as a component of CVD risk reduction strategies holds the promise of positively influencing the public perception of this food and reinforcing the importance on nut consumption in fostering heart-healthy lifestyle changes.

### Author Contributions

All authors conceived and designed the analysis. H.W. performed the literature search. H.W., A.M.C., S.C., and A.M.H. performed data screening. H.W. performed data extraction and quality assessment. A.M.H. completed the analysis. H.W. and A.M.H. synthesized the data. H.W. completed the initial first draft of the manuscript, with key revisions by A.M.H. All authors performed a critical review of the manuscript and have read and approved the final manuscript.

## Supplementary Material

nuaf033_Supplementary_Data

## Data Availability

Data will be made available on request.
