# Supplementary material for: Does Medication Status Impact the Effectiveness of Nuts in Altering Blood Pressure and Lipids? A Systematic Review and Meta-Analysis
Source: Nutr Rev. 2025 Apr 1;83(10):1843–60. doi: 10.1093/nutrit/nuaf033 (PMC12422015; doi:10.1093/nutrit/nuaf033)
Supplement: nuaf033_Supplementary_Data [file nuaf033_supplementary_data.zip › Supplementary Figure 3 HDL-C.pdf]

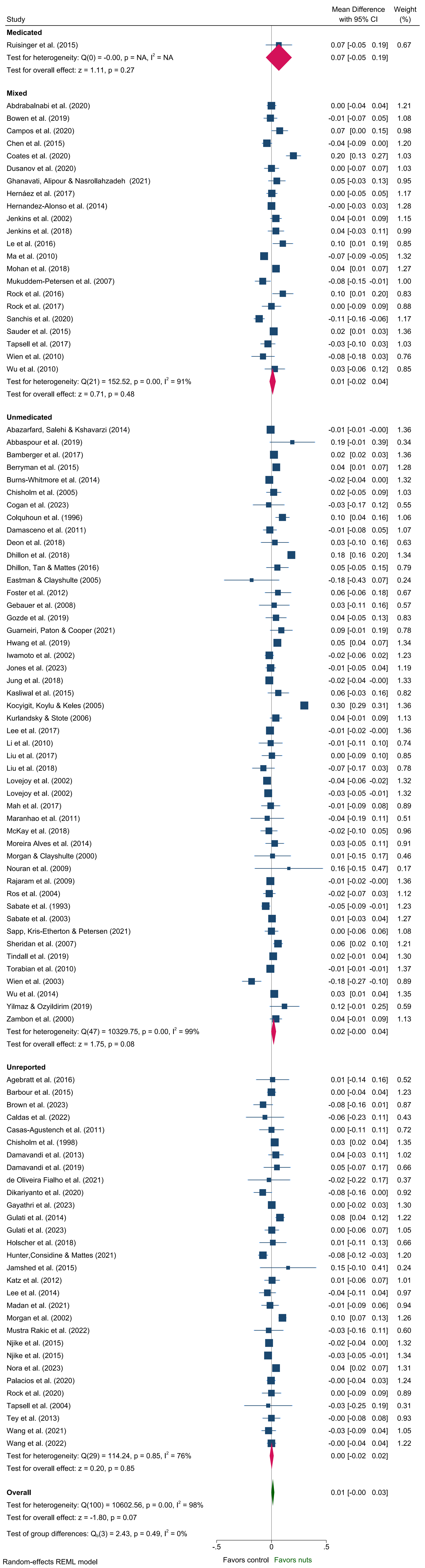

Study
Mean Difference with 95% CI
Weight (%)
Medicated
Ruisinger et al. (2015) 0.07 [-0.05 0.19] 0.67
Test for heterogeneity: Q(0) = -0.00, p = NA, I² = NA 0.07 [-0.05 0.19]
Test for overall effect: z = 1.11, p = 0.27
Mixed
Abdrabalnabi et al. (2020) 0.00 [-0.04 0.04] 1.21
Bowen et al. (2019) -0.01 [-0.07 0.05] 1.08
Campos et al. (2020) 0.07 [0.00 0.15] 0.98
Chen et al. (2015) -0.04 [-0.09 0.00] 1.20
Coates et al. (2020) 0.20 [0.13 0.27] 1.03
Dusanov et al. (2020) 0.00 [-0.07 0.07] 1.03
Ghanavati, Alipour & Nasrollahzadeh (2021) 0.05 [-0.03 0.13] 0.95
Hernáez et al. (2017) 0.00 [-0.05 0.05] 1.17
Hernandez-Alonso et al. (2014) -0.00 [-0.03 0.03] 1.28
Jenkins et al. (2002) 0.04 [-0.01 0.09] 1.15
Jenkins et al. (2018) 0.04 [-0.03 0.11] 0.99
Le et al. (2016) 0.10 [0.01 0.19] 0.85
Ma et al. (2010) -0.07 [-0.09 -0.05] 1.32
Mohan et al. (2018) 0.04 [0.01 0.07] 1.27
Mukuddem-Petersen et al. (2007) -0.08 [-0.15 -0.01] 1.00
Rock et al. (2016) 0.10 [0.01 0.20] 0.83
Rock et al. (2017) 0.00 [-0.09 0.09] 0.88
Sanchis et al. (2020) -0.11 [-0.16 -0.06] 1.17
Sauder et al. (2015) 0.02 [0.01 0.03] 1.36
Tapsell et al. (2017) -0.03 [-0.10 0.03] 1.03
Wien et al. (2010) -0.08 [-0.18 0.03] 0.76
Wu et al. (2010) 0.03 [-0.06 0.12] 0.85
Test for heterogeneity: Q(21) = 152.52, p = 0.00, I² = 91% 0.01 [-0.02 0.04]
Test for overall effect: z = 0.71, p = 0.48
Unmedicated
Abazarfard, Salehi & Kshavarzi (2014) -0.01 [-0.01 -0.00] 1.36
Abbaspour et al. (2019) 0.19 [-0.01 0.39] 0.34
Bamberger et al. (2017) 0.02 [0.02 0.03] 1.36
Berryman et al. (2015) 0.04 [0.01 0.07] 1.28
Burns-Whitmore et al. (2014) -0.02 [-0.04 0.00] 1.32
Chisholm et al. (2005) 0.02 [-0.05 0.09] 1.03
Cogan et al. (2023) -0.03 [-0.17 0.12] 0.55
Colquhoun et al. (1996) 0.10 [0.04 0.16] 1.06
Damasceno et al. (2011) -0.01 [-0.08 0.05] 1.07
Deon et al. (2018) 0.03 [-0.10 0.16] 0.63
Dhillon et al. (2018) 0.18 [0.16 0.20] 1.34
Dhillon, Tan & Mattes (2016) 0.05 [-0.05 0.15] 0.79
Eastman & Clayshulte (2005) -0.18 [-0.43 0.07] 0.24
Foster et al. (2012) 0.06 [-0.06 0.18] 0.67
Gebauer et al. (2008) 0.03 [-0.11 0.16] 0.57
Gozde et al. (2019) 0.04 [-0.05 0.13] 0.83
Guarneiri, Paton & Cooper (2021) 0.09 [-0.01 0.19] 0.78
Hwang et al. (2019) 0.05 [0.04 0.07] 1.34
Iwamoto et al. (2002) -0.02 [-0.06 0.02] 1.23
Jones et al. (2023) -0.01 [-0.05 0.04] 1.19
Jung et al. (2018) -0.02 [-0.04 -0.00] 1.33
Kasliwal et al. (2015) 0.06 [-0.03 0.16] 0.82
Kocyigit, Koylu & Keles (2005) 0.30 [0.29 0.31] 1.36
Kurlandsky & Stote (2006) 0.04 [-0.01 0.09] 1.13
Lee et al. (2017) -0.01 [-0.02 -0.00] 1.36
Li et al. (2010) -0.01 [-0.11 0.10] 0.74
Liu et al. (2017) 0.00 [-0.09 0.10] 0.85
Liu et al. (2018) -0.07 [-0.17 0.03] 0.78
Lovejoy et al. (2002) -0.04 [-0.06 -0.02] 1.32
Lovejoy et al. (2002) -0.03 [-0.05 -0.01] 1.32
Mah et al. (2017) -0.01 [-0.09 0.08] 0.89
Maranhao et al. (2011) -0.04 [-0.19 0.11] 0.51
McKay et al. (2018) -0.02 [-0.10 0.05] 0.96
Moreira Alves et al. (2014) 0.03 [-0.05 0.11] 0.91
Morgan & Clayshulte (2000) 0.01 [-0.15 0.17] 0.46
Nouran et al. (2009) 0.16 [-0.15 0.47] 0.17
Rajaram et al. (2009) -0.01 [-0.02 -0.00] 1.36
Ros et al. (2004) -0.02 [-0.07 0.03] 1.12
Sabate et al. (1993) -0.05 [-0.09 -0.01] 1.23
Sabate et al. (2003) 0.01 [-0.03 0.04] 1.27
Sapp, Kris-Etherton & Petersen (2021) 0.00 [-0.06 0.06] 1.08
Sheridan et al. (2007) 0.06 [0.02 0.10] 1.21
Tindall et al. (2019) 0.02 [-0.01 0.04] 1.30
Torabian et al. (2010) -0.01 [-0.01 -0.01] 1.37
Wien et al. (2003) -0.18 [-0.27 -0.10] 0.89
Wu et al. (2014) 0.03 [0.01 0.04] 1.35
Yilmaz & Ozyildirim (2019) 0.12 [-0.01 0.25] 0.59
Zambon et al. (2000) 0.04 [-0.01 0.09] 1.13
Test for heterogeneity: Q(47) = 10329.75, p = 0.00, I² = 99% 0.02 [-0.00 0.04]
Test for overall effect: z = 1.75, p = 0.08
Unreported
Agebratt et al. (2016) 0.01 [-0.14 0.16] 0.52
Barbour et al. (2015) 0.00 [-0.04 0.04] 1.23
Brown et al. (2023) -0.08 [-0.16 0.01] 0.87
Caldas et al. (2022) -0.06 [-0.23 0.11] 0.43
Casas-Agustench et al. (2011) 0.00 [-0.11 0.11] 0.72
Chisholm et al. (1998) 0.03 [0.02 0.04] 1.35
Damavandi et al. (2013) 0.04 [-0.03 0.11] 1.02
Damavandi et al. (2019) 0.05 [-0.07 0.17] 0.66
de Oliveira Fialho et al. (2021) -0.02 [-0.22 0.17] 0.37
Dikariyanto et al. (2020) -0.08 [-0.16 0.00] 0.92
Gayathri et al. (2023) 0.00 [-0.02 0.03] 1.30
Gulati et al. (2014) 0.08 [0.04 0.12] 1.22
Gulati et al. (2023) 0.00 [-0.06 0.07] 1.05
Holscher et al. (2018) 0.01 [-0.11 0.13] 0.66
Hunter,Considine & Mattes (2021) -0.08 [-0.12 -0.03] 1.20
Jamshed et al. (2015) 0.15 [-0.10 0.41] 0.24
Katz et al. (2012) 0.01 [-0.06 0.07] 1.01
Lee et al. (2014) -0.04 [-0.11 0.04] 0.97
Madan et al. (2021) -0.01 [-0.09 0.06] 0.94
Morgan et al. (2002) 0.10 [0.07 0.13] 1.26
Mustra Rakic et al. (2022) -0.03 [-0.16 0.11] 0.60
Njike et al. (2015) -0.02 [-0.04 0.00] 1.32
Njike et al. (2015) -0.03 [-0.05 -0.01] 1.34
Nora et al. (2023) 0.04 [0.02 0.07] 1.31
Palacios et al. (2020) -0.00 [-0.04 0.03] 1.24
Rock et al. (2020) 0.00 [-0.09 0.09] 0.89
Tapsell et al. (2004) -0.03 [-0.25 0.19] 0.31
Tey et al. (2013) -0.00 [-0.08 0.08] 0.93
Wang et al. (2021) -0.03 [-0.09 0.04] 1.05
Wang et al. (2022) -0.00 [-0.04 0.04] 1.22
Test for heterogeneity: Q(29) = 114.24, p = 0.85, I² = 76% 0.00 [-0.02 0.02]
Test for overall effect: z = 0.20, p = 0.85
Overall 0.01 [-0.00 0.03]
Test for heterogeneity: Q(100) = 10602.56, p = 0.00, I² = 98%
Test for overall effect: z = -1.80, p = 0.07
Test of group differences: Q_b(3) = 2.43, p = 0.49, I² = 0%
-.5 0 .5
Random-effects REML model
Favors control Favors nuts
