# Supplementary material for: Does Medication Status Impact the Effectiveness of Nuts in Altering Blood Pressure and Lipids? A Systematic Review and Meta-Analysis
Source: Nutr Rev. 2025 Apr 1;83(10):1843–60. doi: 10.1093/nutrit/nuaf033 (PMC12422015; doi:10.1093/nutrit/nuaf033)
Supplement: nuaf033_Supplementary_Data [file nuaf033_supplementary_data.zip › Supplementary Figures – Title and Legend_revised 18_11_24.docx]

**Supplementary Figures – Title and Legend**

**Supplementary Figure 1.** Forest plot of effects of nuts consumption on systolic blood pressure (mmHg), with subgroup analysis by medication status.

*Abbreviations*: CI, confidence interval

**Supplementary Figure 2.** Forest plot of effects of nuts consumption on diastolic blood pressure (mmHg), with subgroup analysis by medication status.

*Abbreviations*: CI, confidence interval

**Supplementary Figure 3.** Forest plot of effects of nuts consumption on high-density lipoprotein-cholesterol (mmol/L), with subgroup analysis by medication status.

*Abbreviations*: CI, confidence interval

**Supplementary Figure 4.** Forest plot of effects of nuts consumption on very-low-density lipoprotein-cholesterol (mmol/L), with subgroup analysis by medication status.

*Abbreviations*: CI, confidence interval

**Supplementary Figure 5.** Forest plot of effects of nuts consumption on non-high-density lipoprotein-cholesterol (mmol/L), with subgroup analysis by medication status.

*Abbreviations*: CI, confidence interval

**Supplementary Figure 6.** Forest plot of effects of nuts consumption on apolipoprotein A1 (g/L), with subgroup analysis by medication status.

*Abbreviations*: CI, confidence interval

**Supplementary Figure 7.** Forest plot of effects of nuts consumption on lipoprotein (a) (g/L), with subgroup analysis by medication status.

*Abbreviations*: CI, confidence interval

**Supplementary Figure 8.** Funnel plots of blood pressure and lipid outcomes evaluated in this review.

Abbreviations: Apo, apolipoprotein; DBP, diastolic blood pressure; HDL-C, high-density lipoprotein - cholesterol; LDL-C, low-density lipoprotein - cholesterol; Lp, lipoprotein; MD, mean difference; SBP, systolic blood pressure; TC, total cholesterol; TG, triglycerides; VLDL-C, very low-density lipoprotein – cholesterol
