# Supplementary material for: Does Medication Status Impact the Effectiveness of Nuts in Altering Blood Pressure and Lipids? A Systematic Review and Meta-Analysis
Source: Nutr Rev. 2025 Apr 1;83(10):1843–60. doi: 10.1093/nutrit/nuaf033 (PMC12422015; doi:10.1093/nutrit/nuaf033)
Supplement: nuaf033_Supplementary_Data [file nuaf033_supplementary_data.zip › Supplementary Tables 1-15_final version.docx]

# **Supplementary Table 1.** PRISMA checklist

| **Section and Topic** | **Item #** | **Checklist item** | **Location where item is reported** |
| --- | --- | --- | --- |
| **TITLE** | | |  |
| Title | 1 | Identify the report as a systematic review. | Page 1 |
| **ABSTRACT** | | |  |
| Abstract | 2 | See the PRISMA 2020 for Abstracts checklist. | Page 2-3 |
| **INTRODUCTION** | | |  |
| Rationale | 3 | Describe the rationale for the review in the context of existing knowledge. | Page 3-6 |
| Objectives | 4 | Provide an explicit statement of the objective(s) or question(s) the review addresses. | Page 6 |
| **METHODS** | | |  |
| Eligibility criteria | 5 | Specify the inclusion and exclusion criteria for the review and how studies were grouped for the syntheses. | Page 7, Table 1, Supplementary table 2 |
| Information sources | 6 | Specify all databases, registers, websites, organisations, reference lists and other sources searched or consulted to identify studies. Specify the date when each source was last searched or consulted. | Page 6-7 |
| Search strategy | 7 | Present the full search strategies for all databases, registers and websites, including any filters and limits used. | Supplementary table 2 |
| Selection process | 8 | Specify the methods used to decide whether a study met the inclusion criteria of the review, including how many reviewers screened each record and each report retrieved, whether they worked independently, and if applicable, details of automation tools used in the process. | Page 6-7, Figure 1 |
| Data collection process | 9 | Specify the methods used to collect data from reports, including how many reviewers collected data from each report, whether they worked independently, any processes for obtaining or confirming data from study investigators, and if applicable, details of automation tools used in the process. | Page 8-9 |
| Data items | 10a | List and define all outcomes for which data were sought. Specify whether all results that were compatible with each outcome domain in each study were sought (e.g. for all measures, time points, analyses), and if not, the methods used to decide which results to collect. | Page 8-11 |
|  | 10b | List and define all other variables for which data were sought (e.g. participant and intervention characteristics, funding sources). Describe any assumptions made about any missing or unclear information. | Page 8-11 |
| Study risk of bias assessment | 11 | Specify the methods used to assess risk of bias in the included studies, including details of the tool(s) used, how many reviewers assessed each study and whether they worked independently, and if applicable, details of automation tools used in the process. | Page 8-9 |
| Effect measures | 12 | Specify for each outcome the effect measure(s) (e.g. risk ratio, mean difference) used in the synthesis or presentation of results. | Page 9-11 |
| Synthesis methods | 13a | Describe the processes used to decide which studies were eligible for each synthesis (e.g. tabulating the study intervention characteristics and comparing against the planned groups for each synthesis (item #5)). | Page 9-11 |
|  | 13b | Describe any methods required to prepare the data for presentation or synthesis, such as handling of missing summary statistics, or data conversions. | Page 9-11 |
|  | 13c | Describe any methods used to tabulate or visually display results of individual studies and syntheses. | Page 9-11 |
|  | 13d | Describe any methods used to synthesize results and provide a rationale for the choice(s). If meta-analysis was performed, describe the model(s), method(s) to identify the presence and extent of statistical heterogeneity, and software package(s) used. | Page 11-12 |
|  | 13e | Describe any methods used to explore possible causes of heterogeneity among study results (e.g. subgroup analysis, meta-regression). | Page 11-12 |
|  | 13f | Describe any sensitivity analyses conducted to assess robustness of the synthesized results. | N/A |
| Reporting bias assessment | 14 | Describe any methods used to assess risk of bias due to missing results in a synthesis (arising from reporting biases). | Page 8-11 |
| Certainty assessment | 15 | Describe any methods used to assess certainty (or confidence) in the body of evidence for an outcome. | Page 9 |
| **RESULTS** | | |  |
| Study selection | 16a | Describe the results of the search and selection process, from the number of records identified in the search to the number of studies included in the review, ideally using a flow diagram. | Page 12-14, Figure 1 |
|  | 16b | Cite studies that might appear to meet the inclusion criteria, but which were excluded, and explain why they were excluded. | Page 12-14, Figure 1 |
| Study characteristics | 17 | Cite each included study and present its characteristics. | Page 12-14, Supplementary Table 3 |
| Risk of bias in studies | 18 | Present assessments of risk of bias for each included study. | Page 15, Supplementary Table 3 |
| Results of individual studies | 19 | For all outcomes, present, for each study: (a) summary statistics for each group (where appropriate) and (b) an effect estimate and its precision (e.g. confidence/credible interval), ideally using structured tables or plots. | Table 2-3, Figure 2-5, Supplementary Figure 1-7 |
| Results of syntheses | 20a | For each synthesis, briefly summarise the characteristics and risk of bias among contributing studies. | Page 15-18 |
|  | 20b | Present results of all statistical syntheses conducted. If meta-analysis was done, present for each the summary estimate and its precision (e.g. confidence/credible interval) and measures of statistical heterogeneity. If comparing groups, describe the direction of the effect. | Page 15-18, Table 2-3, Supplementary Table 5-15 |
|  | 20c | Present results of all investigations of possible causes of heterogeneity among study results. | Page 18 |
|  | 20d | Present results of all sensitivity analyses conducted to assess the robustness of the synthesized results. | N/A |
| Reporting biases | 21 | Present assessments of risk of bias due to missing results (arising from reporting biases) for each synthesis assessed. | Page 13-14, Supplementary Table 3-4, Supplementary Figure 8 |
| Certainty of evidence | 22 | Present assessments of certainty (or confidence) in the body of evidence for each outcome assessed. | Page 15, Supplementary Table 4 |
| **DISCUSSION** | | |  |
| Discussion | 23a | Provide a general interpretation of the results in the context of other evidence. | Page 19-22 |
|  | 23b | Discuss any limitations of the evidence included in the review. | Page 22-23 |
|  | 23c | Discuss any limitations of the review processes used. | Page 22-23 |
|  | 23d | Discuss implications of the results for practice, policy, and future research. | Page 19-23 |
| **OTHER INFORMATION** | | |  |
| Registration and protocol | 24a | Provide registration information for the review, including register name and registration number, or state that the review was not registered. | Page 3 |
|  | 24b | Indicate where the review protocol can be accessed, or state that a protocol was not prepared. | N/A |
|  | 24c | Describe and explain any amendments to information provided at registration or in the protocol. | N/A |
| Support | 25 | Describe sources of financial or non-financial support for the review, and the role of the funders or sponsors in the review. | Page 24-26 |
| Competing interests | 26 | Declare any competing interests of review authors. | Page 25-26 |
| Availability of data, code and other materials | 27 | Report which of the following are publicly available and where they can be found: template data collection forms; data extracted from included studies; data used for all analyses; analytic code; any other materials used in the review. | Page 26 |

*From:*  Page MJ, McKenzie JE, Bossuyt PM, Boutron I, Hoffmann TC, Mulrow CD, et al. The PRISMA 2020 statement: an updated guideline for reporting systematic reviews. BMJ 2021;372:n71. doi: 10.1136/bmj.n71

For more information, visit: <http://www.prisma-statement.org/>

# **Supplementary Table 2.** Combined search terms and strategies for MEDLINE, EMBASE, Web of Science and Scopus

| **Search terms:** Three groups of subject headings and keywords related to 1. nuts, 2. lipids and 3. BP were combined using Boolean operators to identify clinical trials investigating changes in blood lipid and BP parameters during nut intervention | |
| --- | --- |
| **Keyword Group** |  |
| 1 | “nut”, “nuts”, “almond?”, “walnut?”, “hickory”, “juglans”, “peanut?”, “goober?”, “pistachio?”, “pecan?”, “macadamia?”, “cashew?”, “hazelnut?”, “filbert?”, "brazil nut", "pine nut", "tree nuts", "mixed nuts", “groundnut”, “arachis?”, “corylus?”, “carya?”, "prunus dulcis", “pistacia?”, “anacardium?” or “prunus?” |
| 2 | “dyslipid?emia”, “hyperlipid?emia”, “hypercholesterolemia”, “lipoprotein*”, “cholesterol*”, “triglyceride*”, “apolipoprotein B”, “ApoB”, “apolipoprotein A1”, “Apo?A1”, “low?density lipoprotein?”, “LDL?”, “high?density lipoprotein?”, “HDL?”, “very?low?density lipoprotein?”, “VLDL?”, “Intermediate?density lipoprotein?” or “IDL?” |
| 3 | "blood pressure", “hypertension”, “high blood pressure”, “systolic blood pressure” or “diastolic blood pressure” |
|  |  |
|  | “Keyword group 1 AND Keyword group 2” OR “Keyword group 1 AND Keyword group 3” |

| **Search Strategy for MEDLINE and EMBASE** | |
| --- | --- |
| **No.** | **Search** |
| 1 | SH: Nuts |
| 2 | nut OR nuts OR almond? OR walnut? OR hickory OR juglans OR peanut? OR goober? OR pistachio? OR pecan? OR macadamia? OR cashew? OR hazelnut? OR fibert? OR "brazil nut" OR "pine nut" OR "tree nuts" OR "mixed nuts" OR groundnut OR arachis? OR Corylus? OR carya? OR "prunus dulcis" OR Pistacia? OR anacardium? OR prunus? |
| 3 | SH: Lipids |
| 4 | SH: cholesterol |
| 5 | SH: exp/lipoprotein |
| 6 | dyslipid?emia OR hyperlipid?emia OR hypercholesterolemia OR lipoprotein* OR cholesterol* OR triglyceride* OR “apolipoprotein B” OR ApoB OR “apolipoprotein A1” OR Apo?A1 OR “low?density lipoprotein?” OR LDL? OR “high?density lipoprotein?” OR HDL? OR “very?low?density lipoprotein?” OR VLDL? OR “Intermediate?density lipoprotein?” OR IDL? |
| 7 | SH: blood pressure |
| 8 | "blood pressure" OR hypertension OR “high blood pressure” OR “systolic blood pressure” OR “diastolic blood pressure” |
| 9 | 1 OR 2 |
| 10 | 3 OR 4 OR 5 OR 6 |
| 11 | 7 OR 8 |
| 12 | 9 AND 10 |
| 13 | 9 AND 11 |
| 14 | 12 OR 13 |
| 15 | Limit 14 to Humans |
| 16 | Limit 15 to English Language |
|  | |
| **Search Strategy for Web of Science and Scopus** | |
| ((nut OR nuts OR almond? OR walnut? OR hickory OR juglans OR peanut? OR goober? OR pistachio? OR pecan? OR macadamia? OR cashew? OR hazelnut? OR fibert? OR "brazil nut" OR "pine nut" OR "tree nuts" OR "mixed nuts" OR groundnut OR arachis? OR Corylus? OR carya? OR "prunus dulcis" OR Pistacia? OR anacardium? OR prunus?) AND (dyslipid?emia OR hyperlipid?emia OR hypercholesterolemia OR lipoprotein* OR cholesterol* OR triglyceride* OR “apolipoprotein B” OR ApoB OR “apolipoprotein A1” OR Apo?A1 OR “low?density lipoprotein?” OR LDL? OR “high?density lipoprotein?” OR HDL? OR “very?low?density lipoprotein?” OR VLDL? OR “Intermediate?density lipoprotein?” OR IDL?))  **OR**  ((nut OR nuts OR almond? OR walnut? OR hickory OR juglans OR peanut? OR goober? OR pistachio? OR pecan? OR macadamia? OR cashew? OR hazelnut? OR fibert? OR "brazil nut" OR "pine nut" OR "tree nuts" OR "mixed nuts" OR groundnut OR arachis? OR Corylus? OR carya? OR "prunus dulcis" OR Pistacia? OR anacardium? OR prunus?) AND ("blood pressure" OR hypertension OR “high blood pressure” OR “systolic blood pressure” OR “diastolic blood pressure”)) | |

# **Supplementary Table 3:** Characteristics and outcomes of the included studies in the review (n=115)

| **Author (year); Country** | **Study Design,  Duration** | **No. analysed (no. female)** | **Mean Age ± SD (years)**  **Mean BMI ± SD (kg/m^2^)**  **Health Status** | **Use of Antihypertensive medications (Type of medication: % using)** | **Use of Antihyperlipidemic medications (Type of medication: % using)** | **Control** | **Intervention** | **Outcomes measured (IG VS CG, between-group variations)** | **QA** |
| --- | --- | --- | --- | --- | --- | --- | --- | --- | --- |
| Abazarfard, Salehi & Kshavarzi (2014)^79^; Iran | P, 3 months | IG: 50 (50); CG: 50 (50) | IG: 42.36 ± 7.30, CG: 42.94 ± 6.82  IG: 29.91 ± 1.20 CG: 29.37 ± 1.73   Overweight and obese premenopausal women with BMI ≥25 kg/m^2^ who had no exercise or only light physical activities | Unreported | Unmedicated | Low-calorie diet - 1000 kcal were reduced from the total calculated energy requirement (approximately 54% of energy from carbohydrates, 16% from proteins, and 30% from fat). Compensatory servings of meat and fat (e.g. sunflower oil and corn oil) were used to replace almonds in IG. | Low-calorie diet same as CG with 50g/d raw almond in the form of two snacks (25g per snack) included. | SBP ↑, DBP↓, TG ↓, TC ↓, LDL-C ↑, HDL-C ↓ | (+) |
| Abbaspour et al. (2019)^76^; USA | P, 8 weeks | IG: 24 (10); CG: 24 (9) | IG: 30.4 ± 10.2, CG: 29.1 ± 9.3   IG: 30.9 ± 2.8 CG: 31.6 ± 3.1   Overweight and obese adults with a BMI of ≥ 27 kg/m^2^ | Unmedicated | Unmedicated | Usual diet with 69g/d (250kcal) unsalted pretzels as an isocaloric snack. | Usual diet with 42.5g/d (250kcal) roasted and slightly salted mixed nuts (containing -almonds, cashews, hazelnuts, pecans, Brazil nuts, macadamias, pistachios, walnuts, and peanuts) as an isocaloric snack. | SBP →, DBP →, TG →, TC →, LDL-C →, HDL-C → | (∅) |
| Abdrabalnabi et al. (2020)^61^; Spain & USA | P, 2 years | IG: 319; CG: 306 (420-only report the total female number for the whole trial) | All participants: 69.1 ± 3.6   IG: 27.1 ± 2.55, CG: 27.5 ± 2.55   Healthy elderly individuals | Mixed (All types: 46.7%) | Mixed (Statins: 31.7%; Other lipid-lowering drugs: 2.7%) | Habitual diet. Abstain from eating walnuts and other tree nuts. | Habitual diet with walnuts equivalent to 15% of daily energy needs (30, 45, or 60g/d). | SBP →, DBP →, TG →, HDL-C → | (∅) |
| Agebratt et al. (2016)^63^; Canada | P, 2 months | IG: 15 (4); CG: 15 (8) | All participants: 23.5 ± 3.7   IG: 22.54 ± 2.26, CG: 22.15 ± 1.61   Healthy participants | Unreported | Unreported | Usual diet with 7kcal/kg of body weight per day of fruit (any fresh, unprocessed fruits - bananas, apples citrus fruits, pears, melons, grapes mangos, kiwis, or persimmons with less than 1% each from pineapples or plums etc.) between regular meals. | Usual diet with 7kcal/kg of body weight per day of nuts (any nuts - cashews (47.4%), peanuts (14.1%), walnuts (8.1%), almonds (8.0%), pistachios (7.8%), hazelnuts (5.8%), Brazil nuts (3.5%), unspecified “nut mixes” (3.4%), macadamias or pecans (<1%)) between regular meals. | SBP →, DBP →, TG →, TC →, LDL-C →, HDL-C →, Apo A1 →, Apo B →, Lp(a) ↓ | (∅) |
| Bamberger et al. (2017)^56^; Germany | CR, 8 weeks per arm | 204 (134/194-completed) | All participants: 63 ± 0.54   All participants: 25.4 ± 0.29   Healthy, non-smoking, men and postmenopausal women, LDL-C <190 mg/dL, TG <350 mg/dL, and BMI <35 kg/m^2^ | Unreported | Unmedicated | Nut-free Western-type diet (approximately 50% of energy from carbohydrates, 15% from protein, and 35% from fat [15% saturated fat]). *Specified this was consumed during run-in and washout but not specifically during the control period | Walnut-enriched diet (43g/d shelled walnuts) | TG ↓: TC ↓; LDL-C ↓; HDL-C →; VLDL-C ↓; non-HDL-C ↓; Apo B ↓; Lp(a) → | (∅) |
| Barbour et al. (2015)^78^; Australia | CR, 12 weeks per arm | 61(32) | All participants: 65 ± 7   All participants: 31 ± 4   Healthy overweight males or post-menopausal females with self-reported stable weight and BMI ≥ 25kg/m^2^ | Unreported | Unreported | Habitual diet (without nuts) | Habitual diet with 15%–20% of energy from roasted, unsalted high oleic (Australian Middleton cultivar) peanuts with skins (84g/d for males, 56g/d females, 6 days per week) | TG →, TC →, LDL-C →, HDL-C → | (+) |
| Bashan & Bakman (2018)^48^; Turkey^#^ | P, 3 months | IG: 73 (48); CG: 72 (48) | IG: 41.0 ± 10.84 CG: 40.7 ± 9.59   IG: 30.9 ± 2.55 CG: 30.1 ± 2.91   Adult with dyslipidaemia | Unmedicated | Unmedicated | Individualised diet consistent with AHA Dietary Guidelines | Individualised diet consistent with AHA Dietary Guidelines with 40–50g/day (one handful) Maras 18 cultivar walnuts (Energy from walnuts deducted from daily calorie intake). | TG (NR), TC (NR), LDL-C (NR), HDL-C (NR), VLDL-C (NR) | (-) |
| Berryman et al. (2015)^147^; USA | CR, 6 weeks per arm | 48 (26) | All participants: 49.9± 9.4   All participants: 26.2 ± 2.8   Men and women with a BMI of 20-35 kg/m^2^, and with elevated LDL-C (≥121-190 for females and 128-194 mg/dL for males), free of any chronic illness, and did not use tobacco | Unreported | Unmedicated | Cholesterol-lowering diet (approximately 58% of energy from carbohydrate, 15% from protein, 26% from total fat) with 106 g banana muffin and 2.7 g butter (273 kcal/d) | Cholesterol-lowering diet (approximately 51% of energy from carbohydrate, 16% from protein, 32% from total fat) with 42.5g/d unsalted, whole, natural almonds with skins (253 kcal/d) | TG →, TC ↓, LDL-C ↓, HDL-C ↑, VLDL-C ↓, IDL-C →, non-HDL-C ↓, Apo A1 →, Apo B ↓, Lp(a) → | (+) |
| Bowen et al. (2019)^145^; Australia | P, 8 weeks | IG: 39 (14); CG: 35 (17) | IG: 60.8 ± 6.6, CG: 60.6 ± 8.8   IG: 34.4 ± 6.2, CG: 33.2 ± 4.9   Overweight or obese (BMI ≥ 27 kg/m^2^), with an elevated WC (>88 cm females, >102 cm males), and elevated fasting plasma glucose (≥5.6 - <7.0 mmol/L) or T2D (confirmed by evidence of previous diagnosis) and not taking any diabetes medication and/or HbA1c >10%, and weight stable (i.e. <3 kg weight loss/gain in the past 2 months) | Unreported | Mixed (All types: 26%, 8/39 in IG and 12/37 in CG) | Usual *ad libitum* diet with 72 g/d (isocaloric snack as IG) sweet, nut- and seed-free commercial biscuits (The Original Scotch Finger, Arnott's Biscuits) as morning and afternoon snacks (36g each time) | Usual *ad libitum* diet with 56g/d of raw almonds as morning and afternoon snacks (28g each time) | TG →, TC →, LDL-C →, HDL-C → | (+) |
| Brown et al. (2023)^69^; New Zealand | P, 1 year | IG: 57 (52/71-randomised); CG: 52 (49/65-randomised) | IG: 35.3 ± 13.6, CG: 35.9 ± 13.4   IG: 23.9 ± 2.9, CG: 23.4 ± 3.2   Healthy men and women with a BMI of 18.5–29.9 kg/m^2^, and those who reported typically consuming 1500 kJ of discretionary foods each day | Unreported | Unreported | Habitual diet and replace their regular snacks to provided isocaloric snacks - biscuits (a mix of sweet cookies and savoury crackers) equivalent to the highest of either 10% total energy requirement (TER) or 42.5g almonds (1030kJ) | Habitual diet and replace their regular snacks to provided isocaloric snacks - roasted whole almonds equivalent to the highest of either 10% total energy requirement (TER) or 42.5g almonds (1030kJ) | TG →, TC →, LDL-C →, HDL-C →, Apo A1 →, Apo B → | (∅) |
| Burns-Whitmore et al. (2014)^65^; USA | CR, 8 weeks per arm | 20 (16) | All participants: 38 ± 3   All participants: 23 ± 1   Healthy free-living men and women who are lacto-ovo-vegetarians and have normal TC and TG levels | Unreported | Unmedicated | Habitual diet (lacto-ovo- vegetarian diet) + standard egg (6x/week) | Habitual diet (lacto-ovo- vegetarian diet) + 28.4g walnuts (6x/week) | TG ↓, TC ↓, LDL-C →, HDL-C →, Apo A1 →, Apo B ↓ | (∅) |
| Caldas et al. (2022)^129^; Brazil | P, 8 weeks | IG: 14 (14); CG: 15 (15) | IG: 31.2 ± 10.10, CG: 31.6 ± 8.13   IG: 33.8 ± 4.49, CG: 33.0 ± 3.87   Free-living conditions with women at cardiometabolic risk | Unreported | Unreported | Energy-restricted diet (–500kcal/day from the estimated energy requirement) without nuts, including two tablespoons (twice a day; at lunch and dinner) of a salad dressing based on soya oil and lemon (2:1 ratio) | Energy-restricted diet (–500 kcal/day from the estimated energy requirement), including 45g/d of nuts (15g of Brazil nuts + 30g of cashew nuts) | SBP →, DBP →, TG →, TC →, LDL-C →, HDL-C →, VLDL-C →, non-HDL-C →, Apo A1 →, Apo B → | (+) |
| Campos et al. (2020)^133^; Brazil | P, 12 weeks | IG: 68 (13); CG: 67 (18) | IG: 59.59 ± 8.40, CG: 60.40 ± 8.54   IG: 29.78 ± 5.60, CG: 29.06 ± 3.98   Patients diagnosed with stable CAD more than 60 days before the trial | Unreported | Mixed (Simvastatin: 80.7% in CG, 62.7% in IG; Rosuvastatin: 11.3% in CG, 18.6% in IG; Atorvastatin: 8.1% in CG, 18.6% in IG) | Received an individualised dietary prescription based on specific energy requirements. The distribution of macronutrients (carbohydrates, proteins, total fats, and fatty acids) in the CG diet was determined according to the Brazilian nutritional guidelines | Received an individualised dietary prescription based on specific energy requirements (same as CG) with 30g/d raw pecan nuts and advised not to consume olive oil | TG →, TC →, LDL-C →, HDL-C →, non-HDL-C ↓ | (+) |
| Casas-Agustench et al. (2011)^142^; Spain | P, 12 weeks | IG: 25 (10); CG: 25 (12) | IG: 52.9 ± 8.4, CG: 50.6 ± 8.4   IG: 31.6 ± 2.8, CG: 30.0 ± 3.3   Outpatient with metabolic syndrome | Unreported | Unreported | Receive recommendations of having a prudent diet (qualitative recommendations according to the American Heart Association dietary guidelines) with no nut or peanut intake | Receive the same dietary recommendations as CG with 30g/d of raw unpeeled nuts (15g walnuts, 7.5g almonds and 7.5g hazelnuts), with no specific instructions on food replacement | SBP →, DBP →, TG →, TC →, LDL-C →, HDL-C → | (∅) |
| Chen et al. (2015)^132^; USA | CR, 6 weeks per arm | 45 (27) | All participants: 61.8 ± 8.6   All participants: 30.2 ± 5.1   Patients with angiographically proven CAD from the cardiology practice at the Boston Medical Center | Mixed (vasodilators: 87%, beta-blockers: 84%, angiotensin-converting enzyme inhibitors: 38%, diuretics: 33%, and calcium channel blockers: 22%) | Mixed (statins: 96%) | A NCEP Step 1 diet without nuts | A NCEP Step 1 diet with 85 g/d raw whole almonds | SBP →, DBP →, TG →, TC →, LDL-C →, HDL-C →, Apo A1 →, Apo B → | (+) |
| Chisholm et al. (1998)^108^; New Zealand | CR, 4 weeks per arm | 21 (0) | All participants:45 ± 6.8   All participants: 28.4 ± 4.3   Moderately hyperlipidaemic men | Unreported | Unreported | Low-fat diets (with 30% energy from fat), individually prescribed, based on estimated energy requirements, and reported intake during the baseline. | Similar diet as CG with walnuts (on average, 78g walnuts/ day) contributed to 20% of the total energy. | TG →, TC →, LDL-C →, HDL-C →, Apo A1 →, Apo B ↓ | (∅) |
| Chisholm et al. (2005)^104^; New Zealand | CR, 6 weeks per arm | 28 (23) | All participants: 48.3 ± 10.3   All participants: 26.9 ± 3.2   Participants with moderately raised levels of TC and LDL-C | Unreported | Unmedicated | Low-fat diet (30-33% total energy from fats) with one serving (58g) of a cereal containing canola oil | Low-fat diet (same as CG) with 30g/d of nuts that are raw or roasted at a low heat without the addition of any fat or oil (participants were free to choose their preferred variety of nuts, including almonds, Brazil nuts, cashew nuts, hazelnuts, macadamia nuts, peanuts, pecan nuts, pistachio nuts, and walnuts) | SBP →, DBP →, TG →, TC →, LDL-C →, HDL-C →, Apo A1 →, Apo B → | (∅) |
| Choudhury, Clark & Griffiths (2014)^46^; UK^#^ | P, 4 weeks | IG – Healthy middle-aged: 15 (0); IG – Healthy young: 15 (0) IG- Young at risk: 15 (0); CG: 9 (0) | IG – Healthy middle-aged: 56.07 ± 5.77, IG – Healthy young: 22.14 ± 2.88, IG- Young at risk: 27.27 ± 5.04, CG:23.00 ± 5.87  IG – Healthy middle-aged: 25.37 ± 4.041, IG – Healthy young: 19.99 ± 1.595, IG- Young at risk: 26.34 ± 4.321, CG:22.20 ± 2.260   Males were recruited and grouped by age and health status. Group 1 subjects; ‘healthy middle-aged’ men; Group 2 subjects; ‘ healthy young ’ men; Group 3 subjects; ‘ young, at risk ’ for CVD men | Unreported | Unmedicated | Habitual diet for the 5 participants each from 3 groups | Habitual diet and substitute any daily snack for two bags of almonds (50g/d) for the 15 participants each from 3 groups | IG – Healthy middle-aged: SBP (NR), DBP (NR), TG (NR), TC (NR), LDL-C (NR), HDL-C (NR); IG – Healthy young: SBP (NR), DBP (NR), TG (NR), TC (NR), LDL-C (NR), HDL-C (NR); IG- Young at risk: SBP (NR), DBP (NR), TG (NR), TC (NR), LDL-C (NR), HDL-C (NR) | (∅) |
| Coates et al. (2020)^75^; Australia | P, 12 weeks | IG: 77; CG: 74 (78/128-completed) | IG: 64 ± 8, CG: 65 ± 8    IG: 30.3 ± 3.6, CG: 30.5 ± 3.8   Adults (women postmenopausal), with a BMI of 25–39.9 kg/m^2^ | Mixed (All: 31.9%; IG: 25.7%; CG: 38.0%) | Mixed (All: 18.4%; IG: 14.3%; CG: 22.5%) | Habitual diet with 15% energy from their EER from carbohydrate-rich snack foods (the original Scotch finger, Arnott’s biscuits, Australia and no-added-salt potato chips, Freedom Foods, Australia) | Habitual diet with 15% energy from their EER from whole raw natural almonds | SBP ↓, DBP →, TG →, TC →, LDL-C →, HDL-C → | (+) |
| Cogan et al. (2023)^68^; USA | P, 4 weeks | IG: 21 (15); CG: 23 (16) | IG: 59 ± 1, CG: 58 ± 1   IG: 25.1 ± 4.6, CG: 25.4 ± 1.0   Adult men and postmenopausal women | Unmedicated | Unmedicated | Free-living diet and diet instructions to avoid a written list of antioxidant or nitrate-rich foods, avoid consuming >3 alcoholic beverages/d (men) or >2/d (women), to consume <2 servings of red wine/week, and <2 servings of nut butter/week. CG also abstained from all nuts (peanuts and tree nuts). | Free-living diet and the same dietary instructions as CG except for consumption of 68 g/d pecans. | TG →, TC ↓, LDL-C ↓, HDL-C →, non-HDL-C ↓, Apo B ↓, Lp(a) → | (+) |
| Colquhoun et al. (1996)^153^; Australia | CR, 4 weeks | 14 (7) | All participants: 46.36 ± 10.44   NR  Adult with sedentary occupation (no specified health status) | Unreported | Unmedicated | Isocaloric diet compared with IG - A low-fat diet aimed to reduce total dietary fat to 20-25% of total energy intake | Isocaloric diet compared with IG - A high-fat diet (40-45% total energy from fat) with a large proportion of the fat intake derived from macadamia nuts (20% of total energy intake, ranged between 50-100g or 25-50 nuts per day). | TG (NR), TC (NR), LDL-C (NR), HDL-C (NR) | (∅) |
| Damasceno et al. (2011)^106^; Spain | CR, 4 weeks per arm | 18 (9) | All participants: 56 ± 13   All participants: 25.7 ± 2.3   Asymptomatic men and women with moderate hypercholesterolemia aged 25-75 years (after menopause in women), absence of chronic illnesses or secondary hypercholesterolemia. | Unreported | Unmedicated | Mediterranean diet with virgin olive oil, amount varying from 35-50g/d, depending on total energy requirements | IG- almond: Mediterranean diet with raw, shelled Spanish almonds (Marcona variety) amount varying from 50-75 g/d, depending on total energy intake **OR** IG: walnut: Mediterranean diet with Spanish grown walnuts (Serr/Chandler variety), amount varying from 40-65 g, depending on total energy intake | IG - almond: SBP →, DBP →, TG →, TC, LDL-C, HDL-C →, Apo A1 →, Apo B →, Lp(a) →; IG - walnut: SBP →, DBP →, TG →, TC, LDL-C, HDL-C →, Apo A1 →, Apo B →, Lp(a) → | (∅) |
| Damavandi et al. (2013)^125^; Iran | P, 8 weeks | IG: 23 (16); CG: 25 (17) | All participants: 55.68 ± 7.74   IG: 28.47±3.57, CG: 28.18±3.55   Patients diagnosed with type 2 diabetes mellitus | Unreported | Unreported | Follow own‑self-selected diet | A diet similar to CG, except with replacement of raw, unsalted hazelnuts with skins as a snack for 10% of total daily calorie intake | TG →, TC →, LDL-C →, HDL-C ↑ | (+) |
| Damavandi et al. (2019)^126^; Iran | P, 8 weeks | IG: 22 (15); CG: 21 (19) | All participants: 53.86 ± 7.22   IG: 28.7 ± 5.8, CG: 28.7 ± 5.8   Patients with type 2 diabetes mellitus | Unreported | Unreported | Maintain usual dietary pattern | Maintain usual dietary pattern and substitute 10% of their total calorie intake with unsalted cashews as snacks (approximately 28 g/day, based on baseline calculated energy intake) | TG (NR), TC (NR), LDL-C (NR), HDL-C (NR) | (∅) |
| de Oliveira Fialho et al. (2021)^85^; Brazil | P, 8 weeks  IG: whole peanut (WP) IG: skinned peanut (SP) - discuss in next section CG: no peanut (NP) | IG - whole peanut (WP): 8 (8); IG - skinned peanut (SP): 8 (8); CG: 8 (8) | All participants: 33.1 ± 8.7   All participants: 34.3 ± 3.7   Adult women with obesity (BMI > 30 kg/m^2^) at reproductive age, weight stable (±5 kg) during the previous 6 months, had good dental health, and had a constant level of physical activity | Unreported | Unreported | A restrictive diet (meets AMDR) providing 250 kcal/day less than the customary intake of their group means. | A restrictive diet similar to CG for the 2 IG. IG -WP: consume 56g/d whole roasted peanuts daily **OR** IG – SP: consume 56g/d skinned roasted peanuts *Peanuts were roasted at 180 °C for 20 minutes; peanuts were consumed 30 minutes before midday meal | IG-WP: SBP →, DBP →, TG →, TC ↓, LDL-C →, HDL-C →, VLDL-C →; IG-SP: SBP →, DBP →, TG →, TC ↓, LDL-C →, HDL-C →, VLDL-C → | (+) |
| Deon et al. (2018)^54^; Italy | P, 8 weeks | IG: HZN+S: 22 (10); HZN-S: 20 (11); CG: 18 (5) | IG - HZN+S: 10.8 ± 2.5; IG - HZN-S: 11.8 ± 2.8; CG: 12.2 ± 2.3   IG - HZN+S: 20.4 ± 4.0; IG - HZN-S: 20.3 ± 3.7; CG: 20.9 ± 3.9   Children and adolescents with primary hyperlipidemia | Unreported | Unmedicated | Only receiving nutritional recommendations based on the CHILD guidelines (55% of energy from carbohydrates, 15% from proteins, and 30% from fats (saturated fat 7-10%); dietary cholesterol <100 mg/1000 kcal and no more than 300 mg/d and 10-25 g/d of soluble fiber) | Receiving nutritional recommendations based on CHILD guidelines. IG - HZN+S: consumed 13-15g/d (adjusted on children's body weight (0.43 g/kg of body weight on average) roasted (31 minutes at 135 °C) hazelnut with skin (hazelnut with >80% of skin) **OR** IG - HZN-S: consumed 13-15g/d roasted hazelnut without skin | IG - HZN+S: SBP (NR), DBP (NR), TG (NR), TC (NR), LDL-C (NR), HDL-C (NR), non-HDL-C (NR); IG - HZN-S: SBP (NR), DBP (NR), TG (NR), TC (NR), LDL-C (NR), HDL-C (NR), non-HDL-C (NR) | (+) |
| Dhillon et al. (2018)^57^; USA | P, 8 weeks | IG: 38 (22); CG: 35 (19) | IG: All participants are 18 CG: All participants are 18 except one is 19   IG: 25.6 ± 5.0 CG: 25.3 ± 4.5   New college students (BMI: 18–41 kg/m^2^) with no cardiometabolic disorders | Unmedicated | Unmedicated | Usual diet with 5 sheets Graham crackers (77.5 g/d) (providing 338 kcal; 74% carbohydrate [2.5g fiber], 20% fat, 6% protein) | Usual diet with dry-roasted (129.4°C for 50 min) almond 56.7 g/d (providing 364 kcal; 14% carbohydrate [8 g fiber], 74% fat, 13% protein) | SBP →, DBP →, TG →, TC ↑, LDL-C →, HDL-C ↑ | (+) |
| Dhillon, Tan & Mattes (2016)^87^; USA | P, 12 weeks | IG: 43 (32); CG: 43 (33) | IG: 31.1 ± 12.9, CG: 31 ± 13.2   IG: 29.9 ± 3.2, CG: 40 ± 4.5   Healthy adults who were overweight and obese | Unreported | Unmedicated | A nut-free energy-restricted diet (500-kcal deficit/d). | An almond-enriched energy-restricted (500-kcal deficit/d) diet with 15% energy from dry roasted (at 129.4°C for 50 minutes) and lightly salted almonds | SBP →, DBP →, TG →, TC →, LDL-C →, HDL-C → | (∅) |
| Dikariyanto et al. (2020)^128^; United Kingdom | P, 6 weeks | IG: 54 (39/56-randomised); CG: 51 (36) | IG: 56.3 ± 10.3, CG: 56.0 ± 10.7   IG: 27.3 ± 4.4, CG: 26.7 ± 4.5   Free-living adult men and women with above-average risk of developing CVDs | Unreported | Unreported | Maintained habitual mealtime eating habits and fruit consumption and consumed sweet and savoury mini muffins (20% of EER) baked at the study center between meals. | Maintained habitual mealtime eating habits and fruit consumption and consumed dry-roasted whole non-salted almonds (20% of EER) between meals. | SBP →, DBP →, TG →, TC →, LDL-C ↓, HDL-C →, VLDL-C →, IDL-C →, non-HDL-C ↓, Apo A1 →, Apo B → | (+) |
| Dusanov et al. (2020)^146^; Norway | P, 6 months | IG: 42 (24); CG: 44 (25) | IG: 58.0 ± 5.2, CG: 55.0 ± 6.9   IG: 31.7 ± 3.1, CG: 31.2 ± 2.9   Men and women with BMI 25-38 kg/m^2^ and 1 component of metabolic syndrome in addition to WC (≥102 cm for men or ≥88 cm for women) | Mixed (IG: 47.6%, 20/42; CG: 34.1%, 15/44) | Mixed (IG: 16.7%, 7/42; CG: 15.9%, 7/44) | Usual diet without fatty fish and nuts (lean fish is allowed) | Usual diet with 30g/d nuts (approximately 100g walnuts, 50g hazelnuts and 50g almonds weekly, providing approximately 1400 kcal/week) | SBP →, DBP →, TG →, TC →, LDL-C →, HDL-C →, Apo B → | (+) |
| Eastman & Clayshulte (2005)^100^; USA | P, 8 weeks | IG: 8 (6) CG: 9 (7) | IG: 46 ± 6, CG: 53 ± 10   IG: 27 ± 4, CG: 27 ± 3   Adults with hyperlipidemia (both TC >200 mg/dL and LDL-C >130 mg/dL) or otherwise healthy adults. | Unreported | Unmedicated | Self-selected diets with the exception of not consuming nuts. | Self-selected diets avoiding nut consumption except for consuming 68 g/d (approximately 3/4 cup) of shelled pecans at any time in the day | TG →, TC →, LDL-C →, HDL-C → | (+) |
| Edwards et al. (1999)^51^; USA^#^ | CR, 3 weeks per arm | 10 (6) | Median age: 46 (Range: 28-64)   NR   Participants with moderate hypercholesterolemia | Unmedicated | Unmedicated | Regular diet | Regular diets and substituted roasted, unsalted pistachio nuts for 20% of their daily caloric intake. | TG (NR), TC (NR), LDL-C (NR), HDL-C (NR) | (-) |
| Fitó et al. (2014)^131;^ Spain^e^ | P, 1 year | IG: 310 (167); CG: 310(170) | IG: 66.2 ± 6.0, CG: 66.4 ± 5.7  IG: 29.5 ± 3.3, CG: 29.4 ± 3.4   Participants at high risk of CVD, but with no CVD at enrolment.  . | Mixed (ACE inhibitors: IG: 32.3%; CG: 26.5%; Diuretics: IG: 18.7%, CG: 20.8%) | Mixed (Lipid-lowering agents: IG: 39.7%; CG: 44.2%) | Mediterranean-style diet + virgin olive oil (1L/week) | Mediterranean-style diet + mixed nut (30g/d, including 15g/d of walnut, 7.5g/d of hazelnut, and 7.5g/d of almond) | Lp(a) (NR) | (∅) |
| Foster et al. (2012)^90^; USA | P, 18 months | IG: 61 (54); CG: 62 (58) | IG: 47.0 ± 12.02, CG: 46.7 ± 13.0   IG: 33.9 ± 3.5, CG: 34.0 ± 3.7   Overweight and obese individual with a BMI of 27–40kg/m^2^ | Unreported | Unmedicated | Low-calorie diet (providing 1200–1500 kcal/d for women and 1500–1800 kcal/d for men) | Low-calorie diet same as CG and consumed two 28g packages of almonds per day (~24 almonds per package; only whole, raw almonds were used for the first 5 weeks only, roasted almonds were introduced at week 6 and, a variety of isocaloric, flavored almonds were used over time). | SBP →, DBP →, TG →, TC →, LDL-C →, HDL-C →, VLDL-C → | (∅) |
| Gayathri et al. (2023)^94^; India | P, 12 weeks | IG: 174 (NR); CG: 178 (NR) | All participants: 38 ± 9   IG: 28.6 ± 3.7, CG: 28.2 ± 3.8   Adults who were overweight or obese (BMI≥23 kg/m^2^) | Unreported | Unreported | Habitual diet, avoid nuts in any form | Habitual diet and consumed 43 g/d almonds, either as a mid-morning or evening snack, after adjusting for carbohydrate or fat calories in their regular diet | SBP →, DBP →, TG →, TC ↓, LDL-C →, HDL-C →, Apo A1 →, Apo B → | (∅) |
| Gebauer et al. (2008)^97^; USA^a^ | CR, 4 weeks per arm | 28 (18) | All participants: 48 ± 1.5   All participants: 26.8 ± 0.7   Healthy, non-smoking adult with elevated LDL-C (≥2.86 mmol/L) | Unmedicated | Unmedicated | Step I diet (cholesterol-lowering diet with an average intake of 2500 kcal/d to maintain weight), consistent with food-based dietary recommendations | Step I diet. IG - 1PD: consumed 1 serving of roasted and salted pistachio (10% of total energy, dose-ranging 32-63 g/d) **OR** IG – 2PD: consumed 2 servings of roasted and salted pistachio (20% of total energy, dose-ranging 63-126 g/d) | 1PD: TG →, TC ↓, LDL-C ↓, HDL-C →, VLDL-C →, non-HDL-C ↓, Apo A1 →, Apo B ↓; 2PD: TG ↓, TC ↓, LDL-C ↓, HDL-C →, VLDL-C ↓, non-HDL-C ↓, Apo A1 →, Apo B ↓ | (∅) |
| Ghadimi Nouran et al. (2010)^103^; Iran | CR, 4 weeks per arm | 54 (0) | All participants: 43 ± 9.55   All participants: 27.5 ± 3.67   Male adults aged 25–65 years, with TC between 200–350mg/dl and mean TG lower than 400mg/dl. | Unmedicated | Unmedicated | Habitual diet with no specific dietary advice | Habitual diet with 60-93g/d freshly roasted, lightly salted peanuts (equivalent to approximately 20% of each participant’s mean energy intake), consumed with meals or as snacks | SBP →, DBP →, TG →, TC →, LDL-C →, HDL-C ↑ | (∅) |
| Ghanavati, Alipour & Nasrollahzadeh (2021)^27^; Iran | P, 8 weeks | IG:35 (16); CG:32 (14) | IG: 58 ± 7, CG: 59 ± 8   IG: 30 ± 3.7, CG: 31.8 ± 4.2   Overweight or obese, and with stable coronary artery disease | Mixed (B-blockers: 47.8%, 32/67; ACI/ARB: 67.2%, 45/67; CCB: 16.4%, 11/67; carvedilol - combined alpha and beta-blockers: 20.9%, 14/67) *this information was stated in Ghanavati et al. (2021)^26^ | Mixed (Statin: 95.5%, 64/67 - 31 patients in CG and 33 patients in IG) | Nut-free energy-restricted diet (25% calorie restriction of baseline energy requirements, 55% of the energy from carbohydrate, 18% from protein and 27% from fat) | Low-calorie (25% of energy deficit) nuts-containing diet, with mixed nuts with equal amounts of unsalted pistachios, almonds, and peanuts (20% of energy from mixed nuts and the remaining energy were distributed as 55% from carbohydrate, 18% from protein and 27% from fat) | TG →; TC →; LDL-C →; HDL-C ↑; Non-HDL-C →; Apo A1 ↑ | (∅) |
| Gozde et al. (2019)^99^; United Kingdom | P, 6 weeks | IG: 20 (10); CG: 17 (6) | IG: 47.1 ± 5.4, CG: 43.3 ± 6.2   IG: 25.64 ± 2.04, CG: 25.47 ± 3.16   Mild to moderate hyperlipidemic, non-smoking participants | Unreported | Unmedicated | AHA low-fat and low-cholesterol diet with no nut consumption | AHA low-fat and low-cholesterol diet supplemented with 40 g/d walnuts as a snack | TG →, TC →, LDL-C →, HDL-C ↑, VLDL-C → | (∅) |
| Guarneiri, Paton & Cooper (2021)^37^; USA | P, 8 weeks | IG – addition (ADD): 16 (11); IG – addition (SUB): 18 (13); CG: 18 (12) | IG - ADD: 49 ± 11, IG - SUB: 46 ± 10, CG: 50 ± 16  IG - ADD: 30.2 ± 4.1, IG - SUB: 32.5 ± 6.9, CG: 31.0 ± 7.1  Sedentary men and women, aged between 30-75 years with a high cholesterol or a BMI of ≥28kg/m^2^ | Unreported | Unmedicated | Free-living diet without nuts | Free-living diet. IG-ADD: consumed 68 g/d pecans as part of their free-living diet with no additional dietary instructions - **OR** IG-SUB: substituted the 470 kcal of food they habitually consumed in their usual diet with the 68g/d pecans. *Pecan (raw pecan, no roasting, cooking, or baking) was provided to participants. | IG-ADD: SBP →, DBP →, TG↓, TC↓, LDL-C↓, HDL-C →, non-HDL-C↓, Apo B↓, Lp(a) →; IG-SUB: SBP →, DBP →, TG↓, TC↓, LDL-C↓, HDL-C →, non-HDL-C↓, Apo B↓, Lp(a) → | (+) |
| Guarneiri, Paton & Cooper (2022)^38^; USA | P, 8 weeks | IG - addition (ADD): 30 (20); IG - c (SUB): (21); CG: 32 (23) | IG - ADD: 47 ± 10.95, IG - SUB: 44 ± 11.14, CG: 47 ± 11.31   IG - ADD: 27.6 ± 4.93, IG - SUB: 30.3 ± 6.68, CG: 28.1 ± 6.79   Sedentary men and women with a BMI of ≥18.5 kg/m^2^ | Unreported | Unmedicated | Free-living diet without nuts | Free-living diet. IG-ADD: consumed 68 g/d pecans as part of their free-living diet with no additional dietary instructions - **OR** IG-SUB: substituted the 470 kcal of food they habitually consumed in their usual diet with the 68g/d pecans. *Pecan (raw pecan, no roasting, cooking, or baking) was provided to participants. | IG-ADD: SBP →, DBP →; IG-SUB: SBP →, DBP → | (∅) |
| Gulati et al. (2014)^136^; India | P, 24 weeks | 68 IG: 33 (10) CG: 35 (21) | IG: 41.6 ± 8.4, CG: 43.3 ± 8.1   All participants: 30.9 ± 7.5 Kg/m^2^   Asian Indians with metabolic syndrome | Unreported | Unreported | A standard diet formulated according to the dietary guidelines for Asian Indians (60% of energy from carbohydrates, 15% from protein, and 25% from fat) | A standard diet formulated according to the dietary guidelines Asian Indians and substituted unsalted pistachios daily (20% energy) for visible fat, a portion of carbohydrates, and dairy products, resulting in a diet containing 51% energy from carbohydrates, 20% from protein, and 29% from fat. | TG →, TC ↓, LDL-C ↓, HDL-C → | (∅) |
| Gulati et al. (2023)^118^; India | P, 3 months | IG: 32 (16); CG: 34 (22) | IG: 41.3 ± 7.1, CG: 42.6 ± 9.1   IG: 31.6 ± 4.1, CG: 30.5 ± 4.2   Participants with prediabetes | Unreported | Unreported | A standard diet with lifestyle changes and dietary counselling | A standard diet with lifestyle changes and dietary counselling and consumed a premeal load of raw unsalted almonds (20 g, about 17-18 in number) 30 minutes before breakfast, lunch, and dinner, and chew it over 5 min, with 60g/d in total. | TG →, TC ↓, LDL-C ↓, HDL-C →, VLDL-C ↓ | (+) |
| Hernáez et al. (2017)^130^; Spain^e^ | P, 1 year | 200 IG: 100 (53); CG: 100 (44) | IG: 66.4 ± 6.93, CG: 66.3 ± 5.78   IG: 29.0 ± 3.76, CG: 30.1 ± 3.85   Population at high cardiovascular risk | Unreported | Unreported | Mediterranean-style diet + virgin olive oil (1L/week) | Mediterranean-style diet + mixed nut (30g/d, including 15g/d of walnut, 7.5g/d of hazelnut, and 7.5g/d of almond) | TG (NR), TC (NR), LDL-C (NR), HDL-C (NR), Apo A1 (NR), Apo B (NR) | (∅) |
| Hernandez-Alonso et al. (2014)^112^; Spain | CR, 4 months per arm | 49 (25/54-randomised) | All participants: 55 ± 6.23   All participants: 28.9 ± 2.56   Community-living prediabetic men and women with a BMI < 35 kg/m^2^ and fasting plasma glucose levels 100-125 mg/dL | Mixed (ACE inhibitors: 11.1%; B-blocker and other antihypertensive medications: 24.1%) | Mixed (Statins: 9.3%; Fibrates: 3.7%) | Isocaloric diet as IG (energy intake of other fatty foods, mostly olive oil, was added to replace energy from pistachios included in IG) | Isocaloric diet as CG with 57g/d pistachio (half roasted, and half roasted and salted) | SBP →, DBP →, TG →, TC →, LDL-C →, HDL-C →, VLDL-C → | (+) |
| Holligan et al. (2014)^47^; USA^a#^ | CR, 4 weeks per arm | 28 (18) | All participants: 48 ± 1.5   All participants: 26.8 ± 0.7   Healthy, non-smoking adult with elevated LDL-C (≥2.86 mmol/L) | Unmedicated | Unmedicated | Step I diet (cholesterol-lowering diet with an average intake of 2500 kcal/d to maintain weight), consistent with food-based dietary recommendations | Step I diet, IG - 1PD: consumed 1 serving of roasted and salted pistachio (10% of total energy, dose-ranging 32-63g/d) **OR** IG – 2PD: consumed 2 servings of roasted and salted pistachio (20% of total energy, dose-ranging 63-126 g/d) | 1 PD: Lp(a) →; 2 PD: Lp(a) → | (∅) |
| Holscher et al. (2018)^72^; USA | CR, 3 weeks per arm | 18 (8) | All participants: 53.1 ± 9.33   All participants: 28.8 ± 3.82   Healthy men and women | Unreported | Unreported | Baseline diet (54% of energy from carbohydrates, 17% from protein, 29% from fat) | Isocaloric diet as CG with 1.5 servings (42g/d) of walnut halves and pieces | TG →, TC ↓, LDL-C ↓, HDL-C → | (∅) |
| Hunter, Considine & Mattes (2021)^84^; USA | P, 6 months | IG: 69 (51); CG: 65 (50) | Only report data for individual subgroups: High android visceral adipose tissue (High VAT) group: IG: 44 ± 9.8; CG: 45 ± 9.4, High android subcutaneous adipose tissue (High SAT) group: IG: 35 ± 14.4; CG: 36 ± 9.6, High gluteal femoral adipose tissue (High GF) group: IG: 32 ± 9.4; CG: 29 ± 8.9   Only report data for individual subgroups: High VAT group: IG: 34.6 ± 5.4; CG: 32.6 ± 5.2, High SAT group: IG: 34.2 ± 5.3; CG: 33 ± 5.3, High GF group: IG: 33.1 ± 5.2; CG: 33.3 ± 5.4   Healthy men and women with a BMI ≥ 27 kg/m^2^, falling within body fat distribution criteria, weight stable (±5 kg) for 6 months before the start of the study, non-smokers | Unreported | Unreported | Usual diet with habitual breakfast and afternoon snack routines | Usual diet and consumed two 0.75oz packets of roasted, unsalted almonds per day (1.5oz/d in total): once with their habitual breakfast and once as their afternoon snack, providing 1130 kJ | TG →, TC →, LDL-C →, HDL-C → | (∅) |
| Hwang et al. (2019)^140^; South Korea | CR, 16 weeks per arm | 84 (42) | All participants: 39.44 ± 6.53   All participants: 27.08 ± 3.61   Participant was diagnosed as having Metabolic syndrome | Unmedicated | Unmedicated | Habitual diet and consumed isocaloric white bread (313.3kcal, 60.5g carbohydrate, 3.4g fat, and 10.2g protein) per day, as convenient during the daytime. | Habitual diet and consumed 45g/d walnut (305.4kcal, 6.4g carbohydrate, 28.9g fat, and 6.4g protein), as convenient during the daytime | SBP →, DBP →, TG →, TC →, LDL-C →, HDL-C ↑, Apo B → | (∅) |
| Iwamoto et al. (2002)^71^; Japan | CR, 4 weeks per arm | 40 (20) | M: 23.8 ± 3.13, F: 23.6 ± 4.92   M: 22.2 ± 0.5, F: 20.7 ± 0.5   Healthy men and women | Unmedicated | Unmedicated | Reference diet following the dietary allowance for 20–39-year-olds based on the fifth recommended dietary allowance in Japan (1995) and the nutrient intake for 20–39-year-olds based on the national nutritional survey of Japan (1997). | Diet was identical to the reference diet in CG except that the walnut diet substituted two servings of walnuts per day (25 or 27 g per serving, or 52 g of walnuts per 10.0 MJ) for portions of some foods in the reference diet. | SBP →, DBP →, TG →, TC ↓, LDL-C ↓ , HDL-C →, Apo A1 →, Apo B ↓ | (∅) |
| Jamshed et al. (2015)^134^; Pakistan | P, 12 weeks | IG - Pakistani Almonds (PA): 38 (14/50-randomised *only reported no. female in randomised cohort); IG - American Almonds (AA): 41 (12/50-randomised); CG: 34 (11/50-randomised) | IG - PA: 57 ± 11.3, IG - AA: 61 ± 10.6, CG: 61 ± 1.4   All participants: 76 ± 12   CAD patients with optimal LDL-C and low HDL-C | Unreported | Unreported | Participants were instructed not to consume other nuts *Did not discuss background diet | Participants were instructed to soak the almonds overnight remove the skin and eat them before breakfast. IG – PA: consumed 10g/d Pakistani almonds - **OR** IG-AA: consumed 10g/d American almonds *Did not discuss background diet | IG - PA group: SBP →, DBP →, TG ↓, TC ↓, LDL-C ↓, HDL-C ↑, VLDL-C ↓; IG - AA group: SBP →, DBP →, TG ↓, TC ↓, LDL-C ↓, HDL-C ↑, VLDL-C ↓ | (∅) |
| Jenkins et al. (2018)^122^; Canada | P, 3moths | IG: 39 (14/40-randomised); CG: 32 (13/39-randomised) | IG: 63 ± 8.9, CG: 61 ± 9.9   IG: 28.8 ± 4.5, CG: 29.4 ± 4.2   Men or postmenopausal women with type 2 diabetes who were taking glucose-lowering agents other than acarbose, and not taking insulin, with medications stable for the previous 3 months | Mixed (All types: IG: 58%; CG: 72%) | Mixed (All types: IG: 58%; CG: 77%) | General dietary advice based on NECP Adult Treatment Panel  III and ADA guidelines were given, and participants were told to reduce total food intake (especially carbohydrate intake) since the supplements in both groups replaced approximately 24% of energy. CG consumed muffins (2-4 muffins per day depending on energy requirement) made by whole wheat, sweetened with apple concentrate, and with no added sugar. | Similar dietary advice as CG. IG consumed mixed nuts (50-100g/d depends on the energy requirement) including unsalted and mostly raw almonds, pistachios, walnuts, pecans, hazelnuts, peanuts, cashews and macadamias. | SBP →, DBP →, TG →, TC →, LDL-C →, HDL-C →, non-HDL-C →, Apo A1 →, Apo B ↓ | (+) |
| Jenkins et al. (2002)^109^; Canada | CR, 1 months per arm | 27 (12) | All participants: 64 ± 9   All participants: 25.7 ± 3.0   Healthy hyperlipidaemic men and postmenopausal women | Mixed (Beta blockers: 11%, ACE inhibitors; Angiotensin II ATI receptor blockers: 4%; Thiazide diuretics 7%) | Mixed (Statin: 7%) | Self-selected low-fat therapeutic diet and included the whole-wheat muffins, amount varying based on the participants' EER, ranging from 2 to 4 whole-wheat muffins (100- 200g), with an average of 147 ± 6 g/d | Self-selected low-fat therapeutic diets and include whole raw unblanched almonds, amount varying based on participants’ EER, ranging from 50 to 100g of almonds, with an average of 73±3g/d | SBP →, DBP →, TG →, TC ↓, LDL-C ↓, HDL-C ↑, Apo A1 →, Apo B ↓, Lp(a) ↓ | (∅) |
| Jones et al. (2023)^154^; USA | CR, 8 weeks per arm | 35 (28) | All participants: 62.1 ± 8.2   All participants: 30.3 ± 3.4   Overweight and obese men and women with abdominal obesity and at least one additional cardiometabolic risk factor | Mixed (NR) | Unmedicated | Usual diet abstaining from all nuts and limiting seed intake | Usual diet and consumed macadamia nuts to make up 15% of daily energy intake | TG →, TC →, LDL-C →, HDL-C → | (∅) |
| Jung et al. (2018)^80^; Korea | CR, 4 weeks per arm | 84 (73) | All participants: 52.4 ± 0.6   All participants: 25.4 ± 0.22   Overweight/obese adults with BMI 23–29.9 kg/m^2^ or WC ≥85 cm for women, ≥90 cm for men, and free of any diagnosed chronic disorders or acute inflammatory diseases for 2 years | Unreported | Unmedicated | Habitual diet without nuts and consumed 70g/d isocaloric home-made cookies as snacks | Habitual diet and consumed 56g/d roasted almonds as snacks | SBP →, DBP →, TG →, TC ↓, LDL-C ↓, HDL-C →, non-HDL-C ↓, Apo A1 →, Apo B → | (+) |
| Kasliwal et al. (2015)^105^; India | P, 3 months | IG: 21 (3/29-randomised *no. female ony reported in randomised cohort); CG: 21 (7/27-randomised) | IG: 37.7± 7.6, CG: 40.4 ± 8.2   IG: 26.1 ± 2.9, CG: 27.8 ± 4.7   Adults aged between 25-60 years with mild dyslipidemia, and no diabetes or CVD | Unmedicated | Unmedicated | Lifestyle modification (LSM) alone, with exercise and diet counselling (Amercian Heart Association recommended diet which aimed at providing 50-55% of energy from carbohydrates, 15-18% from proteins, and 25-30% from fats) | LSM (same as CG) with substitution of 80 g/d (in-shell) pistachios (equivalent to 40 g shelled pistachios) for visible fat (cooking oil and butter), a portion of carbohydrates, and dairy products. | SBP →, DBP →, TG →, TC →, LDL-C →, HDL-C →, Apo A1 ↓, Apo B → | (∅) |
| Katz et al. (2012)^148^; USA | CR, 8 weeks per arm | 46 (28) | All participants: 57.4 ± 11.9   All participants: 33.2 ± 4.4   Non-smoking adults with a BMI >25kg/m^2^ and a WC >40 inches for men or >35 inches for women, and exhibited 1 or more additional risk factors for metabolic syndrome | Unreported | Unreported | An *ad libitum* diet without walnuts | A walnut-enriched *ad libitum* diet (56 g/d shelled, unroasted English walnuts as a snack or with a meal) | SBP →, DBP →, TG →, TC →, LDL-C →, HDL-C → | (∅) |
| Khorramirad et al. (2021)^49^; Iran^#^ | CR, 12 weeks | 44 (NR) | Group A (followed IG and then CG): 53.1; Group B (followed CG and then IG): 50.1 *did not report whether SD or SEM is used in the study   Group A (followed IG and then CG): 30.3, Group B (followed CG and then IG): 31.8 *did not report whether SD or SEM is used in the study   Patients with diabetes for at least 1 year | Unreported | Unreported | Routine diet | Routine diet and consumed 50g/d raw pistachios at morning and evening snack times (pre-packaged in 2 bags with a total of 50g/d) | SBP ↓, DBP →, TC →, TG →, LDL-C →, HDL-C → | (-) |
| Kocyigit, Koylu & Keles (2006)^52^; Turkey | P, 3 weeks | IG: 22 (10); CG: 22 (10) | IG: 32.8 ± 6.7, CG: 33.4 ± 7.2   IG: 24.2 ± 6.1, CG: 24.6 ± 5.6   Healthy volunteers (doctors and nurses) with normal lipid levels | Unmedicated | Unmedicated | Regular diet | Regular diet and consumed pistachio nuts as 20% of the total daily energy intake (ranging from 65 to 75g/d depending on the subject’s energy intake). | TG →, TC ↓, LDL-C →, HDL-C ↑ | (∅) |
| Kurlandsky & Stote (2006)^62^; USA | P, 6 weeks | IG: 12 (12); CG: 12 (12) | IG: 41.8 ± 11.7, CG: 51.3 ± 6.3   IG: 25.3 ± 3.5, CG: 26.1 ± 4.1   Healthy women with no previous history of hypertension, atherosclerosis, or metabolic diseases | Unreported | Unmedicated | Self-selected diet (25% to 35% of total energy from fat, with less than 7% SFA, up to 10% PUFA, and up to 20% MUFA) and avoided consumption of nuts and chocolate. | Self-selected diet same as CG with 60g/d almonds | TG →, TC →, LDL-C →, HDL-C → | (∅) |
| Le et al. (2016)^81^; USA^b^ | P, 6 months | IG: 71 (NR); CG: 66 (NR)* only report 245 females for the whole study including other arms | IG: 51 (Range: 22–67), CG: 50 (Range: 25–72)  IG: 33.6 ± 3.6, CG: 33.6 ±3.6   Overweight and obese women with a BMI 27-40 kg/m^2^ | Unreported | Mixed (All types: 11.8%, 29/ 245) | A lower carbohydrate (45% of total energy), higher fat (35% of total energy) diet with a reduction in energy intake (-500 to -1000 kcal/d deficit with an individualised prescribed diet plan 1200, 1500, or 1800 kcal/d). | A walnut-rich (42g/d), lower carbohydrate (45% of total energy), higher fat (35% of total energy) diet with a reduction in energy intake (-500 to -1000 kcal/d deficit with an individualised prescribed diet plan 1200, 1500, or 1800 kcal/d). | TG (NR), TC (NR), LDL-C (NR), HDL-C ↑ | (∅) |
| Lee et al. (2014)^141^; South Korea | P, 6 weeks | IG: 30 (NR); CG: 30 (NR) | NR (age range: 35-65)   IG: 27.19 ± 2.11, CG: 26.96 ± 2.16   Individuals with a BMI at least 23 kg/m^2^ and met the criteria for metabolic syndrome, defined according to the NCEP Adult Treatment Panel III criteria, except abdominal obesity was defined using the Korean-specific cutoff values (WC ≥90 cm for men or ≥85 cm for women) | Unreported | Unreported | Dietary recommendations for prudent diet | Usual diet and consumed 30g/d mixed nuts (15, 7.5, and 7.5g/d of raw walnuts, raw pine nuts, and roasted peanuts, respectively). | SBP →, TG →, TC →, LDL-C →, HDL-C →, non-HDL-C → | (∅) |
| Lee et al. (2017)^93^; USA | CR, 4 weeks per arm | 31 (13) | All participants: 46.3 ± 10.02   All participants: 29.6 ± 2.78   Overweight and obese individuals | Unreported | Unmedicated | Average American Diet, composed of the same foods as the other diets, with the exception of almonds, dark chocolate, and cocoa. | Almond diet (42.5 g/d raw almonds, 253 kcal/d), lower in saturated fatty acids (8% compared with 13%) and higher in MUFA (16% compared with 13%) and PUFA (9% compared with 7%) compared with CG | SBP →, DBP →, TG →, TC ↓, LDL-C ↓, HDL-C →, VLDL-C →, IDL-C →, non-HDL-C ↓, Apo A1 →, Apo B →, Lp(a) → | (∅) |
| Li et al. (2010)^67^; USA | P, 12 weeks | IG: 27 (28/36-randomised *only reported in randomised cohort); CG: 25 (29/34-randomised) | IG: 45.4 ± 12.00m CG: 47.3 ± 13.41   IG: 30.1 ± 2.40, CG: 30.9 ± 2.33   Individuals in good health | Unreported | Unmedicated | Isocaloric weight reduction diets (-500 calories per day less than resting metabolic rate) with an afternoon snack of 56 g/d salted pretzels (220 calories) | Isocaloric weight reduction diets (-500 calories per day less than resting metabolic rate) with an afternoon snack of 53 g/d salted pistachios (240 calories) | TG ↓, TC →, LDL-C →, HDL-C → | (+) |
| Liu et al. (2017)^36^; South Korea^d^ | P, 16 weeks | IG - pre-meal (PM): 58 (31); IG -snack (SN): 55 (32); CG: 56 (29) | All participants: 26.33 ± 5.551   IG-PM: 22.96 ± 2.86; IG-SN: 22.88 ± 3.06 CG: 21.92 ± 3.15   Healthy and young adults who are non-smokers with a BMI of 17-30 kg/m^2^ | Unmedicated | Unmedicated | Regular meal + consumed 66 g of commercial cookies, a high-carbohydrate control food item which has similar calories as almond | Regular meal. IG-PM: consumed 56 g/d almonds before regular meals, and consume nearly 1/3 amount of almonds before each meal **OR** IG-SN: consumed 56 g/d almonds between meals as snacks (two hours before and after meals) | IG-PM: TG →, TC →, LDL-C →, HDL-C →, non-HDL-C →; IG-SN: TG →, TC →, LDL-C ↓, HDL-C →, non-HDL-C ↓ | (∅) |
| Liu et al. (2018)^59^; South Korea^d^ | P, 20 weeks | IG (have peanut premeal or as a snack): 57 (26); CF: 28 (14) | IG: 26.96 ± 5.22, CG: 26.14 ± 5.40   IG: 23 ± 3.17, CG: 21.66 ± 3.08   Healthy and young adults who are non-smokers with a BMI of 17-30 kg/m^2^ | Unmedicated | Unmedicated | Regular meal + consumed 66 g of commercial cookies, a high-carbohydrate control food item which has similar calories as almond | Regular meal. IG-PM: consumed 56 g/d almonds before regular meals, and consume nearly 1/3 amount of almonds before each meal **OR** IG-SN: consumed 56 g/d almonds between meals as snacks (two hours before and after meals) | SBP →, DBP →, TG ↓, TC ↓, LDL-C ↓, HDL-C ↓, VLDL-C ↓, non-HDL-C ↓ | (∅) |
| Lovejoy et al. (2002)^39^; United States | CR, 4 weeks per arm | 30 (17) | All participants: 53.8 ± 10.41   All participants: 33.0 ± 5.48   Men and women with type 2 diabetes | Unreported | Unmedicated | CG-High-fat control diet (37% energy from total fat, 10% from the MUFAs olive or canola oil) **OR** CG-Low-fat control (25% energy from total fat, 10% from olive or canola oil) | IG-High-fat, high-almond diet (37% energy from total fat, 10% from almonds) **OR** IG-low-fat, high-almond diet (25% energy from total fat, 10% from almonds). *Both diets provided 57–113 g/d almonds depending on the total energy level. | TC (NR), LDL-C (NR), HDL-C (NR) | (∅) |
| Ma et al. (2010)^123^; USA | CR, 8 weeks per arm | 24 (14) | All participants: 58.1 ± 9.2   All participants: 32.5 ± 5.0   Participants with a clinical diagnosis of type 2 diabetes for at least 1 year but no more than 5 years, were nonsmokers, had serum glucose levels and medication doses that had been stable for 3 months, and were not currently receiving insulin therapy | Mixed (All types: 70.8%; lisinopril: 37.5%; verapamil: 4.2%; irbesartan: 4.2%; atenolol: 4.2%; metoprolol: 8.3%; valsartan: 4.2%; enalapril: 4.2%; hydrochlorothiazide/moexipril: 4.2%; nadolol: 4.2%; olmesartan: 4.2%) | Mixed (All types: 54.2%; atorvastatin: 20.8%; pravastatin: 8.3%; simvastatin: 8.3%; fenofibrate: 4.2%; lovastatin: 4.2%; rosuvastatin 4.2%; ezetimibe/simvastatin: 4.2%) | An ad libitum diet without walnuts | An ad libitum diet enriched with 56g/d (366 kcal) shelled, unroasted English walnuts. | SBP ↑, DBP ↑, TG →, TC →, LDL-C →, HDL-C → | (∅) |
| Madan et al. (2021)^115^; India | P, 12 weeks | IG: 107 (121/146-randomised); CG: 112 (95/129-randomised) | Range: 16-25 years old, no average age reported   IG: 23.7 ± 5.4, CG: 22.4 ± 4.8   Community living adolescents and young adults with impaired fasting glucose levels and 2-hour post-glucose value and/or fasting hyperinsulinemia or glucose challenge hyperinsulinemia | Unreported | Unreported | Regular diet with 61-65g/d of commonly consumed isocaloric (equal to 56g of raw almonds) Indian savory snacks (2 varieties), prepared using whole wheat flour, chickpea flour, salt, and Indian spices. | Regular diet with 56g/d of raw almonds (providing ∼20% of the daily energy intake i.e., 340 kcals/d), separated in 2 packets, consumed as snacks in a day. | TG →, TC ↓, LDL-C ↓, HDL-C →, VLDL-C → | (+) |
| Mah et al. (2017)^111^; USA | CR, 28 days per arm | 42 (31/51-randomised) | All participants: 55.7 ± 10.14   All participants: 26.9 ± 2.79   Normally active men and women (nonpregnant and nonlactating), with or at risk of high LDL-C | Unreported | Unmedicated | Weight-maintenance dietary program with baked potato chips (Lays Baked Chips; PepsiCo) (approximately 11% of total kilocalories; 32–64g/d). | Weight-maintenance dietary program same as CG with roasted, salted cashews (approximately 11% of total kilocalories; 28–64g/d) | TG →, TC ↓, LDL-C ↓, HDL-C →, non-HDL-C ↓ | (+) |
| Maranhao et al. (2011)^89^; Brazil | P, 16 weeks | IG: 8 (8); CG: 9 (9) | All participants: 15.4 ± 2.0   All participants: 35.6 ± 3.3   Female adolescent with obesity who were being above the 95th percentile for BMI according to age | Unmedicated | Unmedicated | Usual diet with 1 placebo capsule (containing lactose)/d | Usual diet with 15-25 g/d (equivalent to 3 to 5 units/day, calculated to achieve 10% of energy from MUFAs in the diet) of Brazil nuts; Nuts were consumed as snacks or with meals in salads | TG ↓, TC ↓, LDL-C ↓, HDL-C → | (∅) |
| McKay et al. (2018)^88^; USA | CR, 4 weeks | 26 (5) | M: 57.9 ± 4.58, F: 67.4 ± 6.48   M: 29.4 ± 3.21, F: 28.4 ± 2.01   Non-smoking men and postmenopausal women who are overweight or obese with central adiposity | Unmedicated | Unmedicated | A typical American diet. The calorie content of the diet was adjusted to meet the energy needs of each subject while maintaining their initial body weight. | Similar diet as CG with whole pecans substituted for 15% of the total energy (~42.5g/ 2000kcal) | SBP →, DBP →, TG →, TC →, LDL-C →, HDL-C →, VLDL-C → | (+) |
| Mohan et al. (2018)^119^; India | P, 12 weeks | IG: 129 (51); CG: 140 (73) | IG: 51.3 ± 8.8, CG: 50.4 ± 10.1   IG: 25.6 ± 2.8, CG: 26.2 ± 3.9   Asian Indian with Type 2 Diabetes | Mixed (All types: 13.4%, 36/269) | Mixed (All types: 12.3%, 33/269) | A standard diabetic diet (The Asian Indian diabetic diet (1400–1600 kcal/d) - 60–65% energy from carbohydrates, 15–25% from fat, and the remaining calories from protein) only | A standard diabetic diet same as CG with 30g/d unsalted, raw, broken cashew nuts (consumed as a mid-morning or evening snack); replaced equivalent calories from carbohydrates in meal | SBP ↓; DBP →; TG →; TC →; LDL-C →; HDL-C ↑; VLDL-C → | (+) |
| Moreira Alves et al. (2014)^91^; Brazil | P, 4 weeks | IG - conventional peanuts (CVP): 22 (0); IG - high-oleic peanuts (HOP): 21 (0); CG: 22 (0) | IG - CVP: 27.6 ± 7.19, IG - HOP: 27.2 ± 7.84, CG:27.1 ± 7.50   IG - CVP: 29.5 ± 1.92, IG - HOP: 30.1 ± 2.45, CG:29.7 ± 2.81   Overweight and obese men with stable weight in the previous 3 months | Unreported | Unmedicated | Hypocaloric diet (250kcal was subtracted from the dietary prescription, 30% energy from fat, and 55% from carbohydrate) | Hypocaloric diet same as CG. IG - CVP – consumed the diet with 56g/d of CVP once at any time of the day - **OR** IG-HOP – consumed the diet with 56g/d of HOP once at any time of the day. | TG (NR), TC (NR), LDL-C (NR), HDL-C (NR), VLDL-C (NR) | (∅) |
| Morgan & Clayshulte (2000)^66^; USA | P, 8 weeks | IG: 10 (7); CG: 9 (8) | IG: 45 ± 10, CG: 37 ± 12   IG: 24 ± 5, CG: 24 ± 4   Healthy individuals with normal lipid levels | Unreported | Unmedicated | Self-selected diet with no nuts | Self-selected diet with 68g/d pecan - shelled pecan halves, consumed at any time during the day, either at meals and for snacks (contributing 459kcal and 44g fat per day) and no other nuts | TG →, TC ↓, LDL-C ↓, HDL-C ↑ | (∅) |
| Morgan et al. (2002)^110^; USA | CR, 6 weeks per arm | 42 (25) | All participants: 55.7 ± 11.8   All participants: 27.7 ± 5.8   Free-living community of healthy men and women with TC > 5.2mmol/L without evidence of CVD | Unreported | Unreported | Low-fat (<30% energy from fat, <14% from saturated fat), low-cholesterol diet (<200mg dietary cholesterol/day) (American Heart Association [AHA] Step I diet as defined by the National Cholesterol Educational Panel) | Diet similar to CG with 64g/d walnuts. Ways to substitute walnuts with other fat sources in the diet were instructed by a dietitian. | TG (NR), TC (NR), LDL-C (NR), HDL-C (NR) | (∅) |
| Mukuddem-Petersen et al. (2007)^135^; South Africa | P, 8 weeks per arm | IG: Walnut diet: 21 (11); IG: Cashew diet: 21 (13); CG: 22 (11) | IG: Walnut diet: 45 ± 10.76; IG: Cashew diet: 46 ± 10.98; CG: 45 ± 9.59   Walnut diet: 36·0 ± 5.9; Cashew diet: 34·4 ± 4.8; CG: 35·1 ± 5.2   Caucasian volunteers with the metabolic syndrome | Mixed (NR) | Mixed (NR) | A control diet (47% energy from carbohydrate, 20% from protein, 33% from fat) without any nuts or nut-based ingredients | Same diet as CG. IG – walnut diet: substituted 20% of energy with walnut **OR** IG – cashew diet: substituted 20% of energy with unsalted cashew (ranging from 63 to 108g/d) | Walnut diet: SBP (NR), DBP (NR), TG (NR), TC (NR), LDL-C (NR), HDL-C (NR); cashew nut diet: SBP (NR), DBP (NR), TG (NR), TC (NR), LDL-C (NR), HDL-C (NR) | (+) |
| Mustra Rakic et al. (2022)^70^; USA | P, 6 months | IG - 1.5 oz Almonds: 19 (9); IG - 3 oz Almonds: 24 (10); CG: 17 (8) | IG - 1.5 oz Almonds: 61.6 ± 6.3, IG - 3 oz Almonds: 60.4 ± 6.8, CG: 63.0 ± 5.6   IG - 1.5 oz Almonds: 28.9 ± 2.9, IG - 3 oz Almonds: 28.9 ± 2.8, CG: 29.2 ± 2.6   Healthy, non-smoking men and post-menopausal women | Unreported | Unreported | Maintain usual dietary habits, consumed 3.5oz/d (100g) of a snack mix containing cereal party mix, coconut, meat jerky, and butter. | Maintain usual dietary habits. IG- 1.5oz almond: consumed 1.5oz/d (42g) almonds **OR** IG-3oz almond: consumed 3oz/d (84g) almonds | IG-1.5oz almonds: TG (NR), TC (NR), LDL-C (NR), HDL-C (NR), VLDL-C (NR); IG- 3oz almonds: TG (NR), TC (NR), LDL-C (NR), HDL-C (NR), VLDL-C (NR) | (+) |
| Njike et al. (2015)^40^; USA | CR, 6 months (P, 12 months, Calorie-adjusted diet group [CADG] and Ad libitum diet group [ALDG] each for 12 months; In each group of the parallel design, further cross-over study with another 2 subgroups - 6 months per arm) | CADG: 56 (39); Ad libitum diet ALDG: 56 (42) | CADG: 56.5 ± 11.7, ALDG: 53.3 ± 11.1   CADG: 30.0 ± 4.0, ALDG: 30.2 ± 4.1   Participants who were non-smokers and had a high risk of Type 2 diabetes | Unreported | Unreported | CG - CADG: consumed a calorie-adjusted diet while avoiding walnuts and specific walnut-containing products **OR** CG – ALDG: consumed an ad libitum diet while avoiding walnuts and specific walnut-containing products | IG - CADG: consumed 392 g of walnuts per week (56 g or 2 oz/d, providing 366 kcal) in their meal plan which was instructed by dietitian to preserve an isocaloric condition after the addition of walnuts **OR** IG – ALDG: consumed 392g of walnuts per week (56g or 2oz/d, providing 366kcal) to include in their diet - the caloric intake was not monitored or regulated, and thus was allowed to float ad libitum | CADG: SBP →, DBP →, TG →, TC →, LDL-C →, HDL-C →; ALDG: SBP →, DBP →, TG →, TC →, LDL-C →, HDL-C → | (+) |
| Nora et al. (2023)^95^; USA | P, 16 weeks | IG: 14 (7); CG: 15 (7) | IG: 28.3 ± 10.1, CG: 26.8 ± 8.5    IG: 31.3 ± 5.36, CG: 30.0 ± 5.02   Men and women with overweight or obesity (BMI 25-40 kg/m^2^) | Unreported | Unreported | Usual diet with one serving - 69g/d isocaloric pretzels. | Usual diet with one serving - 42.5g/d packets of mixed nuts consisting of 25.5g of nut mixture (cashews, almonds, macadamia nuts, Brazil nuts, pecans), 5g of pistachios, 5g of walnuts, and 7g of peanuts | SBP →, DBP →, TG →, TC →, LDL-C →, HDL-C → | (+) |
| Palacios et al. (2020)^117^; USA | CR, 6 weeks per arm | 33 (16) | All participants: 48.3 ± 12.64   All participants: 30.5 ± 4.02   Men and women met the criteria for having prediabetes | Unreported | Unreported | Diet with habitual energy intake to maintain body weight and incorporated isocaloric CHO-based foods (approximately 480kcal/d, including fruit and grain bars, baked potato chips, mini bagels, rice cakes, dinner rolls, pretzels, dried mango slices, pudding cups, and vanilla cookie wafers) | Diet with habitual energy intake to maintain body weight and incorporated 1.5oz of raw almonds twice per day (approximately 480kcal/d) | SBP →, DBP →, TG →, TC →, LDL-C →, HDL-C , non-HDL-C →, Apo A1 →, Apo B → | (∅) |
| Parham et al. (2014)^121^; Iran | CR, 12 weeks per arm | 44 (33) | Group A (IG and then CG): 53 ± 10, Group B (CG and then IG): 50 ± 11   Group A (IG and then CG): 32.16 ± 6.58, Group B (CG and then IG): 30.24 ± 4.03   Patients with type 2 diabetes | Unreported | Unreported | Usual diet without nuts including pistachio nuts | Usual diet with 50g/d pistachio, separated into 2 snacks (25g each) and consumed in the morning and evening | SBP →, DBP → | (+) |
| Petersen et al. (2022)^116^; Australia | P, 6 months | IG: 57 (41); CG: 50 (29) | IG: 59 ± 14, CG: 58 ± 15   IG: 33.1 ± 4.9, CG: 33.0 ± 6.0   Adults were at moderate or high risk of type 2 diabetes | Mixed (All types: 13%, 14/107; IG 9%, 5/57; CG: 18%, 9/50) | Unreported | A low-fat diet with dietetic counseling to restrict energy intake (women: <5500 kJ/1300 kcal/d; men: <7000 kJ/1700 kcal/d) and avoid peanuts and peanut butter. | A weight loss diet. Received dietetic counseling to restrict energy intake (women: <5500 kJ/1300 kcal/d; men: <7000 kJ/1700 kcal/d), and consumed 35g of lightly salted dry-roasted peanuts before two main meals (70 g/d of peanuts in total). | SBP →, DBP → | (+) |
| Rajaram et al. (2009)^152^; USA | CR, 4 weeks per arm | 25 (11) | All participants: Range: 23-65 years old, no average age reported   All participants: 24.8 (Range: 18.7-36.6, no SD reported)   Normolipidemic to mildly hyperlipidemic but apparently healthy men and women, without a history of chronic or metabolic diseases | Unreported | Unmedicated | A diet followed the dietary guidelines for Americans, with 25–35% energy from fat, <10% energy from SFAs, and <300 mg cholesterol, with no nuts or fish. | Isocaloric diet as CG except those walnuts (42.5g/d, 6 days/ week) were substituted for meats and dairy foods. Walnuts were consumed during breakfast and/or dinner, alone or on cereals, and in salads or desserts. | TG →, TC ↓, LDL-C ↓, HDL-C →, Apo A1 →, Apo B ↓ | (∅) |
| Rock et al. (2017)^77^; USA | P, 6 months | IG: 47 (31/49-randomised); CG: 50 (27/51-randomised) | IG: 53.3 ± 9.8 CG: 52.2 ± 11.4   IG: 32.4 ± 3.5 CG: 32.4 ± 2.9   Non-diabetic overweight and obese men and women aged 21 years and older with BMI 27 - 40 kg/m^2^ | Unreported | Mixed (All types: 10%) | An energy-reduced diet with 500 to 1000 kcal/d energy deficit relative to energy expenditure | The energy-reduced diet similar as CG with an average of 42g/d walnuts for a diet containing ≥1500 kcal/day, or 28g of walnuts/ day for a diet containing <1500kcal/day (approximately 15% of total energy intake) | SBP →, DBP →, TG →, TC →, LDL-C →, HDL-C ↑ | (+) |
| Rock et al. (2016)^82^; USA^b^ | P, 1 year | IG: 71 (NR); CG: 66 (NR)* only report 245 females for the whole study including other arms | IG: 51 (Range: 22–67), CG: 50 (Range: 25–72)  IG: 33.6 ± 3.6, CG: 33.6 ±3.6   Overweight and obese women with a BMI of 27-40 kg/m^2^ | Unreported | Mixed (All types: 11.8%, 29/ 245) | A lower carbohydrate (45% of total energy), higher fat (35% of total energy) diet with a reduction in energy intake (500- to 1000-kcal/d deficit relative to expenditure with an individualised prescribed diet plan 1200, 1500, or 1800 kcal/d). | A walnut-rich (42g/d), lower carbohydrate (45% of total energy), higher fat (35% of total energy) diet with a reduction in energy intake (500- to 1000-kcal/d deficit relative to expenditure with an individualised prescribed diet plan 1200, 1500, or 1800 kcal/d). | TG (NR), LDL-C(NR), HDL-C (NR) | (∅) |
| Rock et al. (2020)^74^; USA | P, 4 months | IG: 49 (30/50*); CG: 47 (32/50-randomised) | IG: 55.0 ± 11.3; CG: 56.2 ± 10.6   IG: 32.8 ± 4.2; CG: 32.8 ± 3.5   Non-diabetic overweight/obese adults from the community | Unreported | Unreported | The group-based behaviour weight loss intervention including a diet achieving a deficit of 500-100kcal/d with the aim of weekly 1-2 pounds weight loss. | The group-based behaviour weight loss intervention including a diet achieving a deficit of 500-100kcal/d with the aim of weekly 1-2 pounds weight loss and consumed 42g (or 18% of energy intake) of roasted, shelled, unsalted pistachio. | SBP →, DBP →, TG →, TC →, LDL-C →, HDL-C → | (∅) |
| Ros et al. (2004)^96^; Spain | CR, 4 weeks per arm | 20 (12) | 55 (range: 26 - 75)   BMI: NM, Weight: 70.6 ± 10.3 kg   Non-smoking, moderate hypercholesterolemia, serum LDL-C ≥3.36 mmol/L (130 mg/dL), TG ≤2.82 mmol/L | Unreported | Unmedicated | Mediterranean diet | Mediterranean diet similar to CG with walnuts (varying from 40-65g; 18% total energy and 32% of the energy from MUFA from walnut) partially replaced olive oil and other MUFA–rich foods such as olives and avocados | SBP →; DBP →; TG →; TC ↓; LDL-C ↓; HDL-C →; VLDL-C →; Apo A1 →; Apo B → | (∅) |
| Ruisinger et al. (2015)^101^; USA | P, 4 weeks | IG: 22 (10); CG: 26 (14) | IG: 60.0 ± 10.4 CG: 59.3 ± 11.7   IG: 29.8 ± 4.8 CG: 28.6 ± 3.9   Taking chronic statin therapy with a consistent statin dose for at least 8 weeks before study entry | Unreported | Medicated (Statin: 100%) | Solely the NCEP adult treatment panel's third report therapeutic lifestyle changes diet counselling (emphasizes a reduction in SFAs and moderate consumption of unsaturated fats, specifically MUFA and PUFA). | The NCEP adult treatment panel's third report therapeutic lifestyle changes diet counselling with 100g/d raw and unsalted almonds. | TG →; TC →; LDL-C →; HDL-C →; VLDL-C ↓; IDC - C ↓; Non-HDL-C ↓; Lp (a) → | (∅) |
| Sabate et al. (1993)^73^; USA | CR, 4 weeks per arm | 18 (0) | All participants: 30 (range: 21-43)   All participants: 23.8 (range: 18.7-30.6)   Healthy men | Unmedicated | Unmedicated | A cholesterol-lowering diet designed according to the recommendations of the Expert Panel on the Step 1 diet of the NCEP. | Same diet as CG, and contains identical foods and macronutrients, except that 20% of the calories are from walnuts (replace other fatty foods, meat, and visible fat [oil, margarine, and butter]). | SBP →, DBP →, TG →, TC ↓, LDL-C ↓, HDL-C ↓ | (∅) |
| Sabate et al. (2003)^60^; USA | CR, 4 weeks per arm | 25 (11) | All participants: 41 ± 13   NR   Healthy men and women | Unreported | Unmedicated | Step-1 diet with no almonds. | Isoenergetic step-1 diet similar to CG. IG-low almond diet: 10% of energy was replaced by almond **OR** IG–high almond diet: 20% of energy was replaced by almond | IG-low almond diet: TG →, TC →, LDL-C →, HDL-C →, Apo A1 →, Apo B →, Lp(a) →; IG-high almond diet: TG →, TC ↓, LDL-C ↓, HDL-C →, Apo A1 →, Apo B ↓, Lp(a) → | (∅) |
| Sanchis et al. (2020)^55^; Spain | CR, 30 days per arm | 13 (7) | All participants: 71.33 ± 9.14   IG: 27.67 ± 6.64, CG: 30.33 ± 7.48   Patients had chronic kidney disease (CKD) stage 3 or 4 and were not undergoing renal replacement therapy. | Mixed (ACE inhibitors/ angiotensin II receptor-blocking agents: 69.2%; Beta-blockers: 38.5%; Calcium antagonists: 46.2%; Furosemide or triamterene: 30.8%; Thiazides: 7.7%; Potassium sparing diuretics: 7.7%) | Mixed (Statins: 61.5%; Fibrates: 7.7%) | A diet plan for people with CKD which were 2000kcal or 1650kcal, according to the caloric requirement of each patient is given by the dietitian. Consumed 60g of unsalted white bread with 5g of olive oil in the middle morning | Same diet plan as CG and consumed 30g/d walnuts in the middle morning. | SBP ↓, DBP →, TG →, TC →, LDL-C →, HDL-C → | (+) |
| Sapp, Kris-Etherton & Petersen (2022)^113^; USA | CR, 6 weeks per arm | 50 (25) | All participants: 42 ± 15   All participants: 28.3 ± 5.6   Men and women who had elevated fast plasma glucose measured at screening, were nonsmokers, and had a BMI ≥20 and ≤40 kg/m^2^ | Unmedicated | Unmedicated | Background diet, and consumed isocaloric lower fat, higher carbohydrate snack - 6 low-sodium whole grain crackers (28g) and 1 slice (19g) of low-fat prepackaged American cheese after dinner and avoided other food or drink containing calories in the evening. | Background diet and consumed 28g/d dry roasted, unsalted, skinless peanuts after dinner and avoided other food or drink containing calories in the evening. | SBP →, DBP →, TG →, TC →, LDL-C →, HDL-C →, Apo B ↑ | (+) |
| Sauder et al. (2014)^127^; USA | CR, 4 weeks per arm | 30 (15) | All participants: 56.1 ± 7.8   All participants: 31.2 ± 3.1   Adults (females are post-menopausal) with well-controlled type 2 diabetes and BMI of 18.5-45.0kg/m^2^ | Unmedicated | Mixed (Statin: 43.3%) | The AHATLC diet (26.9% energy from total fat, 6.7% energy from saturated fat, 186mg/d cholesterol) | The AHATLC diet (same as CG) and replaced low-fat or fat-free snacks (i.e., pretzels, string cheese, etc.) with roasted pistachios (1/2 unsalted are incorporated into entrees and 1/2 salted are eaten as snacks), providing 20% of daily energy (range: 59–128g) | SBP →, DBP → | (+) |
| Sauder et al. (2015)^120^; USA | CR, 4 weeks per arm | 30 (15) | All participants: 56.1 ± 7.8   All participants: 31.2 ± 3.1   Adults (females are post-menopausal) with well-controlled type 2 diabetes and BMI of 18.5-45.0kg/m^2^ | Unmedicated | Mixed (Statin: 43.3%) | The AHATLC diet (26.9% energy from total fat, 6.7% energy from saturated fat, 186mg/d cholesterol) | The AHATLC diet (same as CG) and replaced low-fat or fat-free snacks (i.e., pretzels, string cheese, etc.) with roasted pistachios (1/2 unsalted are incorporated into entrees and 1/2 salted are eaten as snacks), providing 20% of daily energy (range: 59–128g) | TG ↓, TC ↓, LDL-C →, HDL-C → | (∅) |
| Sheridan et al. (2007)^102^; USA | CR, 4 weeks per arm | 15 (4) | All participants: 60 ± 11.23   All participants: 27.7 ± 3.49   Participants with moderate hypercholesterolemia | Unmedicated | Unmedicated | Normal, regular diet | Normal, regular diet and substituted pistachio nuts (15% of the daily caloric intake, about 2 to 3 ounces per day) for normally consumed high-fat snacks or other fat calories. | SBP →, DBP →, TG →, TC →, LDL-C ↓, HDL-C ↑, VLDL-C →, Apo A1 →, Apo B → | (∅) |
| Tapsell et al. (2004)^124^; Australia | P, 6 months | IG: 17 (6); CG: 20 (10) | IG: 57.71 ± 8.97, CG: 59.30 ± 7.11   IG: 30.72 ± 3.85, CG: 30.16 ± 4.51   Participants diagnosed with type 2 diabetes for at least 1 year, and generally well | Unreported | Unreported | Modified low-fat diet | Modified low-fat diet as CG with 30g/d walnuts. | TG →, TC →, LDL-C →, HDL-C ↑ | (∅) |
| Tapsell et al. (2017)^86^; Australia | P, 12 months | IG: 126; CG: 124(only report 74% women for the whole trial) | All participants: median: 45 (IQR: 37–51)   All participants: median: 32 (IQR: 29–35)   Community-dwelling residents with a BMI of 25–40 kg/m^2^ | Mixed (All types: IG: 14%, 17/126; CG: 16%, 20/125) | Mixed (All types: IG: 6%, 7/126; CG: 8%, 10/125) | Receive an interdisciplinary model of care which aims at weight loss. In particular with the dietary aspects, an APD negotiated changes in specific food choices based on a diet history assessment and AGHE. | Receive an interdisciplinary model of care same as CG and substituted 30g/d walnuts in the diet. | SBP →, DBP →, TG →, TC →, LDL-C →, HDL-C → | (∅) |
| Tey et al. (2011)^64^; New Zealand | P, 12 weeks | IG: 32 (17); CG: 27 (17/29-randomised) | IG: 38.9 ± 14.3, CG: 36.1 ± 15.2   IG: 24.6 (2.8), CG: 22.9 (2.8)   Healthy males or females | Unreported | Unreported | Regular diet with no additional food | Regular diet and consumed 42g/d hazelnuts as a snack | TG →, TC →, LDL-C →, HDL-C → | (+) |
| Tey et al. (2013)^83^; New Zealand | P, 12 weeks | IG - 30g/d: 33 (20); IG - 60g/d: 37 (20); CG: 37 (21) | IG - 30g/d: 43.8 ± 13.5, IG- 60g/d: 42.8 ± 10.6, CG: 41.1 ± 13.1  IG - 30g/d: 30.7 ± 4.7, IG - 60g/d: 30.9 ± 6.0, CG: 30.4 ± 4.5   Adults with a BMI ≥25 kg/m^2^ | Unreported | Unreported | Usual diet without nuts | Usual diet. IG-30g/d: consumed 30g/d raw, unsalted Ennis hazelnuts **OR** IG-60g/d: consumed 60g/d hazelnuts | IG - 30g/d: SBP →, DBP →, TG →, TC →, LDL-C →, HDL-C →, Apo A1 →, Apo B →; IG - 60g/d: SBP →, DBP →, TG →, TC →, LDL-C →, HDL-C →, Apo A1 →, Apo B → | (+) |
| Tindall et al. (2019)^150^; USA^c^ | CR, 6 weeks per arm | 45 (20) | All participants: 43 ± 10   All participants: 30.3 ± 4.7   Men and women with overweight and obesity, who had LDL-C between the 50th and 90th percentiles from a nationally representative sample and/or elevated brachial SBP/DBP | Unmedicated | Unmedicated | Isocaloric weight maintenance diets (48% energy from carbohydrate, 17% from protein, 35% from fat, 7% from SFAs). | Isocaloric weight maintenance diets with similar carbohydrate, protein, and fat profiles as CG, included 18% of daily energy from walnuts as a snack (57–99g/d). | SBP →, DBP →, TG →, TC →, LDL-C →, HDL-C →, non-HDL-C → | (+) |
| Tindall et al. (2020)^151^; USA^c^ | CR, 6 weeks per arm | 34 (13) | All participants: 44 ± 10   All participants: 30.3 ± 4.7  30.1 ± 4.9 Men and women with overweight and obesity, who had LDL-C between the 50th and 90th percentiles from a nationally representative sample and/or elevated brachial SBP/DBP | Unmedicated | Unmedicated | Isocaloric weight maintenance diets (48% energy from carbohydrate, 17% from protein, 35% from fat, 7% from SFAs). | Isocaloric weight maintenance diets with similar carbohydrate, protein, and fat profiles as CG, included 18% of daily energy from walnuts as a snack (57–99g/d). | VLDL-C →, IDL-C →, Lp(a) → | (∅) |
| Toledo et al. (2013)^53^; Spain ^e^ | P, 4 years | IG: 2367 (1275); CG: 2441 (1424) | IG: 66.6 ± 6.1, CG: 66.9 ± 6.2   IG: 29.7 ± 3.8, CG: 29.9 ± 3.7   Community-dwelling men and women who fulfilled at least 1 of 2 criteria: type 2 diabetes or 3 or more CHD risk factors. | Mixed (All types: IG: 68.4%, 1648/2367; CG: 68.0%, 1660/2441) | Mixed (All types: IG: 44.0%,1041/2367; CG: 44.9%, 1095/2441) | Mediterranean-style diet with virgin olive oil (1L/week) | Mediterranean-style diet with mixed nut (30g/d, including 15g/d of walnut, 7.5g/d of hazelnut, and 7.5g/d of almond) | SBP (NR), DBP (NR) | (+) |
| Torabian et al. (2010)^143^; United States | CR, 6 months | 87 (49) | All participants: 54±10.2   All participants: 26.5 ± 3.3   Non-smoking men and women with normal to moderate high plasma TC | Unreported | Unmedicated | Habitual diet | Habitual diet with 28-64g/d walnut (12% of total energy intake) | TG ↓, TC ↓, LDL-C ↓, HDL-C → | (∅) |
| Wang et al. (2012)^139^; China | P, 12 weeks | IG - RSG: 27 (14/30-randomised); IG - HSG: 29 (18/30-randomised); CG: 30 (17) | IG - RSG: 51.89 ± 8.82, IG - HSG: 51.83 ± 9.37, CG: 50.66 ± 9.86  IG - RSG: 28.12 ± 3.22, IG - HSG: 28.01 ± 4.51, CG: 28.03 ± 4.35   Male and female who also met the metabolic syndrome standards for a Chinese population and without diabetes | Unreported | Unreported | Subjects were counselled on healthy eating at each visit according to the guidelines of the American Heart Association Step I diet and consumed no tree nuts. | Similar diet as CG. IG-RSG: consumed 42g/d pistachios as a snack in the afternoon **OR** IG-HSG: consumed 70g/d pistachios as a snack in the afternoon. | TG →, TC →, LDL-C → | (∅) |
| Wang et al. (2021)^138^; China | P, 12 weeks | IG: 113 (78); CG: 111 (78) | IG: 46.2 ± 9.9, CG: 46.2 ± 9.9   IG: 28.4 ± 3.4, CG: 27.6 ± 2.7   Participants at risk of or with metabolic syndromes | Unreported | Unmedicated | Background/ habitual diet and consumed 2 packets of isocaloric white rice snack bars per day, providing 1392kJ/d (approximately 20% of daily energy intake), 1 hour before lunch and before dinner each day. | Background/ habitual diet and consumed 2 packs of roasted lightly salted peanuts (28g, or 1 serving/packet; 56g in total) per day, providing 1392kJ/d (approximately 20% of daily energy intake), 1 hour before lunch and before dinner each day. | SBP →, DBP →, TG →, TC →, LDL-C →, HDL-C → | (+) |
| Wang et al. (2021)^92^; USA | P, 24 weeks | IG: 56 (39); CG: 39 (32) | IG: 48.3 ± 14.22, CG: 46.9 ± 10.62   IG: 31.1 ± 2.99, CG: 30.7 ± 2.50   Healthy free-living women and men with overweight/ obesity | Unreported | Unreported | Personalised hypocaloric (-500 kcal/day) meal plan with a daily snack of pretzels (isocaloric with 1.5 oz mixed nuts) for the first 12 weeks, followed by isocaloric meal plans aimed for weight maintenance for another 12 weeks. | Personalised hypocaloric (-500 kcal/day) meal plans with a daily snack of 1.5 oz. mixed nuts (almonds, cashews, hazelnuts, macadamia, pecans, pistachios, and walnuts) for the first 12 weeks, followed by isocaloric meal plans aimed for weight maintenance for another 12 weeks. | SBP →, DBP →, TG →, TC →, HDL-C → | (+) |
| West et al. (2012)^98^; USA^a^ | CR, 4 weeks per arm | 28 (18) | All participants: 48 ± 1.5   All participants: 26.8 ± 0.7   Healthy, non-smoking adult with elevated LDL-C (≥2.86 mmol/L) | Unmedicated | Unmedicated | Step I diet (cholesterol-lowering diet with an average intake of 2500kcal/d to maintain weight), consistent with food-based dietary recommendations | Step I diet. IG-1PD: consumed 1 serving of roasted and salted pistachio (10% of total energy, dose-ranging 32 - 63g/d) **OR** IG – 2PD: consumed 2 servings of roasted and salted pistachio (20% of total energy, dose ranging 63-126g/d) | IG-1PD: SBP →, DBP →; IG-2PD: SBP →, DBP → | (-) |
| Wien et al. (2003)^149^; USA | P, 24 weeks | IG: 32 (19); CG: 33 (18) | IG: 53 ± 2, CG: 57 ± 2   IG: 39 ± 1, CG: 37 ± 1   Free-living outpatients who entered into the 24-week Diabetes and Cardiovascular Risk Reduction Program (D & CVRRP) for medically supervised weight reduction and had a medical diagnosis that can be ameliorated or improved by weight reduction and BMI ≥25 kg/m^2^. | Mixed (NR) | Unmedicated | A formula-based LCD supplemented with self-selected complex carbohydrates (explicit instructions on how to self-select a combination of complex carbohydrates daily from a food list that was equivalent in calories to 84g of almonds were given) and 2 teaspoons of safflower oil per day. The two study groups had distinctly different levels of total fat (18% of energy in CG vs 39% of energy in IG) and MUFA (5% of energy in CG vs 25% of energy in IG); however, both groups were equally balanced on total calories, protein, cholesterol and saturated fat. | A similar formula-based LCD as CG supplemented with 84 g/day whole unblanched unsalted almonds consumed at the time of day most convenient to their lifestyle. | SBP ↓, DBP →, TG →, TC →, LDL-C →, HDL-C ↓ | (+) |
| Wien et al. (2010)^114^; USA | P, 16 weeks | IG: 32 (22); CG: 33 (26) | IG: 53 ± 9, CG: 54 ± 11   IG: 30 ± 5, CG: 29 ± 5   Free-living individuals with presence of prediabetes | Unreported | Mixed (All types: IG: 6%; CG: 6%) | The study dietitian prescribed an individualised ADA diet, contained 15%–20% protein, <10% saturated fat, 60%–70% carbohydrate and MUFA, and cholesterol <300 mg/day), participants were prescribed compensatory servings from the meat and fat exchange lists (equal to energy from the almonds). | An ADA diet similar to CG, with 20% of energy from raw or dry roasted almonds and avoided other tree nuts and peanuts. | SBP →, DBP →, TG →, TC →, LDL-C →, HDL-C → | (+) |
| Wu et al. (2010)^137^; China | P, 12 weeks | IG: 94 (41); CG: 95 (43) | IG: 48.2 ± 8.4, CG: 48.6 ± 8.0   IG: 25.7 ± 2.9, CG: 25.4 ± 2.4   Chinese men and women with metabolic syndromes | Mixed (All types: 41.5%, 39/94; CG: 33.7%, 32/95) | Mixed (All types: IG: 4.3%, 4/94; CG: 2.1%, 2/95) | Healthy lifestyle counselling based on the AHA guidelines | Healthy lifestyle counselling with 100g bread incorporated with 30g whole walnut | SBP (NR), DBP (NR), TG (NR), TC (NR), LDL-C (NR), HDL-C (NR), Apo A1 (NR), Apo B (NR) | (+) |
| Wu et al. (2014)^58^; Germany | CR, 8 weeks per arm | 40 (30) | All participants: 60 ± 6.3   All participants: 24.9 ± 3.8   Healthy Caucasian men and postmenopausal women | Unmedicated | Unmedicated | A nut-free Western-type diet consisting of 35% fat (15% saturated fat), 15% protein and 50% carbohydrates with no fish oil or vitamin E supplements | An isocaloric diet as CG with 43 g of shelled, prepackaged walnuts daily to replace 30 g of saturated fat with handouts to assist with integrating walnuts into the diets. | TG →, TC →, LDL-C →, HDL-C →, VLDL-C →, non-HDL-C ↓, Apo B ↓ | (+) |
| Yilmaz & Ozyildirim (2019)^144^; Turkey | P, 6 weeks | IG: 9 (9); CG: 10 (10) | IG: 52.0 ± 6.4, CG: 52.0 ± 3.5   IG: 35.7 ± 4.7, CG: 36.0 ± 6.1   Non-pregnant women who had no accompanying chronic disease and did not use medication due to hyperlipidemia with a BMI >30 kg/m², TC >200 mg/dL, LDL-C >130 mg/dL, fasting blood glucose <110 mg/dL and SBP/DBP <140/90 mmHg | Unreported | Unmedicated | A cardioprotective diet (estimated energy distribution: 50-60% carbohydrates, 15% proteins, and 25-35% fats) with no hazelnut and raisins | A cardioprotective diet with no raisins with 50 g/day hazelnut | SBP →, DBP →, TG →, TC →, LDL-C →, HDL-C → | (+) |
| Zambon et al. (2000)^107^; Spain | CR, 6 weeks per arm | 49 (23) | All participants: 56 ± 11   All participants: 27.0 ± 3.1   Free-living adult men and women with polygenic hypercholesterolemia; no evidence of alcohol, tobacco, or drug abuse; absence of diabetes mellitus and liver, kidney, thyroid, or other endocrine diseases | Mixed (All types: 16.4%) | Unmedicated | A cholesterol-lowering Mediterranean diet. Diets were individually prescribed and were based on estimated energy requirements - they were calculated in increments of 200kcal to cover the range from 1600 to 2200kcal. | A diet of similar energy and fat content compared with CG, except for the inclusion of raw, shelled walnuts (18% of the total energy and 35% of the total fat, ranged from 41-56g) replaced with olive oil and other fatty foods in CG. Walnuts were consumed as snacks or with meals in desserts or salads. | TG →, TC →, LDL-C →, HDL-C →, VLDL-C →, Apo A1 →, Apo B →, Lp(a) → | (∅) |
| Zibaeenezhad et al. (2005)^50^; Iran^#^ | P, 8 weeks | IG: 20 (NR); CG:23 (NR) | NR   NR   Hyperlipidaemic participants with either plasma TG > 350mg/dL (4mmol/L) or TC >250mg/dL (6.36mmol/L) | Unmedicated | Unmedicated | Habitual diet (devoid of walnuts) | Habitual diet with 20g/d Persian walnuts (Juglans regia L.) | TG →, TC ↓, LDL-C →, HDL-C ↑ | (-) |

**Abbreviations:** ACE, Angiotensin-converting enzyme; ADA, American Dietetic Association; AHA, American Heart Association; AHATLC, American heart association’s therapeutic lifestyle changes; AGHE, Australian Guide to Healthy Eating; ALD, ad libitum diet; AMDR, acceptable macronutrient distribution ranges; APD, accredited practising dietitian; Apo, apolipoprotein; BMI, body mass index, BP, blood pressure; CAD, Coronary Artery Disease; CG, control group; CHILD, cardiovascular health integrated lifestyle diet; CHO, carbohydrates, CR, cross over; CVD, cardiovascular disease; d, day; DBP, diastolic blood pressure; EER, estimated energy requirement; g, gram; HDL-C, high-density lipoprotein; IDL-C, intermediate-density lipoprotein; IG, intervention group; IQR, Interquartile range; kcal, Kilocalorie; kJ, Kilojoule; LCD, low-calorie diet, LDL-C, low-density lipoprotein - cholesterol; Lp, lipoprotein; mJ, Megajoule ; MUFA, monounsaturated fatty acid; NA, not applicable; NCEP, National Cholesterol Education Program; NM, not measured; NR, not reported; oz, ounce; P, parallel; PUFA, polyunsaturated fatty acids; QA, quality assessment, SBP, systolic blood pressure; TC, total cholesterol; TG, triglycerides; USA, United States of America; VLDL-C, very low-density lipoprotein; WC, waist circumference.

# Articles that were not included in the meta-analysis (n=9)

→ The results of the outcomes in the intervention (nut group) were not significantly different compared with the control; ↓ The results of the outcomes in the intervention (nut group) were significantly lower compared with the control; ↑ The results of the outcomes in the intervention (nut group) were significantly higher compared with the control; (NR) There were no between-group results, or group x time results available.

(+) Positive rating from the quality assessment; (∅) Neutral rating from the quality assessment; (-) Negative rating from the quality assessment

Articles with the same superscript letters were from the same studies.

# **Supplementary Table 4:** Certainty Assessment using GRADE framework

| **Certainty assessment** | | | | | | | **№ of patients** | | **Effect** | | **Certainty** | **Importance** |
| --- | --- | --- | --- | --- | --- | --- | --- | --- | --- | --- | --- | --- |
| **№ of studies** | **Study design** | **Risk of bias** | **Inconsistency** | **Indirectness** | **Imprecision** | **Other considerations** | **IG** | **CG** | **Relative (95% CI)** | **Absolute (95% CI)** |  |  |
| **SBP** | | | | | | | | | | | | |
| 60 | randomised trials | not serious | serious^a^ | not serious | not serious | none | 5447 | 5316 | - | MD **0.73 mmHg lower** (1.66 lower to 0.19 higher) | ⨁⨁⨁◯ Moderate^a^ | IMPORTANT |
| **DBP** | | | | | | | | | | | | |
| 59 | randomised trials | not serious | not serious | not serious | not serious | none | 5417 | 5286 | - | MD **0.32 mmHg lower** (0.69 lower to 0.05 higher) | ⨁⨁⨁⨁ High | IMPORTANT |
| **TG** | | | | | | | | | | | | |
| 100 | randomised trials | not serious | serious^b^ | not serious | not serious | none | 4844 | 4535 | - | MD **0.06 mmol/L lower** (0.08 lower to 0.04 lower) | ⨁⨁⨁◯ Moderate^b^ | IMPORTANT |
| **TC** | | | | | | | | | | | | |
| 101 | randomised trials | not serious | serious^c^ | not serious | not serious | none | 4552 | 4255 | - | MD **0.12 mmol/L lower** (0.16 lower to 0.08 lower) | ⨁⨁⨁◯ Moderate^c^ | IMPORTANT |
| **LDL-C** | | | | | | | | | | | | |
| 101 | randomised trials | not serious | serious^d^ | not serious | not serious | none | 4561 | 4277 | - | MD **0.11 mmol/L lower** (0.14 lower to 0.08 lower) | ⨁⨁⨁◯ Moderate^d^ | IMPORTANT |
| **HDL-C** | | | | | | | | | | | | |
| 101 | randomised trials | not serious | serious^e^ | not serious | not serious | none | 4848 | 4565 | - | MD **0.01 mmol/L higher** (0 to 0.03 higher) | ⨁⨁⨁◯ Moderate^e^ | IMPORTANT |
| **VLDL-C** | | | | | | | | | | | | |
| 24 | randomised trials | not serious | not serious | not serious | not serious | none | 1223 | 1178 | - | MD **0.04 mmol/L lower** (0.06 lower to 0.02 lower) | ⨁⨁⨁⨁ High | IMPORTANT |
| **IDL-C** | | | | | | | | | | | | |
| 5 | randomised trials | not serious | not serious | not serious | serious^f^ | none | 189 | 190 | - | MD **0.02 mmol/L lower** (0.04 lower to 0.01 higher) | ⨁⨁⨁◯ Moderate^f,^ | IMPORTANT |
| **Non-HDL-C** | | | | | | | | | | | | |
| 21 | randomised trials | serious^g^ | not serious | not serious | not serious | none | 1084 | 981 | - | MD **0.2 mmol/L lower** (0.27 lower to 0.13 lower) | ⨁⨁⨁◯ Moderate^g^ | IMPORTANT |
| **Apo A1** | | | | | | | | | | | | |
| 28 | randomised trials | not serious | not serious | not serious | not serious | none | 1223 | 1178 | - | MD **0.01 g/L lower** (0.02 lower to 0.01 higher) | ⨁⨁⨁⨁ High | IMPORTANT |
| **Apo B** | | | | | | | | | | | | |
| 34 | randomised trials | serious^h^ | not serious | not serious | not serious | none | 1663 | 1609 | - | MD **0.03 g/L lower** (0.05 lower to 0.02 lower) | ⨁⨁⨁◯ Moderate^h^ | IMPORTANT |
| **Lp(a)** | | | | | | | | | | | | |
| 12 | randomised trials | not serious | not serious | not serious | not serious | none | 806 | 816 | - | MD **0 g/L**  (0 to 0.01 higher) | ⨁⨁⨁⨁ High | IMPORTANT |

**Abbreviations:** Apo, apolipoprotein; CG, control group; CI, confidence interval; DBP, diastolic blood pressure; HDL-C, high-density lipoprotein - cholesterol; IDL-C, intermediate-density lipoprotein - cholesterol; IG, intervention group; LDL-C, low-density lipoprotein - cholesterol; Lp, lipoprotein; MD: mean difference; №, number; SBP, systolic blood pressure; TC, total cholesterol; TG, triglycerides; VLDL-C, very low-density lipoprotein – cholesterol

a. I^2^ value=76%, indicating high heterogeneity

b. I^2^ value=96%, indicating high heterogeneity

c. I^2^ value=89%, indicating high heterogeneity

d. I^2^ value=90%, indicating high heterogeneity

e. I^2^ value=98%, indicating high heterogeneity

f. Total number of participant=266, indicating a small sample size

g. I^2^ value=74%, indicating high heterogeneity

h. I^2^ value=77%, indicating high heterogeneity

# **Supplementary Table 5**: Results of subgroup analyses for systolic blood pressure

| **Subgroup analysis category** | **Subgroup** | **Number of analyses** | **Number of participants** | **Effect estimate (95% CI), I^2^ (%)** | **Test for subgroup differences** |
| --- | --- | --- | --- | --- | --- |
| Study design | Cross-over | 23 | 896 | -0.71 (-2.16, 0.74), 68% | Chi² = 0.00, df = 1 (P = 0.986), I^2^ = 0% |
|  | Parallel | 37 | 8941 | -0.69 (-1.82, 0.43), 76% |  |
| Duration | <12 weeks | 30 | 1274 | -0.29 (-1.43, 0.85), 57% | Chi² = 0.79, df = 1 (P = 0.374), I^2^ = 0% |
|  | ≥12 weeks | 30 | 8563 | -1.09 (-2.44, 0.25), 80% |  |
| Energy restriction | Yes | 9 | 757 | -0.83 (-3.71, 2.05), 73% | Chi² = 0.01, df = 1 (P = 0.919), I^2^ = 0% |
|  | No | 51 | 9080 | -0.68 (-1.58, 0.23), 72% |  |
| Health status | CAD | 2 | 158 | -1.05 (-4.20, 2.09), 0% | Chi² = 24.44, df = 8 (P = 0.002), I^2^ = 63% |
|  | High risk of CVD | 3 | 4942 | -2.42 (-3.21, -1.63), 0% |  |
|  | T2DM | 5 | 438 | -0.51 (-5.47, 4.44), 88% |  |
|  | Healthy | 5 | 906 | -0.74 (-3.15, 1.67), 68% |  |
|  | Hypercholesterolemia/ dyslipidemia | 9 | 292 | 0.35 (-1.32, 2.02), 0% |  |
|  | Metabolic syndrome | 6 | 671 | 0.67 (-0.93, 2.27), 2% |  |
|  | Multiple/ other | 6 | 274 | -3.94 (-8.61, 0.72), 78% |  |
|  | Overweight/ obesity | 17 | 1750 | 0.14 (-1.65, 1.93), 70% |  |
|  | Prediabetes | 7 | 406 | -2.66 (-4.08, -1.25), 25% |  |
| Nut type | Almond | 17 | 1556 | -1.47 (-3.14, 0.20), 78% | Chi² = 11.33, df = 7 (P = 0.133), I^2^ = 38% |
|  | Cashew | 2 | 312 | -3.41 (-6.12, -0.71), 0% |  |
|  | Hazelnut | 3 | 186 | 0.91 (-1.89, 3.70), 0% |  |
|  | Macadamia | 0 | 0 | NA |  |
|  | Mixed | 11 | 5334 | 0.78 (-1.08, 2.65), 54% |  |
|  | Peanut | 5 | 459 | -0.98 (-3.77, 1.82), 58% |  |
|  | Pecan | 3 | 171 | 0.15 (-3.11, 3.41), 0% |  |
|  | Pistachio | 7 | 309 | -2.61 (-5.08, -0.15), 61% |  |
|  | Walnut | 14 | 1550 | -0.08 (-2.08, 1.92), 71% |  |
| Nut dose | <30g | 3 | 223 | -0.64 (-2.87, 1.59), 0% | Chi² = 0.19, df = 2 (P = 0.910), I^2^ = 0% |
|  | 30-59g | 41 | 8673 | -0.54 (-1.66, 0.59), 80% |  |
|  | ≥60g | 18 | 1006 | -0.95 (-2.44, 0.54), 52% |  |

Abbreviations: CAD, coronary artery disease; CI, confidence interval; CVD, cardiovascular disease; df, degree of freedom; NA, not applicable

# **Supplementary Table 6**: Results of subgroup analyses for diastolic blood pressure

| **Subgroup analysis category** | **Subgroup** | **Number of analyses** | **Number of participants** | **Effect estimate (95% CI), I^2^ (%)** | **Test for subgroup differences** |
| --- | --- | --- | --- | --- | --- |
| Study design | Cross-over | 23 | 896 | -0.09 (-0.71, 0.55), 23% | Chi² = 0.91, df = 1 (P = 0.339), I^2^ = 0% |
|  | Parallel | 36 | 8881 | -0.45 (-0.87, -0.03), 19% |  |
| Duration | <12 weeks | 29 | 1214 | -0.06 (-0.69, 0.56), 30% | Chi² = 0.60, df = 1 (P = 0.438), I^2^ = 0% |
|  | ≥12 weeks | 30 | 8563 | -0.36 (-0.77, 0.05), 10% |  |
| Energy restriction | Yes | 9 | 757 | -0.87 (-2.37, 0.63), 53% | Chi² = 0.67, df = 1 (P = 0.413), I^2^ = 0% |
|  | No | 50 | 9020 | -0.23 (-0.59, 0.14), 19% |  |
| Health status | CAD | 2 | 158 | -0.31 (-2.65, 2.04), 0% | Chi² = 12.18, df = 8 (P = 0.143), I^2^ = 34% |
|  | High risk of CVD | 3 | 4942 | -0.10 (-0.58, 0.37), 0% |  |
|  | T2DM | 5 | 438 | -0.15 (-2.60, 2.30), 78% |  |
|  | Healthy | 5 | 906 | -0.93 (-1.37, -0.50), 0% |  |
|  | Hypercholesterolemia/ dyslipidemia | 9 | 292 | -0.03 (-1.11, 1.05), 0% |  |
|  | Metabolic syndrome | 5 | 611 | 0.81 (-0.36, 1.97), 0% |  |
|  | Multiple/ other | 6 | 274 | -0.61 (-1.95, 0.74), 0% |  |
|  | Overweight/ obesity | 17 | 1750 | -0.63 (-1.53, 0.28), 36% |  |
|  | Prediabetes | 7 | 406 | -0.32 (-1.07, 0.44), 0% |  |
| Nut type | Almond | 17 | 1556 | -0.99 (-1.37, -0.61), 0% | Chi² = 18.81, df = 7 (P = 0.009), I^2^ = 63% |
|  | Cashew | 2 | 312 | -1.44 (-3.14, 0.25), 0% |  |
|  | Hazelnut | 3 | 186 | 1.30 (-0.83, 3.43), 0% |  |
|  | Macadamia | 0 | 0 | NA |  |
|  | Mixed | 10 | 5274 | -0.07 (-0.52, 0.38), 0% |  |
|  | Peanut | 5 | 459 | -0.40 (-1.58, 0.77), 9% |  |
|  | Pecan | 3 | 171 | 1.27 (-1.40, 3.95), 27% |  |
|  | Pistachio | 7 | 309 | -0.10 (-0.98, 0.77), 0% |  |
|  | Walnut | 14 | 1550 | 0.28 (-0.67, 1.23), 40% |  |
| Nut dose | <30g | 3 | 223 | 0.58 (-1.15, 2.31), 0% | Chi² = 1.15, df = 2 (P = 0.562), I^2^ = 0% |
|  | 30-59g | 40 | 8613 | -0.28 (-0.77, 0.21), 47% |  |
|  | ≥60g | 18 | 1006 | -0.44 (-1.11, 0.24), 0% |  |

Abbreviations: CAD, coronary artery disease; CI, confidence interval; CVD, cardiovascular disease; df, degree of freedom; NA, not applicable

# **Supplementary Table 7**: Results of subgroup analyses for triglycerides

| **Subgroup analysis category** | **Subgroup** | **Number of analyses** | **Number of participants** | **Effect estimate (95% CI), I^2^ (%)** | **Test for subgroup differences** |
| --- | --- | --- | --- | --- | --- |
| Study design | Cross-over | 39 | 1634 | -0.08 (-0.10, -0.05), 88% | Chi² = 2.44, df = 1 (P = 0.118), I^2^ = 59% |
|  | Parallel | 61 | 6081 | -0.05 (-0.08, -0.02), 59% |  |
| Duration | <12 weeks | 56 | 2347 | -0.08 (-0.10, -0.05), 54% | Chi² = 2.01, df = 1 (P = 0.156), I^2^ = 50% |
|  | ≥12 weeks | 44 | 5368 | -0.05 (-0.08, -0.02), 92% |  |
| Energy restriction | Yes | 13 | 1097 | -0.11 (-0.23, 0.02), 73% | Chi² = 0.45, df = 1 (P = 0.502), I^2^ = 0% |
|  | No | 87 | 6618 | -0.06 (-0.08, -0.04), 96% |  |
| Health status | CAD | 3 | 293 | -0.14 (-0.28, 0.00), 14% | Chi² = 52.28, df = 8 (P < 0.001), I^2^ = 85% |
|  | High risk of CVD | 3 | 334 | 0.00 (-0.00, 0.00), 0% |  |
|  | T2DM | 7 | 522 | -0.10 (-0.22, 0.02), 47% |  |
|  | Healthy | 19 | 1692 | -0.09 (-0.12, -0.05), 80% |  |
|  | Hypercholesterolemia/ dyslipidemia | 16 | 548 | -0.06 (-0.11, -0.01), 0% |  |
|  | Metabolic syndrome | 8 | 825 | -0.14 (-0.22, -0.05), 14% |  |
|  | Multiple/ other | 13 | 626 | -0.04 (-0.09, 0.01), 57% |  |
|  | Overweight/ obesity | 23 | 2291 | -0.05 (-0.09, 0.00), 52% |  |
|  | Prediabetes | 8 | 584 | -0.06 (-0.10, -0.02), 0% |  |
| Nut type | Almond | 28 | 2527 | -0.06 (-0.09, -0.02), 43% | Chi² = 91.52, df = 8 (P < 0.001), I^2^ = 91% |
|  | Cashew | 4 | 397 | -0.03 (-0.14, 0.09), 8% |  |
|  | Hazelnut | 4 | 234 | -0.13 (-0.36, 0.09), 66% |  |
|  | Macadamia | 2 | 49 | -0.05 (-0.22, 0.13), 47% |  |
|  | Mixed | 13 | 810 | -0.00 (-0.00, 0.00), 0% |  |
|  | Peanut | 6 | 478 | -0.10 (-0.18, -0.03), 0% |  |
|  | Pecan | 6 | 293 | -0.17 (-0.29, -0.04), 8% |  |
|  | Pistachio | 10 | 515 | -0.13 (-0.16, -0.10), 0% |  |
|  | Walnut | 29 | 2452 | -0.06 (-0.09, -0.03), 94% |  |
| Nut dose | <30g | 7 | 351 | -0.14 (-0.22, -0.06), 18% | Chi² = 22.96, df = 2 (P < 0.001), I^2^ = 91% |
|  | 30-59g | 67 | 6098 | -0.05 (-0.07, -0.03), 82% |  |
|  | ≥60g | 31 | 1396 | -0.11 (-0.13, -0.09), 7% |  |

Abbreviations: CAD, coronary artery disease; CI, confidence interval; CVD, cardiovascular disease; df, degree of freedom; NA, not applicable

# **Supplementary Table 8**: Results of subgroup analyses for total cholesterol

| **Subgroup analysis category** | **Subgroup** | **Number of analyses** | **Number of participants** | **Effect estimate (95% CI), I^2^ (%)** | **Test for subgroup differences** |
| --- | --- | --- | --- | --- | --- |
| Study design | Cross-over | 41 | 2355 | -0.14 (-0.20, -0.09), 90% | Chi² = 0.58, df = 1 (P = 0.445), I^2^ = 0% |
|  | Parallel | 60 | 4728 | -0.11 (-0.17, -0.05), 84% |  |
| Duration | <12 weeks | 58 | 1746 | -0.14 (-0.20, -0.08), 90% | Chi² = 0.76, df = 1 (P = 0.382), I^2^ = 0% |
|  | ≥12 weeks | 43 | 5337 | -0.10 (-0.16, -0.05), 76% |  |
| Energy restriction | Yes | 12 | 971 | -0.09 (-0.34, 0.16), 90% | Chi² = 0.09, df = 1 (P = 0.758), I^2^ = 0% |
|  | No | 89 | 6112 | -0.13 (-0.17, -0.09), 87% |  |
| Health status | CAD | 3 | 293 | -0.08 (-0.21, 0.05), 0% | Chi² = 5.37, df = 8 (P = 0.718), I^2^ = 0% |
|  | High risk of CVD | 3 | 334 | 0.07 (-0.29, 0.42), 89% |  |
|  | T2DM | 9 | 582 | -0.07 (-0.15, 0.02), 45% |  |
|  | Healthy | 19 | 1126 | -0.17 (-0.28, -0.07), 95% |  |
|  | Hypercholesterolemia/ dyslipidemia | 16 | 548 | -0.13 (-0.27, -0.00), 83% |  |
|  | Metabolic syndrome | 8 | 825 | -0.10 (-0.20, 0.01), 43% |  |
|  | Multiple/ other | 13 | 626 | -0.08 (-0.17, 0.00), 85% |  |
|  | Overweight/ obesity | 22 | 2165 | -0.16 (-0.26, -0.06), 81% |  |
|  | Prediabetes | 8 | 584 | -0.15 (-0.28, -0.03), 77% |  |
| Nut type | Almond | 30 | 2587 | -0.16 (-0.23, -0.09), 79% | Chi² = 15.58, df = 8 (P = 0.049), I^2^ = 49% |
|  | Cashew | 4 | 397 | -0.05 (-0.23, 0.13), 61% |  |
|  | Hazelnut | 5 | 293 | -0.11 (-0.23, 0.00), 0% |  |
|  | Macadamia | 2 | 49 | -0.06 (-0.26, 0.14), 0% |  |
|  | Mixed | 13 | 810 | 0.10 (-0.06, 0.26), 77% |  |
|  | Peanut | 6 | 478 | -0.08 (-0.45, 0.29), 94% |  |
|  | Pecan | 6 | 293 | -0.25 (-0.38, -0.11), 20% |  |
|  | Pistachio | 10 | 515 | -0.16 (-0.31, -0.01), 92% |  |
|  | Walnut | 27 | 1701 | -0.18 (-0.23, -0.13), 81% |  |
| Nut dose | <30g | 7 | 351 | -0.14 (-0.30, 0.03), 63% | Chi² = 3.74, df = 2 (P = 0.154), I^2^ = 47% |
|  | 30-59 | 66 | 5406 | -0.10 (-0.16, -0.05), 91% |  |
|  | ≥60g | 33 | 1456 | -0.19 (-0.26, -0.12), 78% |  |

Abbreviations: CAD, coronary artery disease; CI, confidence interval; CVD, cardiovascular disease; df, degree of freedom; NA, not applicable

# **Supplementary Table 9**: Results of subgroup analyses for low-density lipoprotein-cholesterol

| **Subgroup analysis category** | **Subgroup** | **Number of analyses** | **Number of participants** | **Effect estimate (95% CI), I^2^ (%)** | **Test for subgroup differences** |
| --- | --- | --- | --- | --- | --- |
| Study design | Cross-over | 41 | 1694 | -0.14 (-0.18, -0.09), 89% | Chi² = 4.12, df = 1 (P = 0.042), I^2^ = 76% |
|  | Parallel | 60 | 5420 | -0.08 (-0.11, -0.04), 84% |  |
| Duration | <12 weeks | 58 | 2407 | -0.13 (-0.17, -0.08), 91% | Chi² = 3.36, df = 1 (P = 0.067), I^2^ = 66% |
|  | ≥12 weeks | 43 | 4707 | -0.08 (-0.11, -0.04), 66% |  |
| Energy restriction | Yes | 12 | 1002 | -0.04 (-0.11, 0.03), 42% | Chi² = 3.90, df = 1 (P = 0.042), I^2^ = 70% |
|  | No | 89 | 6112 | -0.12 (-0.15, -0.08), 89% |  |
| Health status | CAD | 3 | 293 | -0.10 (-0.39, 0.18), 85% | Chi² = 13.88, df = 8 (P = 0.085), I^2^ = 42% |
|  | High risk of CVD | 3 | 334 | -0.09 (-0.21, 0.02), 38% |  |
|  | T2DM | 9 | 582 | -0.03 (-0.08, 0.02), 0% |  |
|  | Healthy | 19 | 1126 | -0.14 (-0.22, -0.07), 95% |  |
|  | Hypercholesterolemia/ dyslipidemia | 16 | 548 | -0.18 (-0.27, -0.10), 74% |  |
|  | Metabolic syndrome | 8 | 825 | -0.05 (-0.14, 0.04), 31% |  |
|  | Multiple/ other | 13 | 626 | -0.09 (-0.16, -0.02), 87% |  |
|  | Overweight/ obesity | 22 | 2196 | -0.10 (-0.16, -0.05), 66% |  |
|  | Prediabetes | 8 | 584 | -0.09 (-0.19, 0.01), 73% |  |
| Nut type | Almond | 30 | 2587 | -0.11 (-0.15, -0.06), 82% | Chi² = 16.49, df = 8 (P = 0.036), I^2^ = 51% |
|  | Cashew | 4 | 397 | -0.13 (-0.38, 0.12), 88% |  |
|  | Hazelnut | 5 | 293 | -0.10 (-0.21, 0.01), 0% |  |
|  | Macadamia | 2 | 49 | -0.08 (-0.26, 0.11), 0% |  |
|  | Mixed | 12 | 715 | 0.02 (-0.06, 0.10), 32% |  |
|  | Peanut | 6 | 478 | 0.02 (-0.21, 0.25), 85% |  |
|  | Pecan | 6 | 293 | -0.26 (-0.44, -0.08), 59% |  |
|  | Pistachio | 10 | 515 | -0.12 (-0.22, -0.03), 85% |  |
|  | Walnut | 28 | 1827 | -0.15 (-0.19, -0.10), 82% |  |
| Nut dose | <30g | 7 | 351 | -0.09 (-0.22, 0.05), 62% | Chi² = 0.92, df = 2 (P = 0.632), I^2^ = 0% |
|  | 30-59 | 66 | 5437 | -0.11 (-0.14, -0.07), 88% |  |
|  | ≥60g | 33 | 1456 | -0.14 (-0.19, -0.08), 82% |  |

Abbreviations: CAD, coronary artery disease; CI, confidence interval; CVD, cardiovascular disease; df, degree of freedom; NA, not applicable

# **Supplementary Table 10**: Results of subgroup analyses for high-density lipoprotein-cholesterol

| **Subgroup analysis category** | **Subgroup** | **Number of analyses** | **Number of participants** | **Effect estimate (95% CI), I^2^ (%)** | **Test for subgroup differences** |
| --- | --- | --- | --- | --- | --- |
| Study design | Cross-over | 41 | 1694 | 0.00 (-0.01, 0.01), 96% | Chi² = 2.73, df = 1 (P = 0.099), I^2^ = 63% |
|  | Parallel | 60 | 5995 | 0.02 (-0.00, 0.04), 95% |  |
| Duration | <12 weeks | 58 | 2407 | 0.02 (-0.00, 0.04), 98% | Chi² = 0.41, df = 1 (P = 0.521), I^2^ = 0% |
|  | ≥12 weeks | 43 | 5282 | 0.01 (-0.01, 0.03), 95% |  |
| Energy restriction | Yes | 13 | 1097 | 0.02 (-0.01, 0.04), 35% | Chi² = 0.17, df = 1 (P = 0.678), I^2^ = 0% |
|  | No | 88 | 6592 | 0.01 (-0.00, 0.03), 98% |  |
| Health status | CAD | 3 | 293 | 0.03 (-0.07, 0.13), 76% | Chi² = 21.97, df = 8 (P = 0.005), I^2^ = 64% |
|  | High risk of CVD | 3 | 334 | -0.03 (-0.10, 0.03), 44% |  |
|  | T2DM | 9 | 582 | -0.00 (-0.03, 0.03), 92% |  |
|  | Healthy | 19 | 1692 | 0.02 (-0.02, 0.07), 99% |  |
|  | Hypercholesterolemia/ dyslipidemia | 16 | 548 | 0.04 (0.01, 0.06), 51% |  |
|  | Metabolic syndrome | 7 | 739 | 0.01 (-0.03, 0.06), 81% |  |
|  | Multiple/ other | 13 | 626 | -0.00 (-0.04, 0.03), 97% |  |
|  | Overweight/ obesity | 23 | 2291 | 0.02 (-0.01, 0.04), 93% |  |
|  | Prediabetes | 8 | 584 | -0.02 (-0.03, -0.01), 14% |  |
| Nut type | Almond | 30 | 2587 | 0.00 (-0.03, 0.03), 96% | Chi² = 6.34, df = 8 (P = 0.609), I^2^ = 0% |
|  | Cashew | 4 | 397 | 0.01 (-0.05, 0.06), 52% |  |
|  | Hazelnut | 4 | 234 | 0.04 (-0.01, 0.08), 0% |  |
|  | Macadamia | 2 | 49 | 0.04 (-0.06, 0.15), 87% |  |
|  | Mixed | 13 | 810 | 0.02 (-0.01, 0.04), 18% |  |
|  | Peanut | 6 | 478 | 0.01 (-0.02, 0.03), 0% |  |
|  | Pecan | 6 | 293 | 0.02 (-0.04, 0.13), 35% |  |
|  | Pistachio | 9 | 429 | 0.06 (-0.00, 0.13), 99% |  |
|  | Walnut | 29 | 2452 | 0.00 (-0.02, 0.02), 97% |  |
| Nut dose | <30g | 66 | 351 | 0.00 (-0.03, 0.03), 22% | Chi² = 0.36, df = 2 (P = 0.835), I^2^ = 0% |
|  | 30-59 | 7 | 6041 | 0.01 (-0.00, 0.03), 97% |  |
|  | ≥60g | 32 | 1397 | 0.01 (-0.02, 0.04), 98% |  |

Abbreviations: CAD, coronary artery disease; CI, confidence interval; CVD, cardiovascular disease; df, degree of freedom; NA, not applicable

# **Supplementary Table 11**: Results of subgroup analyses for very-low-density lipoprotein-cholesterol

| **Subgroup analysis category** | **Subgroup** | **Number of analyses** | **Number of participants** | **Effect estimate (95% CI), I^2^ (%)** | **Test for subgroup differences** |
| --- | --- | --- | --- | --- | --- |
| Study design | Cross-over | 11 | 549 | -0.04 (-0.06, -0.02), 52% | Chi² = 0.38, df = 1 (P = 0.535), I^2^ = 0% |
|  | Parallel | 13 | 1243 | -0.05 (-0.09, -0.02), 37% |  |
| Duration | <12 weeks | 16 | 803 | -0.04 (-0.06, -0.02), 46% | Chi² = 0.08, df = 1 (P = 0.772), I^2^ = 0% |
|  | ≥12 weeks | 8 | 989 | -0.05 (-0.08, -0.01), 39% |  |
| Energy restriction | Yes | 4 | 241 | -0.07 (-0.14, 0.00), 0% | Chi² = 0.61, df = 1 (P = 0.434), I^2^ = 0% |
|  | No | 20 | 1551 | -0.04 (-0.06, -0.02), 47% |  |
| Health status | CAD | 1 | 113 | -0.06 (-0.39, 0.27), NA | Chi² = 12.93, df = 7 (P = 0.074), I^2^ = 46% |
|  | High risk of CVD | 2 | 134 | -0.00 (-0.06, 0.05), 0% |  |
|  | T2DM | 1 | 269 | 0.02 (-0.05, 0.08), NA |  |
|  | Healthy | 4 | 389 | -0.08 (-0.10, -0.05), 0% |  |
|  | Hypercholesterolemia/ dyslipidemia | 6 | 197 | -0.06 (-0.09, -0.03), 0% |  |
|  | Metabolic syndrome | 0 | 0 | NA |  |
|  | Multiple/ other | 2 | 82 | -0.03 (-0.09, 0.04), 79% |  |
|  | Overweight/ obesity | 5 | 269 | -0.02 (-0.07, 0.02), 16% |  |
|  | Prediabetes | 3 | 339 | -0.06 (-0.11, -0.00), 71% |  |
| Nut type | Almond | 10 | 898 | -0.05 (-0.08, -0.02), 45% | Chi² = 5.81, df = 6 (P = 0.445), I^2^ = 0% |
|  | Cashew | 1 | 269 | 0.02 (-0.05, 0.08), NA |  |
|  | Hazelnut | 0 | 0 | NA |  |
|  | Macadamia | 0 | 0 | NA |  |
|  | Mixed | 1 | 29 | -0.04 (-0.22, 0.13), NA |  |
|  | Peanut | 2 | 89 | -0.13 (-0.26, -0.01), 0% |  |
|  | Pecan | 1 | 26 | -0.02 (-0.11, 0.08), NA |  |
|  | Pistachio | 3 | 97 | -0.05 (-0.08, -0.02), 3% |  |
|  | Walnut | 6 | 384 | -0.04 (-0.08, 0.01), 63% |  |
| Nut dose | <30g | 19 | 113 | -0.06 (-0.39, 0.27), NA | Chi² = 0.24, df = 2 (P = 0.886), I^2^ = 0% |
|  | 30-59 | 1 | 1402 | -0.04 (-0.06, -0.03), 23% |  |
|  | ≥60g | 6 | 322 | -0.06 (-0.11, -0.01), 74% |  |

Abbreviations: CAD, coronary artery disease; CI, confidence interval; CVD, cardiovascular disease; df, degree of freedom; NA, not applicable

# **Supplementary Table 12**: Results of subgroup analyses for non-high-density lipoprotein-cholesterol

| **Subgroup analysis category** | **Subgroup** | **Number of analyses** | **Number of participants** | **Effect estimate (95% CI), I^2^ (%)** | **Test for subgroup differences** |
| --- | --- | --- | --- | --- | --- |
| Study design | Cross-over | 9 | 555 | -0.23 (-0.33, -0.12), 89% | Chi² = 1.08, df = 1 (P = 0.298), I^2^ = 7% |
|  | Parallel | 12 | 925 | -0.16 (-0.23, -0.09), 0% |  |
| Duration | <12 weeks | 17 | 1020 | -0.21 (-0.29, 0.13), 79% | Chi² = 0.27, df = 1 (P = 0.603), I^2^ = 0% |
|  | ≥12 weeks | 4 | 460 | -0.17 (-0.28, -0.07), 0% |  |
| Energy restriction | Yes | 2 | 96 | 0.03 (-0.14, 0.21), 0% | Chi² = 7.36, df = 1 (P = 0.007), I^2^ = 86% |
|  | No | 19 | 1384 | -0.22 (-0.29, -0.16), 71% |  |
| Health status | CAD | 1 | 135 | -0.18 (-0.48, 0.12), NA | Chi² = 43.79, df = 8 (P < 0.001), I^2^ = 82% |
|  | High risk of CVD | 2 | 134 | -0.10 (-0.35, 0.15), 65% |  |
|  | T2DM | 1 | 71 | -0.35 (-0.88, 0.18), NA |  |
|  | Healthy | 5 | 542 | -0.18 (-0.22, -0.15), 0% |  |
|  | Hypercholesterolemia/ dyslipidemia | 4 | 178 | -0.46 (-0.54, -0.37), 0% |  |
|  | Metabolic syndrome | 1 | 60 | -0.24 (-0.49, 0.01), NA |  |
|  | Multiple/ other | 3 | 160 | -0.09 (-0.21, 0.04), 41% |  |
|  | Overweight/ obesity | 3 | 167 | -0.24 (-0.32, -0.16), 0% |  |
|  | Prediabetes | 1 | 33 | 0.04 (-0.19, 0.26), NA |  |
| Nut type | Almond | 8 | 603 | -0.19 (-0.25, -0.14), 0% | Chi² = 39.31, df = 6 (P < 0.001), I^2^ = 85% |
|  | Cashew | 1 | 42 | -0.48 (-0.62, -0.34), NA |  |
|  | Hazelnut | 1 | 60 | -0.06 (-0.75, 0.64), NA |  |
|  | Macadamia | 0 | 0 | NA |  |
|  | Mixed | 4 | 227 | -0.08 (-0.25, 0.09), 28% |  |
|  | Peanut | 0 | 0 | NA |  |
|  | Pecan | 3 | 231 | -0.27 (-0.51, -0.03), 0% |  |
|  | Pistachio | 1 | 28 | -0.47 (-0.59, -0.35), NA |  |
|  | Walnut | 3 | 289 | -0.17 (-0.22, -0.12), 25% |  |
| Nut dose | <30g | 1 | 60 | -0.06 (-0.75, 0.64), NA | Chi² = 0.34, df = 2 (P = 0.846), I^2^ = 0% |
|  | 30-59 | 13 | 1022 | -0.22 (-0.28, -0.15), 73% |  |
|  | ≥60g | 8 | 426 | -0.25 (-0.43, 0.07), NA |  |

Abbreviations: CAD, coronary artery disease; CI, confidence interval; CVD, cardiovascular disease; df, degree of freedom; NA, not applicable

# **Supplementary Table 13**: Results of subgroup analyses for apolipoprotein A1

| **Subgroup analysis category** | **Subgroup** | **Number of analyses** | **Number of participants** | **Effect estimate (95% CI), I^2^ (%)** | **Test for subgroup differences** |
| --- | --- | --- | --- | --- | --- |
| Study design | Cross-over | 17 | 550 | -0.01 (-0.02, 0.01), 36% | Chi² = 0.61, df = 1 (P = 0.435), I^2^ = 0% |
|  | Parallel | 11 | 1301 | 0.00 (-0.02, 0.03), 12% |  |
| Duration | <12 weeks | 21 | 781 | -0.01 (-0.02, 0.01), 38% | Chi² = 0.10, df = 1 (P = 0.746), I^2^ = 0% |
|  | ≥12 weeks | 7 | 1070 | -0.00 (-0.03, 0.03), 1% |  |
| Energy restriction | Yes | 2 | 96 | 0.05 (-0.00, 0.10), 0% | Chi² = 4.10, df = 1 (P = 0.043), I^2^ = 76% |
|  | No | 26 | 1755 | -0.01 (-0.02, 0.00), 25% |  |
| Health status | CAD | 1 | 45 | -0.03 (-0.08, 0.03), NA | Chi² = 10.40, df = 8 (P = 0.238), I^2^ = 23% |
|  | High risk of CVD | 3 | 334 | 0.00 (-0.03, 0.03), 0% |  |
|  | T2DM | 1 | 71 | -0.03 (-0.12, 0.06), NA |  |
|  | Healthy | 5 | 217 | -0.03 (-0.05, -0.00), 0% |  |
|  | Hypercholesterolemia/ dyslipidemia | 9 | 248 | -0.00 (-0.02, 0.02), 18% |  |
|  | Metabolic syndrome | 1 | 189 | 0.02 (-0.06, 0.10), NA |  |
|  | Multiple/ other | 3 | 140 | 0.02 (-0.02, 0.05), 82% |  |
|  | Overweight/ obesity | 4 | 574 | -0.02 (-0.05, -0.00), 0% |  |
|  | Prediabetes | 1 | 33 | 0.04 (-0.02, 0.10), NA |  |
| Nut type | Almond | 11 | 870 | -0.01 (-0.03, 0.01), 36% | Chi² = 8.00, df = 4 (P = 0.092), I^2^ = 50% |
|  | Cashew | 0 | 0 | NA |  |
|  | Hazelnut | 1 | 107 | 0.04 (-0.06, 0.14), NA |  |
|  | Macadamia | 0 | 0 | NA |  |
|  | Mixed | 6 | 425 | 0.02 (-0.01, 0.05), 0% |  |
|  | Peanut | 0 | 0 | NA |  |
|  | Pecan | 0 | 0 | NA |  |
|  | Pistachio | 3 | 85 | 0.01 (-0.02, 0.04), 0% |  |
|  | Walnut | 8 | 382 | -0.01 (-0.02, -0.00), 0% |  |
| Nut dose | <30g | 1 | 20 | -0.03 (0.06, 0.00), NA | Chi² = 3.49, df = 2 (P = 0.175), I^2^ = 43% |
|  | 30-59 | 19 | 1444 | -0.00 (-0.02, 0.01), 37% |  |
|  | ≥60g | 11 | 470 | 0.01 (-0.01, 0.02), 0% |  |

Abbreviations: CAD, coronary artery disease; CI, confidence interval; CVD, cardiovascular disease; df, degree of freedom; NA, not applicable

# **Supplementary Table 14**: Results of subgroup analyses for apolipoprotein B

| **Subgroup analysis category** | **Subgroup** | **Number of analyses** | **Number of participants** | **Effect estimate (95% CI), I^2^ (%)** | **Test for subgroup differences** |
| --- | --- | --- | --- | --- | --- |
| Study design | Cross-over | 21 | 928 | -0.04 (-0.06, -0.02), 84% | Chi² = 5.78, df = 1 (P = 0.016), I^2^ = 83% |
|  | Parallel | 13 | 1416 | -0.01 (-0.03, 0.01), 0% |  |
| Duration | <12 weeks | 25 | 1104 | -0.04 (-0.06, -0.03), 79% | Chi² =10.08, df = 1 (P = 0.001), I^2^ = 90% |
|  | ≥12 weeks | 9 | 1240 | -0.00 (-0.02, 0.02), 0% |  |
| Energy restriction | Yes | 1 | 29 | -0.02 (-0.06, 0.03), NA | Chi² = 0.46, df = 1 (P = 0.497), I^2^ = 0% |
|  | No | 33 | 2288 | -0.03 (-0.05, -0.02), 77% |  |
| Health status | CAD | 1 | 45 | 0.05 (0.01, 0.09), NA | Chi² = 53.38, df = 8 (P < 0.001), I^2^ = 85% |
|  | High risk of CVD | 3 | 334 | -0.01 (-0.03, 0.02), 0% |  |
|  | T2DM | 1 | 71 | -0.09 (-0.20, 0.02), NA |  |
|  | Healthy | 8 | 505 | -0.05 (-0.06, -0.05), 0% |  |
|  | Hypercholesterolemia/ dyslipidemia | 9 | 248 | -0.05 (-0.07, -0.04), 0% |  |
|  | Metabolic syndrome | 2 | 273 | -0.00 (-0.03, 0.03), 0% |  |
|  | Multiple/ other | 3 | 159 | -0.05 (-0.10, -0.01), 81% |  |
|  | Overweight/ obesity | 5 | 626 | -0.04 (-0.06, -0.02), 0% |  |
|  | Prediabetes | 2 | 83 | 0.03 (-0.04, 0.09), 63% |  |
| Nut type | Almond | 11 | 870 | -0.02 (-0.05, -0.00), 62% | Chi² = 33.39, df = 6 (P < 0.001), I^2^ = 82% |
|  | Cashew | 0 | 0 | NA |  |
|  | Hazelnut | 1 | 107 | -0.02 (-0.11, 0.08), NA |  |
|  | Macadamia | 0 | 0 | NA |  |
|  | Mixed | 6 | 444 | -0.01 (-0.03, 0.02), 0% |  |
|  | Peanut | 1 | 50 | 0.06 (0.02, 0.10), NA |  |
|  | Pecan | 2 | 96 | -0.10 (-0.19, -0.01), 0% |  |
|  | Pistachio | 3 | 85 | -0.05 (-0.08, -0.03), 0% |  |
|  | Walnut | 11 | 710 | -0.05 (-0.07, -0.04), 77% |  |
| Nut dose | <30g | 2 | 70 | -0.01 (-0.15, 0.12), 97% | Chi² = 0.15, df = 2 (P = 0.926), I^2^ = 0% |
|  | 30-59 | 22 | 1791 | -0.04 (-0.05, -0.02), 67% |  |
|  | ≥60g | 13 | 566 | -0.04 (-0.06, -0.01), 55% |  |

Abbreviations: CAD, coronary artery disease; CI, confidence interval; CVD, cardiovascular disease; df, degree of freedom; NA, not applicable

# **Supplementary Table 15** Results of subgroup analyses for lipoprotein(a)

| **Subgroup analysis category** | **Subgroup** | **Number of analyses** | **Number of participants** | **Effect estimate (95% CI), I^2^ (%)** | **Test for subgroup differences** |
| --- | --- | --- | --- | --- | --- |
| Study design | Cross-over | 8 | 429 | 0.00 (0.00, 0.01), 0% | Chi² = 0.04, df = 1 (P = 0.844), I^2^ = 0% |
|  | Parallel | 4 | 764 | 0.01 (-0.02, 0.03), 0% |  |
| Duration | <12 weeks | 11 | 573 | 0.00 (0.00, 0.00), 0% | Chi² = 0.51, df = 1 (P = 0.475), I^2^ = 0% |
|  | ≥12 weeks | 1 | 620 | 0.02 (-0.02, 0.05), NA |  |
| Energy restriction | Yes | 0 | 0 | NA | NA |
|  | No | 12 | 1193 | 0.00 (0.00, 0.01), 0% |  |
| Health status | CAD | 0 | 0 | NA | Chi² = 7.09, df = 4 (P = 0.131), I^2^ = 44% |
|  | High risk of CVD | 1 | 620 | 0.02 (-0.02, 0.05), NA |  |
|  | T2DM | 0 | 0 | NA |  |
|  | Healthy | 3 | 266 | 0.00 (-0.01, 0.01), 0% |  |
|  | Hypercholesterolemia/ dyslipidemia | 4 | 142 | -0.01 (-0.03, -0.00), 0% |  |
|  | Metabolic syndrome | 0 | 0 | NA |  |
|  | Multiple/ other | 2 | 82 | 0.00 (-0.00, 0.01), 57% |  |
|  | Overweight/ obesity | 2 | 83 | 0.00 (-0.00, 0.01), 0% |  |
|  | Prediabetes | 0 | 0 | NA |  |
| Nut type | Almond | 6 | 190 | 0.00 (0.00, 0.01), 0% | Chi² = 2.26, df = 3 (P = 0.521), I^2^ = 0% |
|  | Cashew | 0 | 0 | NA |  |
|  | Hazelnut | 0 | 0 | NA |  |
|  | Macadamia | 0 | 0 | NA |  |
|  | Mixed | 1 | 620 | 0.02 (-0.02, 0.05), NA |  |
|  | Peanut | 0 | 0 | NA |  |
|  | Pecan | 2 | 96 | -0.00 (-0.15, 0.15), 0% |  |
|  | Pistachio | 0 | 0 | NA |  |
|  | Walnut | 4 | 305 | 0.00 (-0.00, 0.00), 0% |  |
| Nut dose | <30g | 0 | 0 | NA | Chi² = 0.43, df = 1 (P = 0.514), I^2^ = 0% |
|  | 30-59 | 6 | 970 | 0.00 (0.00, 0.01), 0% |  |
|  | ≥60g | 7 | 241 | 0.00 (-0.00, 0.01), 0% |  |

Abbreviations: CAD, coronary artery disease; CI, confidence interval; CVD, cardiovascular disease; df, degree of freedom; NA, not applicable
