# Supplementary figures and images for: Does Medication Status Impact the Effectiveness of Nuts in Altering Blood Pressure and Lipids? A Systematic Review and Meta-Analysis
Source: Nutr Rev. 2025 Apr 1;83(10):1843–60. doi: 10.1093/nutrit/nuaf033 (PMC12422015; doi:10.1093/nutrit/nuaf033)

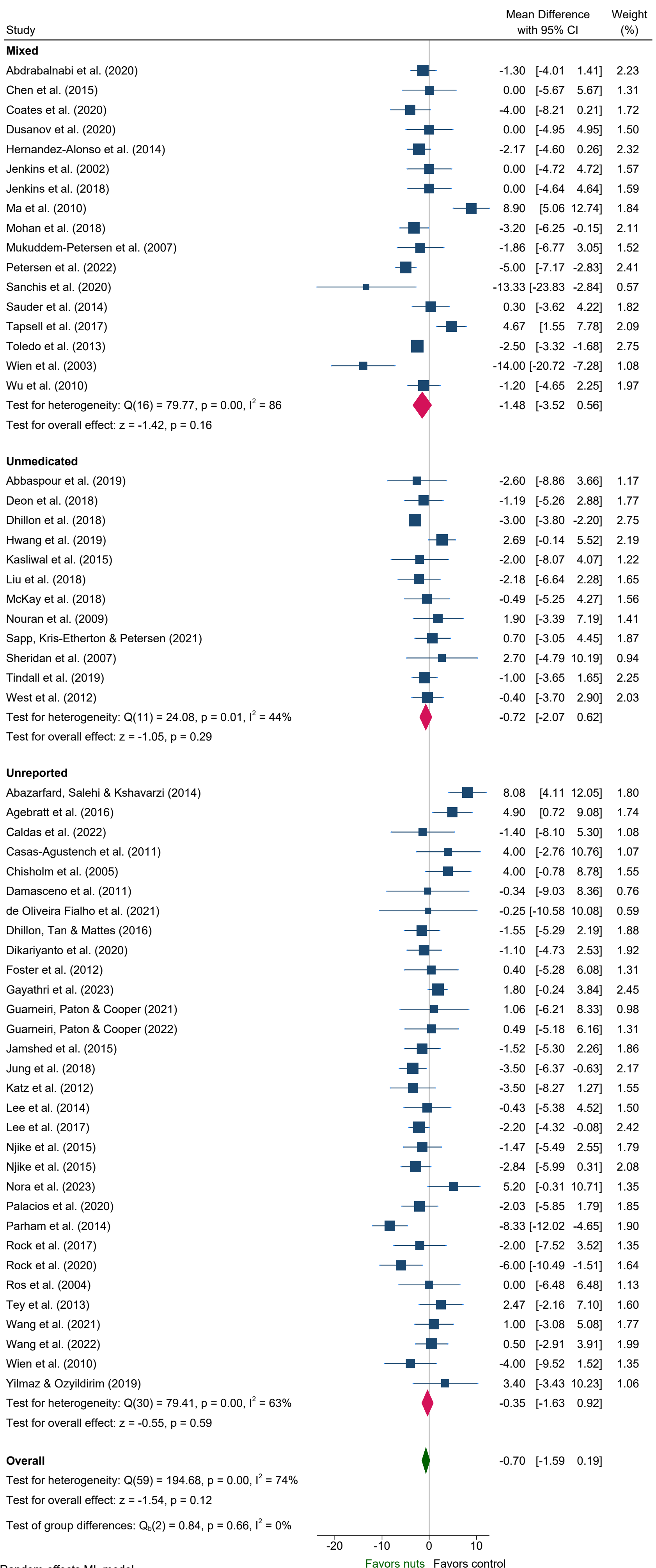

Supplement: nuaf033_Supplementary_Data [file nuaf033_supplementary_data.zip › Supplementary Figure 1 SBP.pdf]

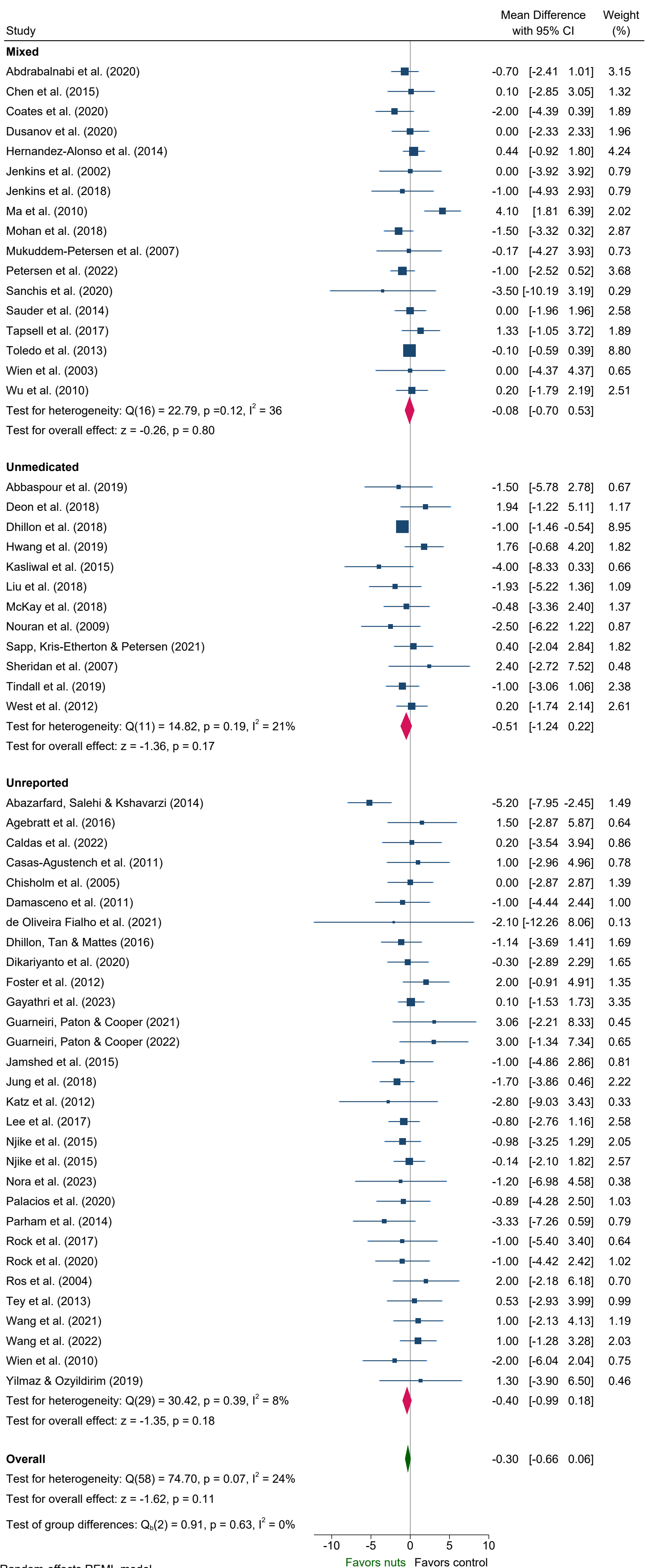

Supplement: nuaf033_Supplementary_Data [file nuaf033_supplementary_data.zip › Supplementary Figure 2 DBP.pdf]

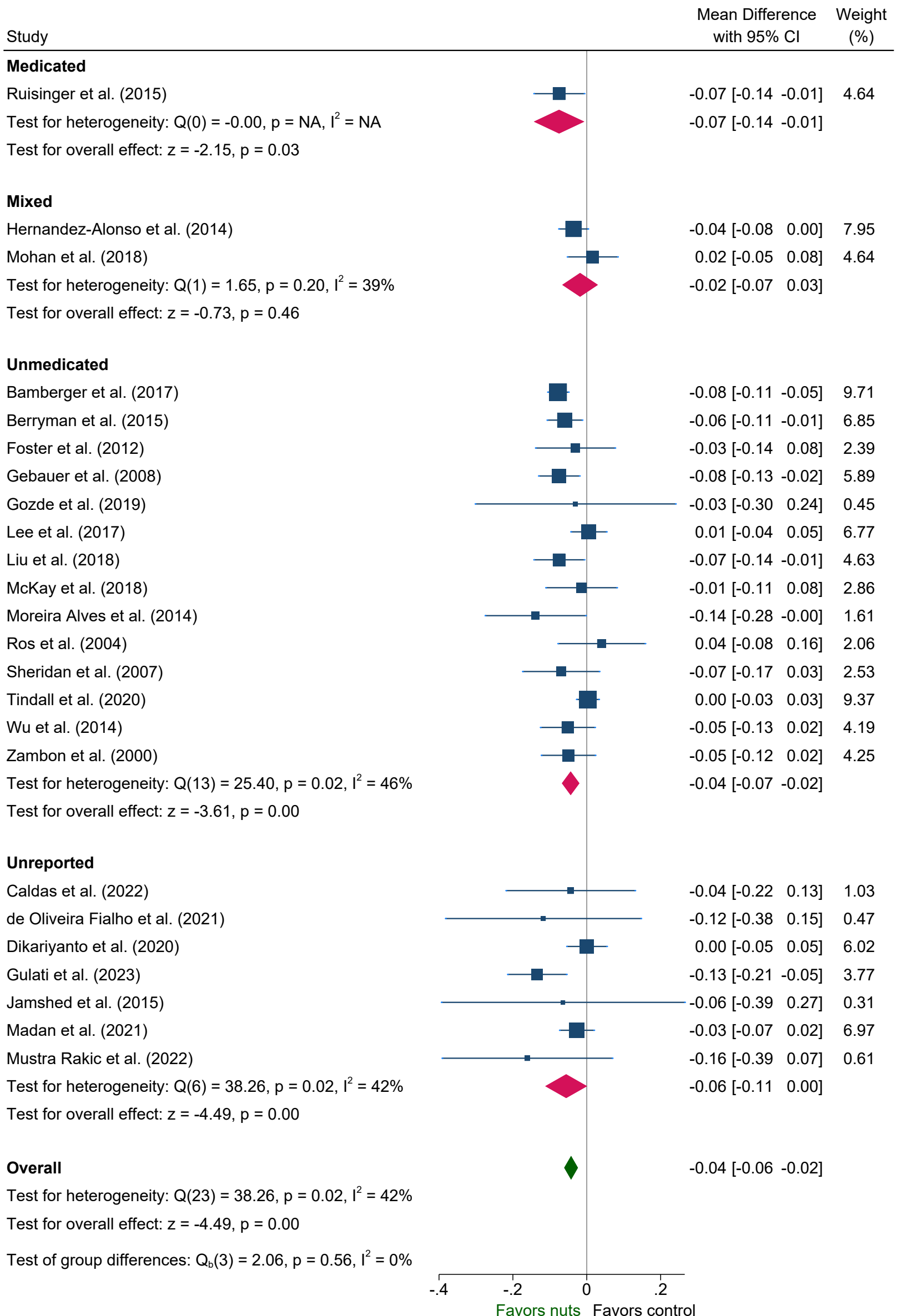

Supplement: nuaf033_Supplementary_Data [file nuaf033_supplementary_data.zip › Supplementary Figure 4 VLDL-C.pdf]

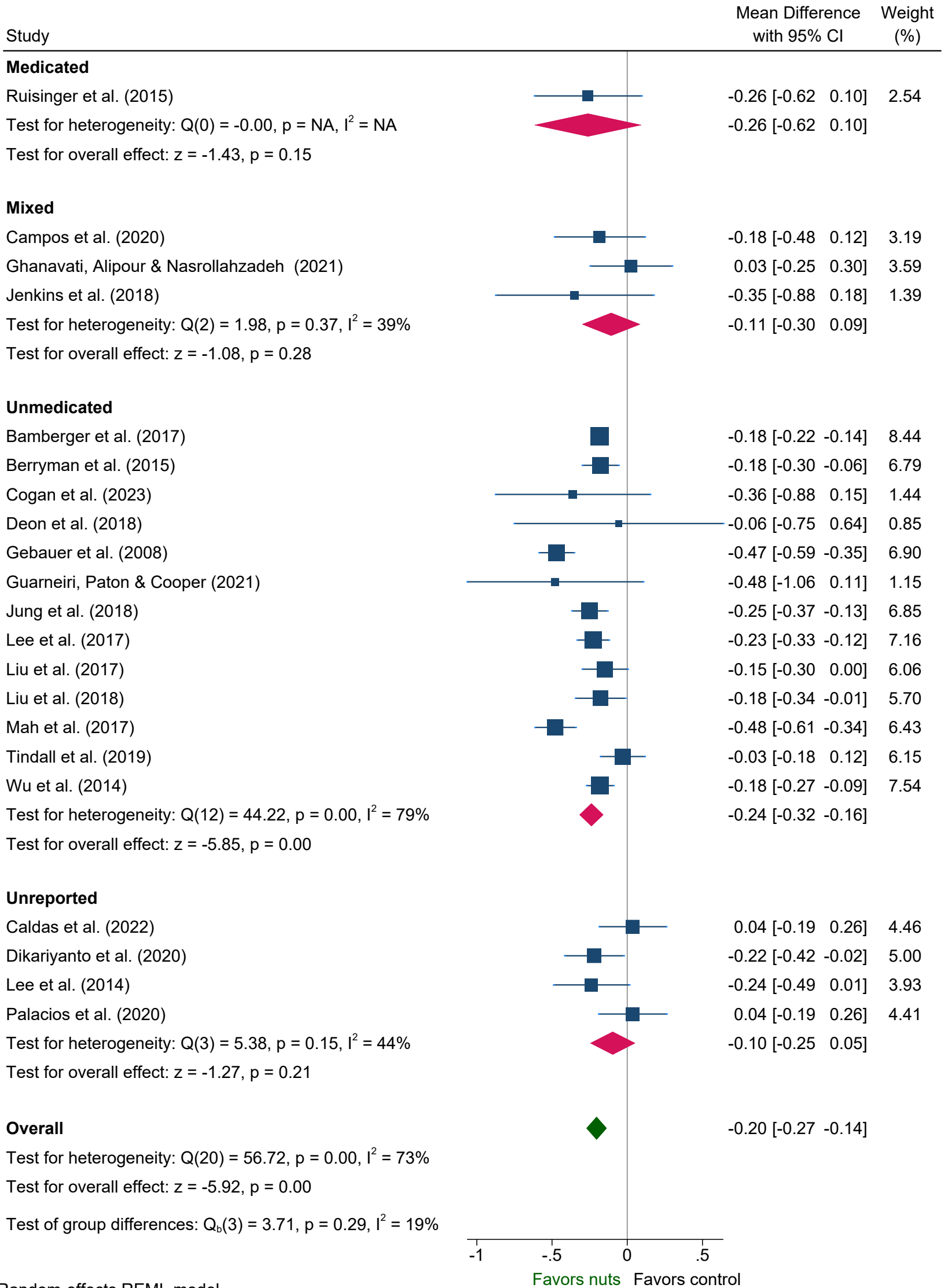

Supplement: nuaf033_Supplementary_Data [file nuaf033_supplementary_data.zip › Supplementary Figure 5 Non HDL-C.pdf]

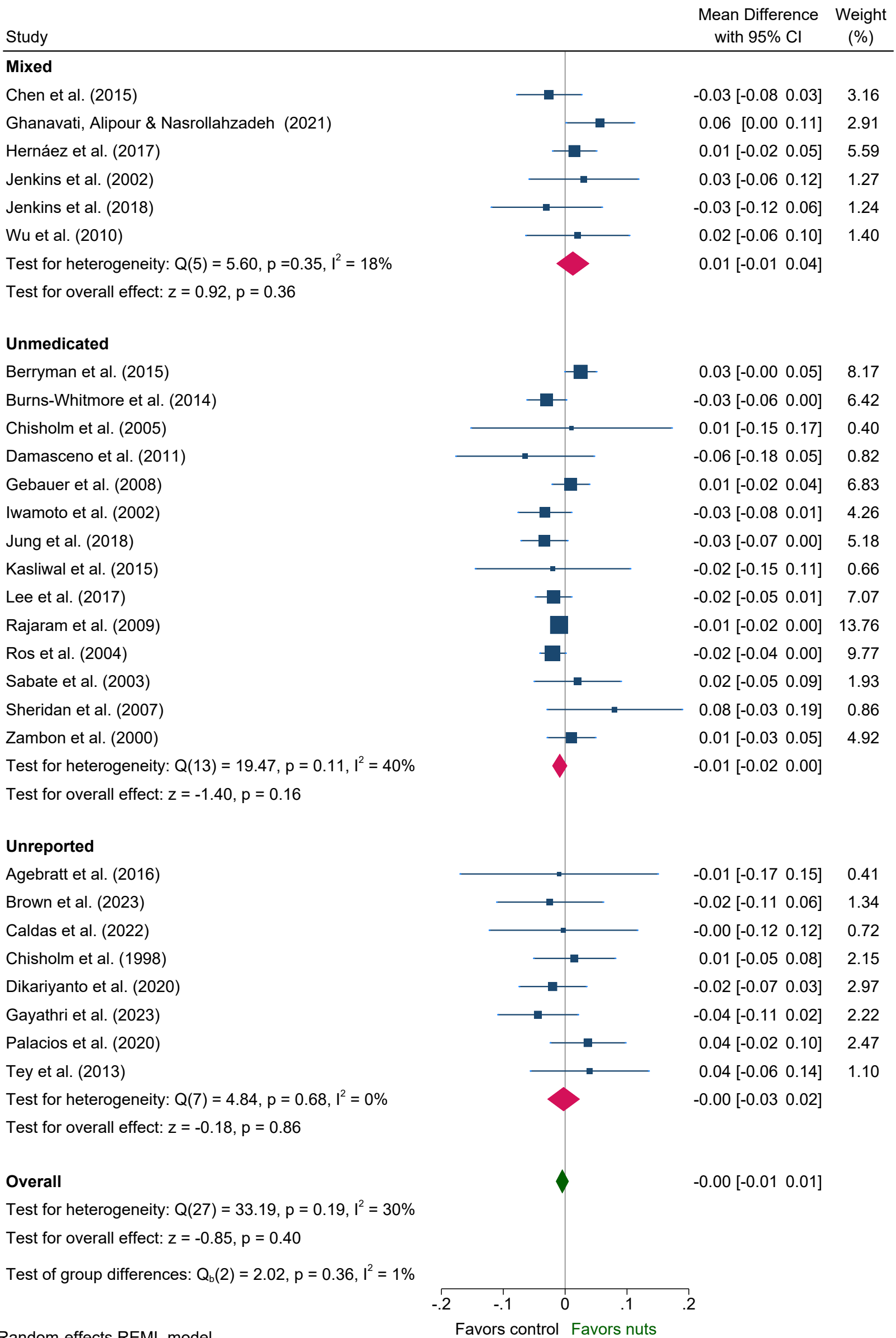

Supplement: nuaf033_Supplementary_Data [file nuaf033_supplementary_data.zip › Supplementary Figure 6 ApoA.pdf]

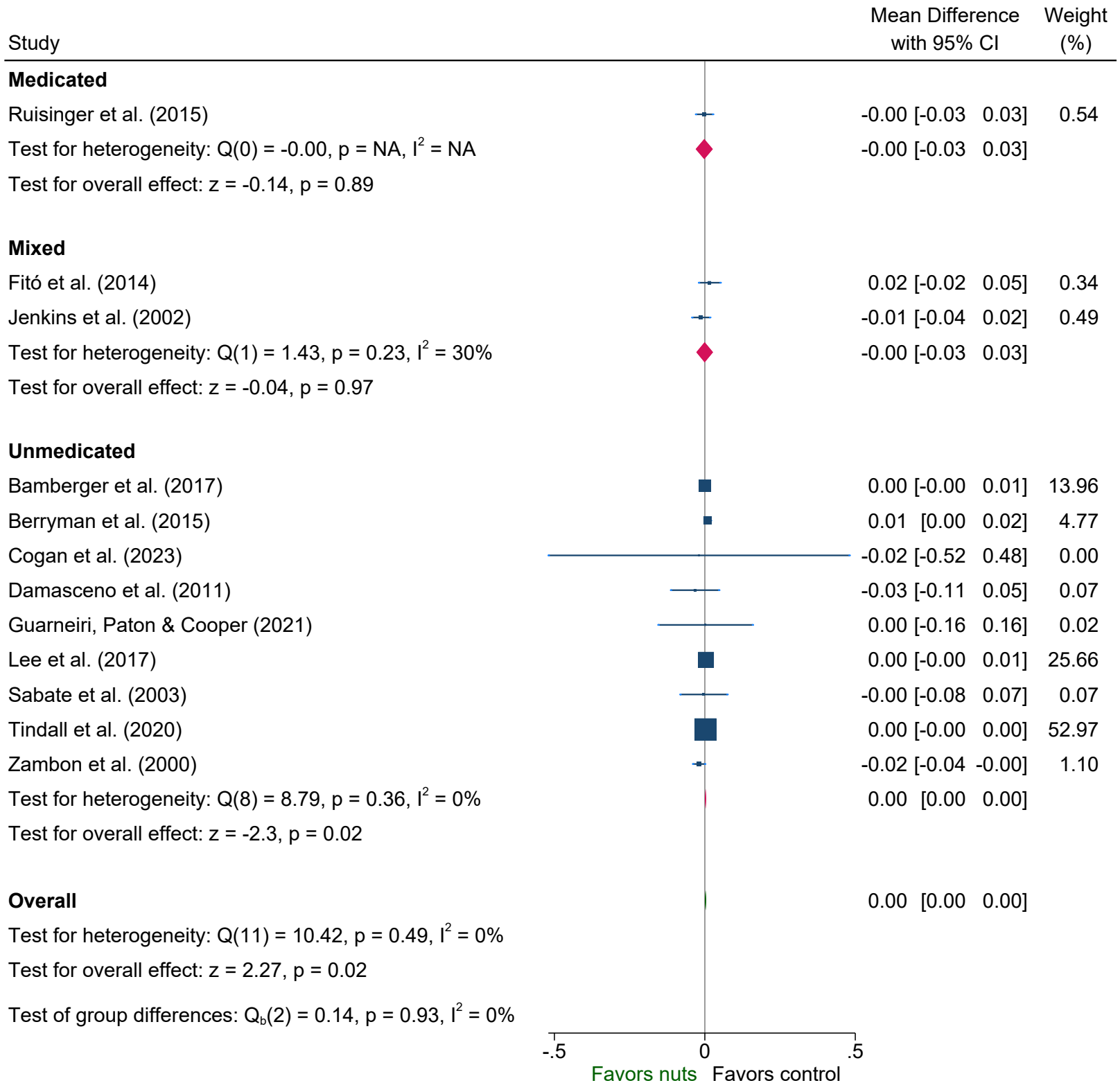

Supplement: nuaf033_Supplementary_Data [file nuaf033_supplementary_data.zip › Supplementary Figure 7 Lpa.pdf]

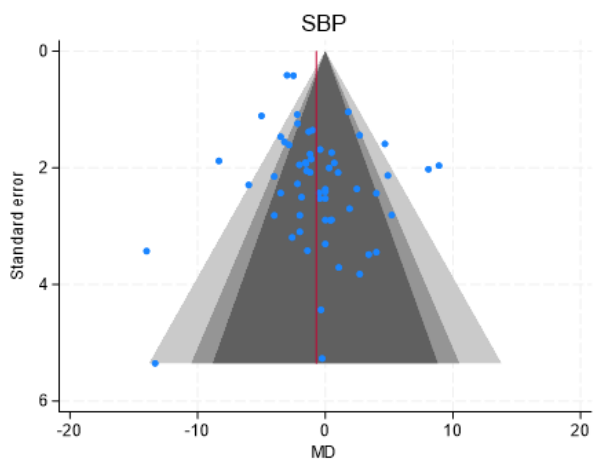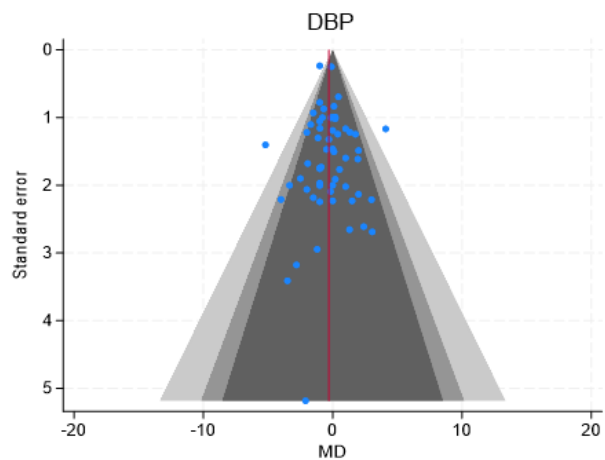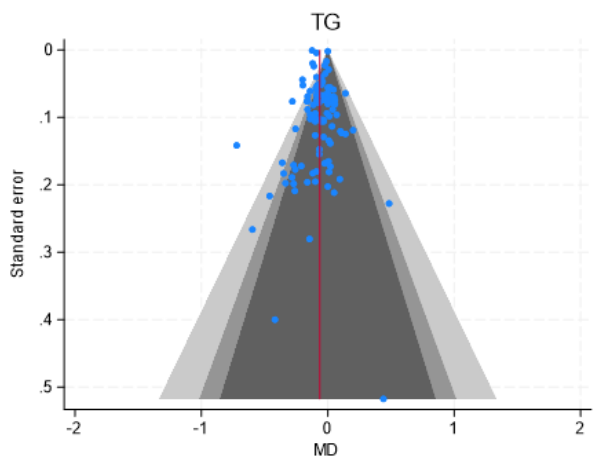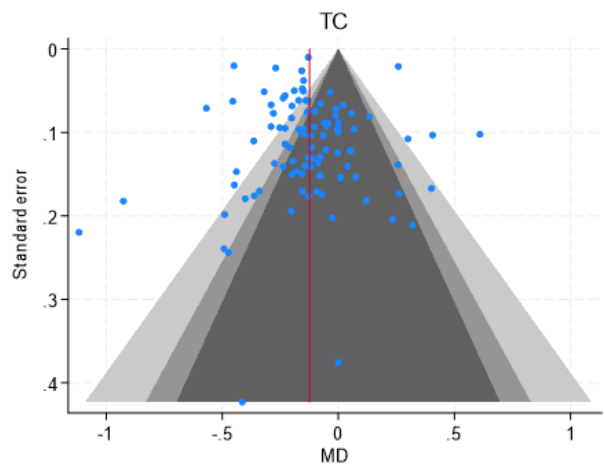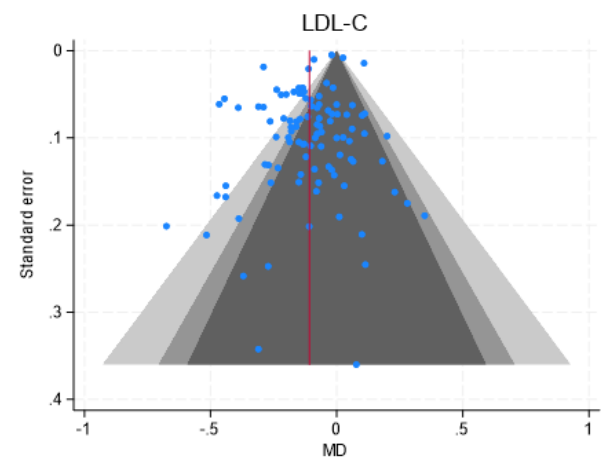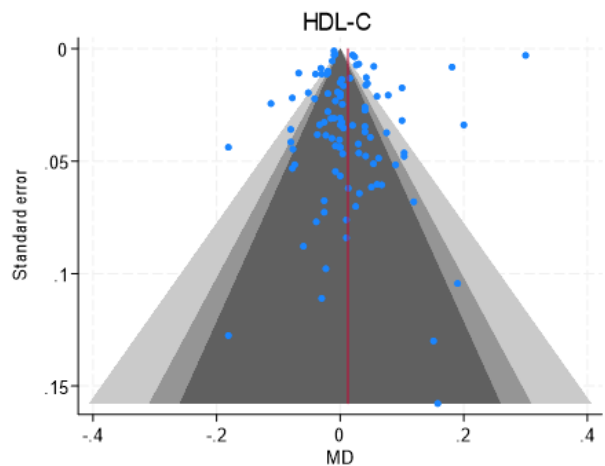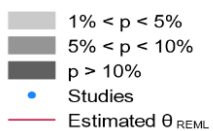

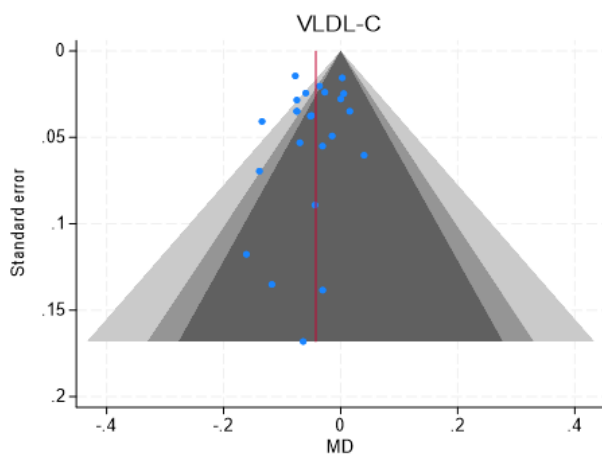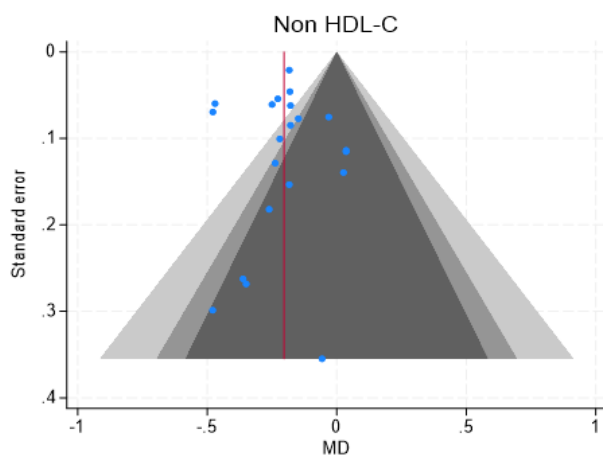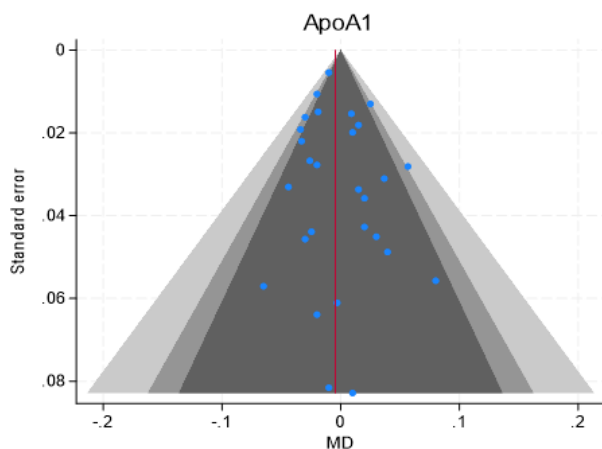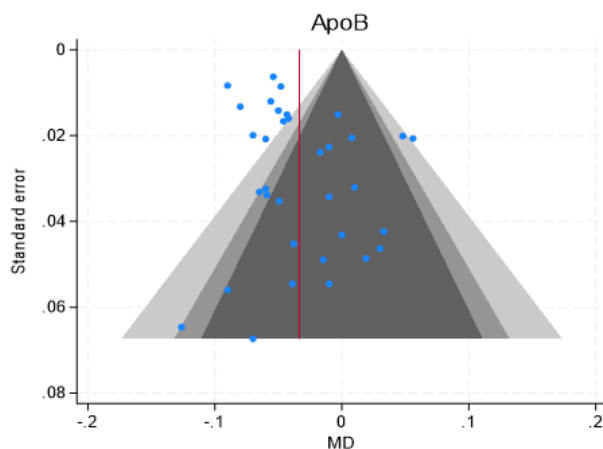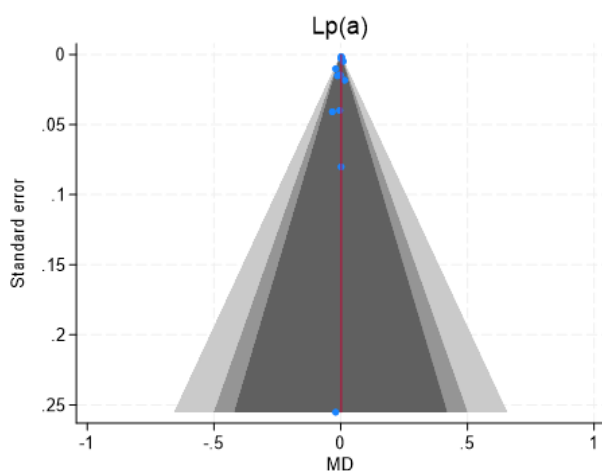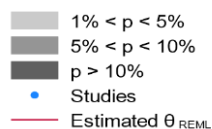

Supplement: nuaf033_Supplementary_Data [file nuaf033_supplementary_data.zip › Supplementary Figure 8_Funnel plots.pdf]
